# Site-specific encoding of photoactivity and photoreactivity into antibody fragments

**DOI:** 10.1038/s41589-022-01251-9

**Published:** 2023-02-16

**Authors:** Thomas Bridge, Udo Wegmann, Jason C. Crack, Kate Orman, Saher A. Shaikh, William Farndon, Carlo Martins, Gerhard Saalbach, Amit Sachdeva

**Affiliations:** 1 School of Chemistry, University of East Anglia, Norwich, UK; 2 Proteomics Facility, The John Innes Centre, Norwich, UK

**Keywords:** Protein design, Cancer therapy

## Abstract

Design of biomolecules that perform two or more distinct functions in response to light remains challenging. Here, we have introduced concurrent photoactivity and photoreactivity into an epidermal growth factor receptor (EGFR)-targeting antibody fragment, 7D12. This was achieved by site-specific incorporation of photocaged tyrosine (pcY) for photoactivity and *p*-benzoyl-ʟ-phenylalanine (Bpa) for photoreactivity into 7D12. We identified a position for installing Bpa in 7D12 that has minimal effect on 7D12–EGFR binding affinity in the absence of light. Upon exposure to 365-nm light, this Bpa-containing 7D12 mutant forms a covalent bond with EGFR in an antigen-specific manner. We then developed a method for site-specific incorporation of pcY and Bpa at two distinct sites in 7D12. Finally, we demonstrated that in the absence of light, this pcY- and Bpa-containing mutant of 7D12 does not bind to EGFR, but irradiation with 365-nm light activates (1) specific binding and (2) covalent bond formation with EGFR.

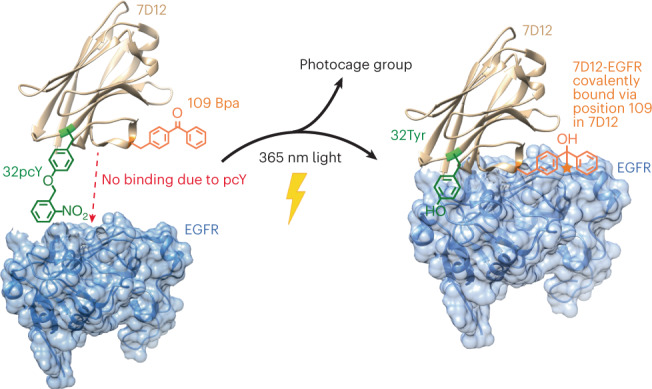

## Abstract (web summary)

Development of a generalized method for dual site-specific incorporation of nonnatural photocaged and photoreactive amino acids into proteins expressed in live cells enabled engineering of a photoreactive photoactive antibody fragment.

## Main

Applications of antibodies depend upon their specific binding to antigens, binding that is mediated by interactions, such as electrostatics, van der Waals, hydrophobic and hydrogen bonding, that are susceptible to changes in the microenvironment^1,2^. Replacing these noncovalent interactions with a covalent bond while concurrently modulating the binding in response to an external stimulus can further expand the applications of antibodies.

Several antibodies and antibody fragments have been previously developed for the treatment of various diseases, including cancer^3,4^. These antibodies bind to cell surface receptors expressed at higher levels on cancer cells, addressing a major challenge of selective cell targeting in cancer therapy. Although full-length antibodies have shown promise for treatment of several cancers, limited success has been demonstrated in eliminating solid tumors. Due to their large size, full-length antibodies are unable to diffuse deep into solid tumors^5^. In addition, it has been shown that high-affinity antibodies bind to the periphery of the tumor tissues, forming a barrier and preventing their further penetration^6^. Some studies in patients with cancer estimate that only 0.01% of the injected antibodies accumulate per gram of solid tumor tissue^7^. Small antibody fragments with low molecular weight can diffuse much deeper into tissues, presenting an excellent alternative to full-length antibodies. However, small antibody fragments have a low residence time in the body and often have a higher rate of dissociation (*k*_off_) from the target compared with full-length antibodies, limiting their clinical utility^8^. To address these challenges, antibody fragments are often multimerized^9,10^ and/or conjugated to larger proteins^11^, which increases the size of antibody fragments, again reducing their ability to penetrate into the tumor.

One solution to overcome the limitation of low residence time would be to replace the noncovalent interactions between the antibody fragment and its antigen with a covalent bond. In a notable effort, an affibody containing a photocrosslinker in its antigen binding region was shown to covalently link to its antigen and demonstrated higher accumulation on tumor tissues^12^. Another pioneering study involved developing affibodies containing a latent bioreactive amino acid in their antigen binding region that forms a covalent bond with the target antigen by proximity-dependent reaction without any external impetus^13^. However, the former had substantially lower binding affinity compared with its wild-type (wt) counterpart and thus, requires using a high concentration for efficient initial binding, while the latter could react with target antigen expressed on healthy cells causing side effects.

Although antibody-based therapeutics are more selective than several cytotoxic small molecule drugs used for cancer treatment, they can cause cardiac toxicity and skin reactions^14^. These side effects are due to the binding of the antibody to its receptor antigen expressed on healthy cells. This challenge could be addressed by activating antibody–antigen binding in the tumor microenvironment. One notable example in this direction is the development of antibodies containing an inhibitory N-terminal domain that is removed by tumor-specific proteases^15^. However, this approach would be difficult to extend to antibody fragments whose N terminus is not involved in antigen binding. We and others have also developed light-activated antibody fragments either by site-specific installation of photocaged functional groups or by introducing light-responsive proteins into antibodies^16–18^. In principle, such antibodies could be activated at the site of tumors using surgically implanted biocompatible light-emitting diodes (LEDs)^19^, thereby reducing the side effects of antibody-based therapeutics.

A method that allows covalent linking of antibody fragments to specific tumor cells while activating the binding at the site of the tumor using one external impetus, such as light, without substantial change in the size of the antibody fragment could (1) reduce the side effects of antibody fragments, (2) reduce the *k*_off_ of antibody fragments, (3) make the binding between the antibody fragment and the receptor less sensitive to the dynamic environment of tumor cells, (4) retain the high penetration of antibody fragments into tumor tissues and (5) allow user-defined control over antibody–antigen binding and its affinity. This could be achieved by engineering a photoreactive amino acid and a photocaged amino acid in the antigen binding region of an antibody fragment. The photoreactive amino acid should be positioned to allow light-promoted covalent bond formation with the antigen without inhibiting binding, whereas the photocaged amino acid should be positioned such that it inhibits antibody–antigen binding in the absence of light and binding is restored upon irradiation with light. Although genetic code expansion has allowed site-specific incorporation of multiple distinct noncanonical amino acids (ncAAs) in model proteins^20^, site-specific incorporation of multiple ncAAs into antibody fragments remains challenging, with only a few examples known^21^. There is no known example of a protein that has been conferred with concurrent photoactivity and photoreactivity.

We had earlier identified positions for introducing photoactivity in an antibody fragment, 7D12 (ref. ^16^). 7D12 is a single-domain antibody fragment that specifically binds to the epidermal growth factor receptor (EGFR) and is a drug candidate for the treatment of EGFR-positive cancers^10,22^. We had demonstrated that the presence of photocaged tyrosine (pcY) at position 32 in 7D12 inhibits its binding to EGFR, and the binding is restored upon irradiation with 365-nm light. In the current study, we first identified a position for introducing photoreactivity into 7D12 by site-specific incorporation of a photoreactive amino acid, *p*-benzoyl-ʟ-phenylalanine (Bpa). Subsequently, we developed a generalized approach to site-specifically incorporate pcY and Bpa into proteins. Finally, we demonstrated that the site-specific incorporation of pcY and Bpa into 7D12 can allow its activation and covalent ligation to its target, EGFR, upon irradiation with 365-nm light.

## Results

### Development of a high-affinity photoreactive 7D12 mutant

Site-specific incorporation of ncAAs into proteins is achieved by assigning stop or quadruplet codons to ncAAs and supplying the cells with orthogonal ncAA-specific aminoacyl-transfer RNA synthetase (aaRS)/transfer RNA (tRNA) pairs^23^. In *Escherichia coli*, evolved mutants of the *Methanocaldococcus jannaschii* tyrosyl-tRNA synthetase (*Mj*RS)/*Mj*tRNA pair and the *Methanosarcina* pyrrolysyl-tRNA synthetase (PylRS)/tRNA pair have been employed extensively to genetically encode several ncAAs^23,24^. An *Mj*RS/*Mj*tRNA pair has been previously evolved for site-specific incorporation of Bpa^25^. To develop photoreactive 7D12, we first used this mutant *Mj*RS(Bpa)/*Mj*tRNA pair to incorporate Bpa in 7D12. Bpa was selected for multiple reasons: (1) it undergoes photocrosslinking at ~365-nm light, the same wavelength as for light-mediated decaging of pcY; (2) Bpa preferentially reacts with the C–H bond and does not require any specific amino acid in EGFR to allow photocrosslinking; and (3) Bpa can reversibly and repeatedly be excited with ~365-nm light, facilitating excellent crosslinking efficiency^26^.

**Fig. 1:**
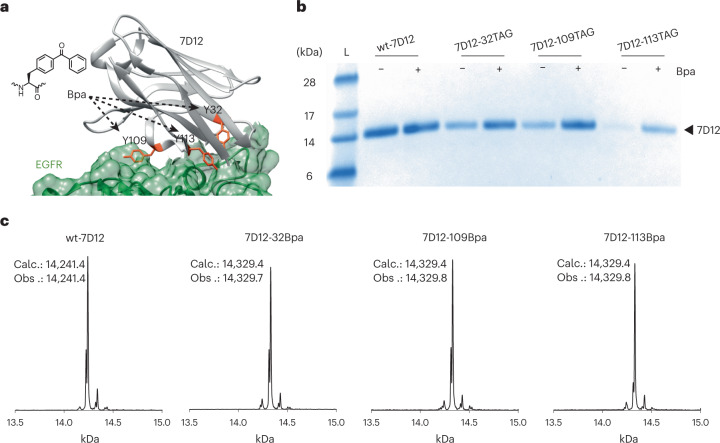
Site-specific incorporation of Bpa in a single-chain antibody fragment, 7D12. **a**, Crystal structure of 7D12 (gray)–EGFR domain III (green) complex (Protein Data Bank ID: 4KRL). Residues Y32, Y109 and Y113 (orange) in the antigen binding pocket of 7D12 were replaced with Bpa. **b**, Expression of wt-7D12 and its three amber mutants (viz. 32TAG, 109TAG and 113TAG) without and with Bpa. Comparison of band intensities for amber mutants with wt-7D12 shows efficient incorporation of Bpa. Full-length protein expressed for negative samples (−Bpa) indicates that *Mj*RS(Bpa) might incorporate canonical amino acids in the absence of Bpa. The lane marked L is the Invitrogen SeeBlue Plus2 Pre-stained Protein Standard (catalog no. LC5925). These experiments were repeated three times with similar results. **c**, ESI-MS results for wt-7D12, 7D12-32Bpa, 7D12-109Bpa and 7D12-113Bpa demonstrate site-specific incorporation of Bpa for expression of amber mutants of 7D12 with Bpa (see Supplementary Fig. 2 for MS data before deconvolution). Calc. is the calculated average molecular mass of the protein, and Obs. is the observed molecular mass from ESI-MS. Source data

The genes for the *Mj*RS(Bpa)/*Mj*tRNA_CUA_ pair were cloned into a suppressor plasmid, pULTRA^27^, forming pULTRA-Bpa (Methods and Supplementary Fig. 1). Three tyrosine residues in 7D12 (viz., 32, 109 and 113) at the binding interface of 7D12 and EGFR were targeted for replacement with Bpa (Fig. 1a). If Bpa is accommodated at any of these positions without inhibiting 7D12–EGFR binding, it would be close enough to EGFR to allow light-dependent covalent bond formation between 7D12 and EGFR. To express these 7D12 mutants, the *7D12* gene was provided by the pSANG10 plasmid as in our previous investigation^16^. Protein expression was performed in the presence and absence of Bpa (Fig. 1b and Methods). For the amber stop codon mutants of *7D12*, we observed a marked difference in the level of protein expressed with and without Bpa. Furthermore, ESI-MS analysis is consistent with site-specific incorporation of Bpa in 7D12 at positions 32, 109 and 113 (Fig. 1c and Supplementary Fig. 2); 2.6 mg of wt-7D12, 2.26 mg of 7D12-32Bpa, 0.57 mg of 7D12-109Bpa and 0.35 mg of 7D12-113Bpa per liter of culture were obtained after purification.

**Fig. 2:**
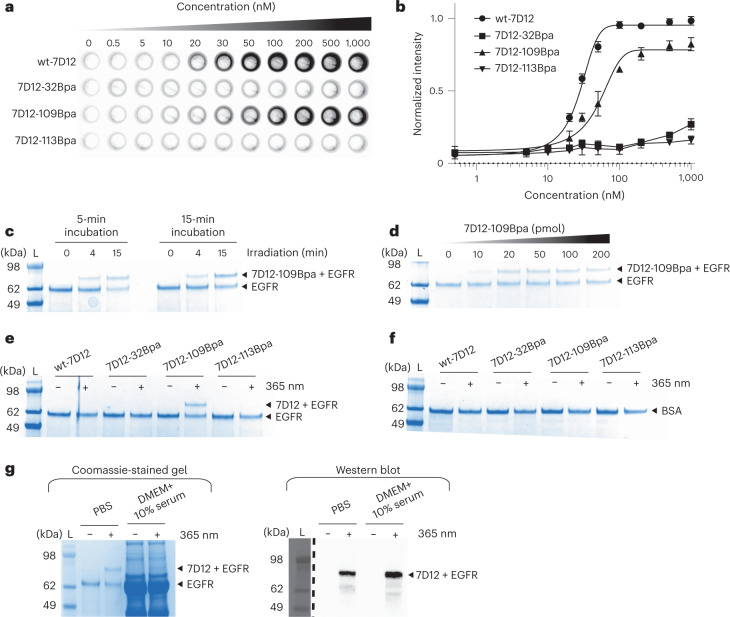
Development of the high-affinity photoreactive 7D12 mutant. **a**, On-cell binding assay demonstrates that the 7D12-109Bpa mutant binds to EGFR, whereas 7D12-32Bpa and 7D12-113Bpa show near-background binding. These experiments were performed in triplicates (Extended Data Fig. 1). **b**, Chemiluminescence intensities from on-cell binding experiments were quantified using the CLARIOstar plate reader. Normalized intensities were plotted against the concentration of 7D12, where the *x* axis is in log_10_ scale. Each point in the graph represents mean values of normalized intensities ± s.d., designated as the error bar, from three replicates. Data were fitted to the sigmoidal nonlinear equation using GraphPad to obtain binding affinity values (*K*_d_). For wt-7D12 and 7D12-109Bpa, *K*_d_ was estimated to be 27 (±1.5) nM and 48 (±7.2) nM, respectively (Extended Data Fig. 1). For wt-7D12 and 7D12-109Bpa, lines show the fitting trace. For 7D12-32Bpa and 7D12-113Bpa, lines show connection between individual points. **c**, Incubation time before irradiation has little effect on photocrosslinking between 7D12-109Bpa and EGFR, whereas with an increase in irradiation time, photocrosslinking efficiency increases from 17% at 5-min irradiation to 46% at 15-min irradiation (Extended Data Fig. 2). These experiments were repeated twice with similar results. **d**, Photocrosslinking efficiency saturates above 100 pmol of 7D12-109Bpa (Extended Data Fig. 3). These experiments were repeated twice with similar results. **e**, Photocrosslinked product was observed only with 7D12-109Bpa, demonstrating that photocrosslinking requires Bpa at position 109 (Extended Data Fig. 4). These experiments were repeated twice with similar results. **f**, No photocrosslinking was observed between BSA and Bpa-containing 7D12 mutants (Extended Data Fig. 5). These experiments were repeated twice with similar results. **g**, The left panel shows Coomassie-stained gel demonstrating successful photocrosslinking of 7D12-109Bpa to sEGFR in PBS. For the same reaction in serum-containing media, bands for sEGFR and photocrosslinked product are not clear due to serum proteins. The right panel shows an anti-His_6_ antibody western blot that detects the C-terminal His_6_ tag on 7D12-109Bpa. The bands show the sEGFR–7D12-109Bpa complex demonstrating successful photocrosslinking in serum-containing media (Extended Data Fig. 6). These experiments were repeated twice with similar results. For gel images, lanes marked L are the Invitrogen SeeBlue Plus2 Pre-stained Protein Standard (catalog no. LC5925). Source data

**Extended Data Fig. 1:**
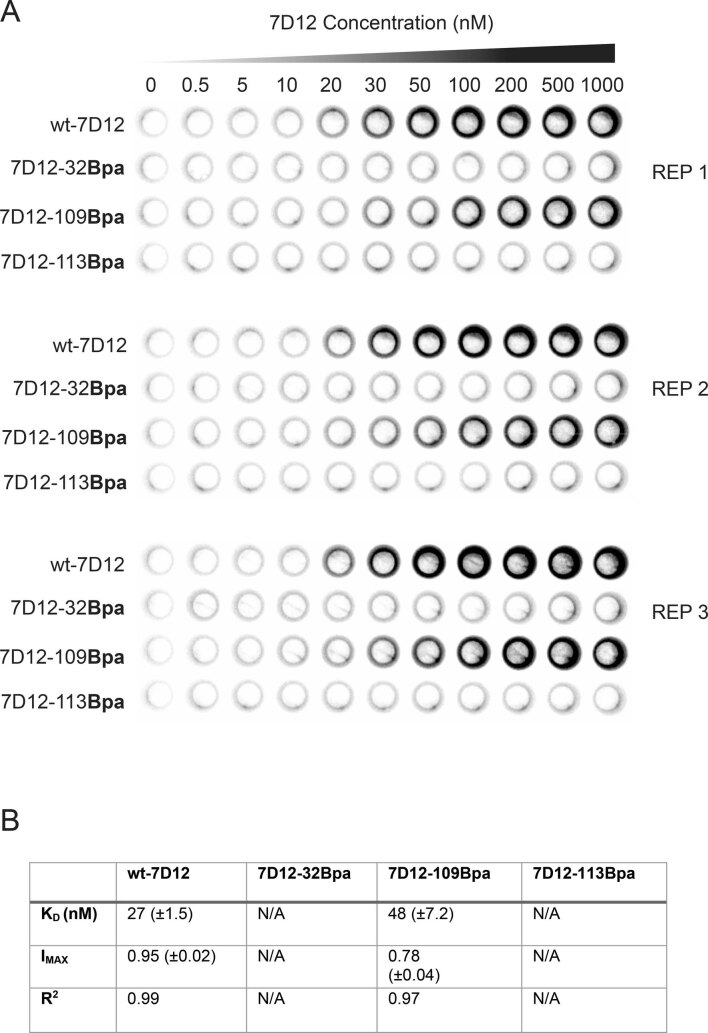
On-cell binding assay to measure the binding affinity of wt-7D12, 7D12-32Bpa, 7D12-109Bpa, and 7D12-113Bpa to Epidermal Growth Factor Receptor (EGFR) expressed on the surface of A431 cells. (A) Results demonstrate that site-specially incorporated Bpa at position 109 in 7D12 has little effect on 7D12-EGFR binding affinity. To ensure reproducibility, experiments were performed in triplicates represented as REP 1, REP 2 and REP 3. (B) Quantitative assessment of binding between Bpa-containing 7D12 mutants and EGFR. Chemiluminescence intensities obtained from on-cell binding experiments for wt-7D12, 7D12-32Bpa, 7D12-109Bpa, and 7D12-113Bpa were quantified using CLARIOstar plate reader. For each plate the maximum value of intensity was normalized to 1 and the minimum normalized to zero. normalization is performed to ensure that data between replicates could be compared. The normalized intensity was plotted against concentration of 7D12. The data was fitted to sigmoidal nonlinear equation using GraphPad as shown in Fig. 2b. K_D_ is the concentration of the 7D12, where chemiluminescence intensity is half of the maximum chemiluminescence intensity. I_MAX_ is the normalized maximum chemiluminescence intensity at saturation. Values shown in brackets denote the standard deviation (s.d.) from the mean value of three replicates. Source data

Next, we assessed the binding of Bpa-containing mutants of 7D12 to EGFR expressed on the surface of cancer cells using our previously developed on-cell assay^16^ (Methods). The results demonstrate that the presence of Bpa at positions 32 and 113 in 7D12 inhibits its binding to EGFR (Fig. 2a and Extended Data Fig. 1a). However, Bpa at position 109 decreases the binding affinity by only twofold, with the dissociation constant (*K*_d_) of wt-7D12 and 7D12-109Bpa to EGFR estimated to be 27 (±1.5) nM and 48 (±7.2) nM, respectively (Fig. 2b and Extended Data Fig. 1b). Previous investigations focused on introducing photoreactive functional groups at the antibody–antigen binding interface have often led to a 10- to 100-fold decrease in *K*_d_ values^12,28^.

Subsequently, we evaluated if Bpa at position 109 allows for light-dependent covalent bond formation between 7D12 and EGFR. In vitro experiments were performed by incubating 7D12-109Bpa with the extracellular domain of EGFR (sEGFR), followed by irradiation with 365-nm light (Methods). Samples were analyzed by denaturing SDS–PAGE, and a band higher than sEGFR indicates the covalently linked 7D12–EGFR complex. For these experiments, the amount of sEGFR was fixed at 10 pmol to be able to see a clearly defined band on SDS–PAGE gels.

**Extended Data Fig. 2:**
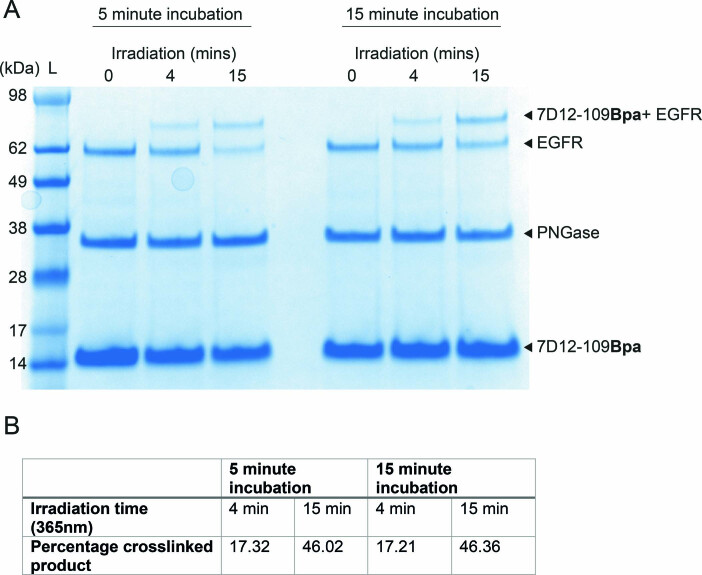
Effect of incubation time prior to irradiation, and irradiation time on photocrosslinking efficiency between 7D12-109Bpa and EGFR assessed using denaturing SDS–PAGE. (A) In the Coomassie stained gel image, the band higher than EGFR is photocrosslinked 7D12-109Bpa + EGFR complex. This is the full gel image for data shown in Fig. 2c. Lane marked L is the Invitrogen SeeBlue Plus2 Pre-stained Protein Standard (Catalog no. LC5925). These results demonstrate that i) incubation time prior to irradiation has little effect on photocrosslinking efficiency, and ii) irradiation time has a profound effect on photocrosslinking efficiency. (B) Percentage crosslinked product in different lanes is calculated by measuring the intensity of the band corresponding to 7D12-109Bpa + EGFR and EGFR. Source data

**Extended Data Fig. 3:**
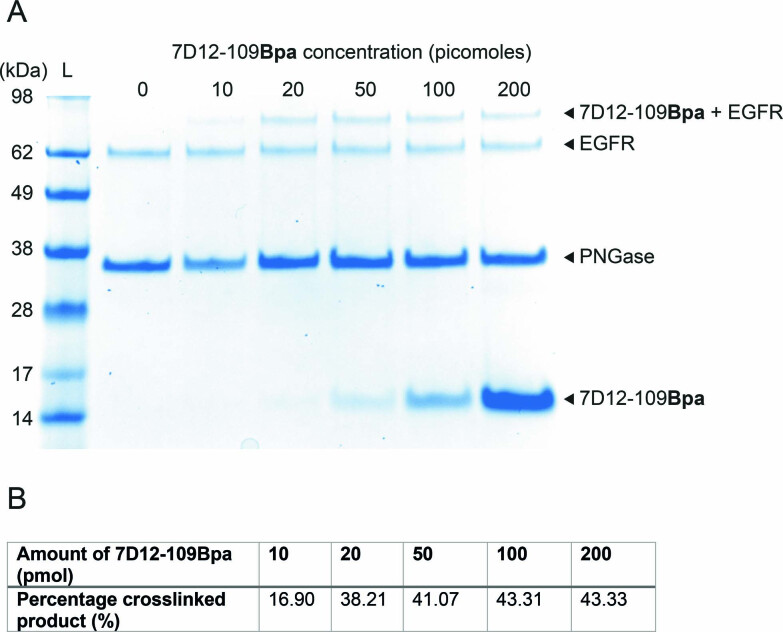
Effect of the amount of 7D12-109Bpa on the photocrosslinking efficiency between 7D12-109Bpa and EGFR assessed using denaturing SDS–PAGE. For these experiments, the amount of EGFR is 10 picomoles, incubation time prior to irradiation is 5 min, irradiation time with 365 nm light is 10 min, and the amount of 7D12-109Bpa is varied from 0 to 200 picomoles. (A) In the Coomassie stained gel image, the band higher than EGFR is photocrosslinked 7D12-109Bpa + EGFR complex. This is the full gel image for data shown in Fig. 2d. Lane marked L is the Invitrogen SeeBlue Plus2 Pre-stained Protein Standard (Catalog no. LC5925). These results demonstrate that the photocrosslinking efficiency plateaus at 100 picomoles of 7D12-109Bpa. (B) Percentage crosslinked product at different amounts of 7D12-109Bpa calculated by measuring the intensity of the band corresponding to 7D12-109Bpa + EGFR and EGFR. Source data

We assessed the effect of incubation time and irradiation time on photocrosslinking efficiency. sEGFR was incubated with 10-fold excess of 7D12-109Bpa for 5 and 15 min, and the samples were irradiated for 0, 4 and 15 min. Incubation time before irradiation was seen to have a negligible effect on photocrosslinking efficiency. In contrast, as the irradiation time increased from 4 to 15 min, the percentage of photocrosslinked product increased from 17 to 46%, respectively (Fig. 2c and Extended Data Fig. 2). As long irradiation times could be toxic to cells, we also assessed the viability of A431 cells irradiated with 365-nm light for 0–15 min (Supplementary Fig. 3 and Methods). Over 90% of A431 cells were found to be viable after a 10-min exposure to 365-nm light. Thus, the irradiation time was fixed to 10 min. Next, we evaluated the effect of the relative amounts of 7D12-109Bpa to sEGFR on the photocrosslinking efficiency. Ten picomoles of sEGFR was incubated with 0–200 pmol of 7D12-109Bpa for 5 min, and samples were irradiated with 365 nm for 10 min. Photocrosslinking efficiency saturates at 43% when the concentration of 7D12-109Bpa is 10-fold or higher compared with sEGFR (Fig. 2d and Extended Data Fig. 3), similar to other photoreactive proteins^12,25^. From these results, we concluded that the optimal conditions to assess photocrosslinking are, in a 10-μl reaction, 10 pmol of sEGFR, 100 pmol of 7D12 mutants, 5 min of incubation time before irradiation and irradiation time of 10 min.

**Extended Data Fig. 4:**
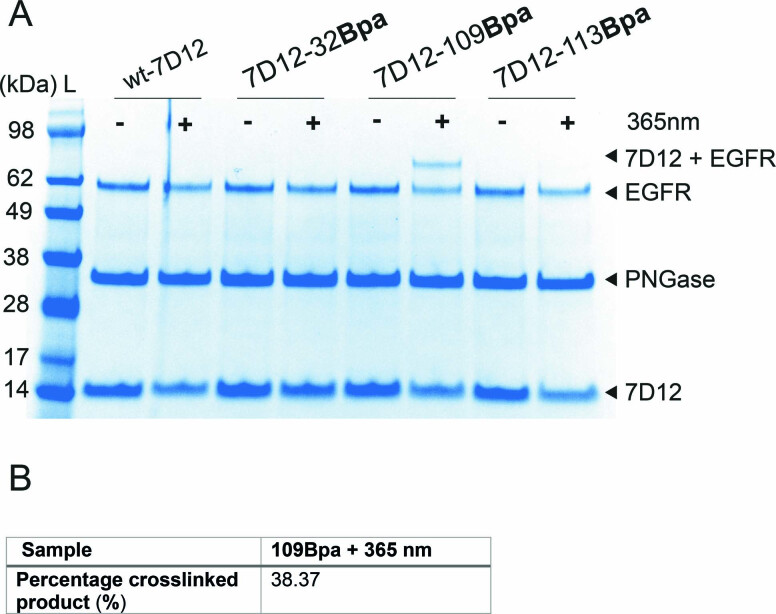
Light dependent covalent bond formation between 7D12 and EGFR is antibody-specific. For these experiments, the amount of EGFR is 10 picomoles, incubation time prior to irradiation is 5 min, irradiation time with 365 nm light is 10 min, and the amount of 7D12 and its Bpa-containing mutants is 100 picomoles. (A) In the Coomassie stained gel image, the band higher than EGFR is photocrosslinked 7D12-109Bpa + EGFR complex. This is the full gel image for data shown in Fig. 2e. Lane marked L is the Invitrogen SeeBlue Plus2 Pre-stained Protein Standard (Catalog no. LC5925). These results demonstrate that the photocrosslinking occurs only for 7D12-109Bpa upon irradiation with 365 nm light. (B) Percentage crosslinked product calculated by measuring the intensity of the band corresponding to 7D12-109Bpa + EGFR and EGFR. These experiments were repeated twice with similar results. Source data

**Extended Data Fig. 5:**
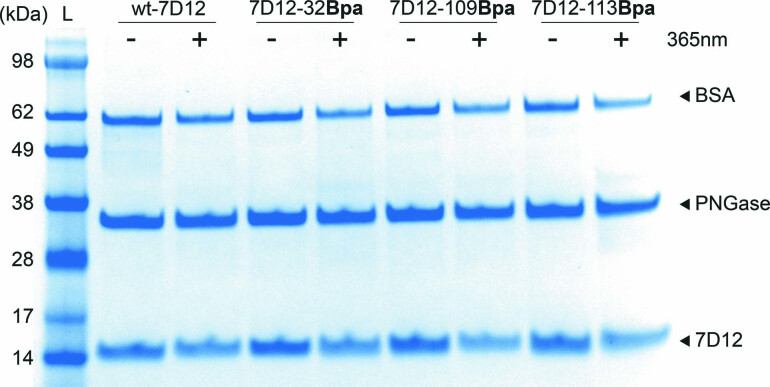
Light dependent covalent bond formation between 7D12 and EGFR is antigen-specific. No photocrosslinked product is observed when 7D12 and its Bpa-containing mutants are irradiated with 365 nm in the presence of Bovine Serum Albumin (BSA) and not with EGFR. For these experiments, the amount of BSA is 10 picomoles, incubation time prior to irradiation is 5 min, irradiation time with 365 nm light is 10 min, and the amount of 7D12 and its Bpa-containing mutants is 100 picomoles. This is the full gel image for data shown in Fig. 2 f. Lane marked L is the Invitrogen SeeBlue Plus2 Pre-stained Protein Standard (Catalog no. LC5925). These experiments were repeated twice with similar results. Source data

Next, we demonstrated that the light-dependent covalent bond formation between 7D12-109Bpa and EGFR is antibody-specific and antigen-specific. Photocrosslinking of sEGFR was performed with wt-7D12, 7D12-32Bpa, 7D12-109Bpa and 7D12-113Bpa using our optimized conditions. The crosslinked product was observed only for 7D12-109Bpa when irradiated with 365-nm light (Fig. 2e and Extended Data Fig. 4). The results demonstrate that light-dependent covalent bond formation between 7D12 and EGFR (1) requires site-specifically incorporated Bpa, as no covalent bond formation was observed between wt-7D12 and EGFR when irradiated with 365-nm light, and (2) requires specific binding between 7D12 and EGFR. 7D12-32Bpa and 7D12-113Bpa that do not bind to EGFR (Fig. 2a) are unable to form a covalent bond with EGFR upon irradiation with light. To test whether 7D12-109Bpa-EGFR bond formation is antigen specific, we also irradiated 7D12-109Bpa with an unrelated protein, BSA, that resulted in no crosslinked product (Fig. 2f and Extended Data Fig. 5).

**Extended Data Fig. 6:**
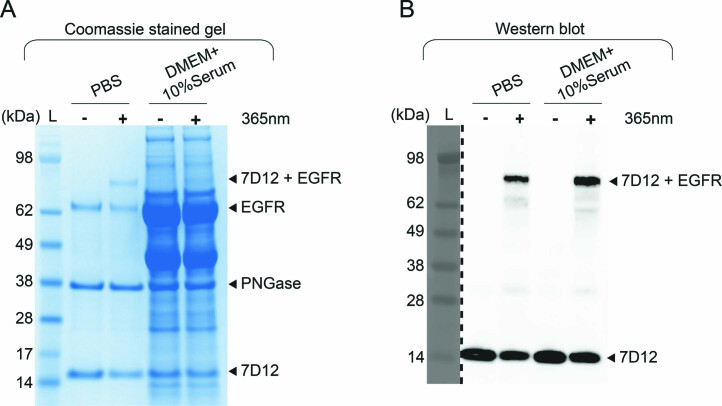
Photocrosslinking of 7D12-109Bpa with EGFR occurs in DMEM containing 10% serum. (A) The Coomassie stained gel demonstrates successful photocrosslinking of 7D12-109Bpa to EGFR in phosphate buffered saline (PBS). For the same reaction performed in serum containing media, bands corresponding to EGFR and photocrosslinked product are not clear on Coomassie stained gel due to the presence of other proteins in the serum. This is the full gel image for data shown in Fig. 2 g. Lane marked L is the Invitrogen SeeBlue Plus2 Pre-stained Protein Standard (Catalog no. LC5925). This experiment was repeated twice with similar results. (B) The anti-His_6_ antibody western blot detects the C-terminal His_6_ tag on 7D12-109Bpa. The photocrosslinked product, 7D12 + EGFR (top band in the blot) appears in lanes where 7D12-109Bpa/sEGFR in PBS or in serum containing media are irradiated with 365 nm light, demonstrating successful photocrosslinking under both conditions. The lower band corresponds to 7D12-109Bpa and serves as a control demonstrating detection of 7D12-109Bpa in all samples. The image was acquired using GE ImageQuant™ LAS 4000 gel imager. This is the full gel image for data shown in Fig. 2g. Lane marked L is the Invitrogen SeeBlue Plus2 Pre-stained Protein Standard (Catalog no. LC5925). These experiments were repeated twice with similar results. Source data

Furthermore, we demonstrated that photocrosslinking of 7D12-109Bpa can be performed in serum-containing media (Fig. 2g and Extended Data Fig. 6). 7D12-109Bpa was incubated with sEGFR in PBS as a control, similar to previous in vitro photocrosslinking experiments, or DMEM containing 10% serum (Methods). In the Coomassie-stained gel, the crosslinked product is observed for reaction in PBS but is masked by other proteins for reaction in serum-containing media (Fig. 2g). However, a western blot detecting the 7D12-109Bpa C-terminal His_6_ tag shows a band corresponding to the photocrosslinked sEGFR–7D12-109Bpa complex in both reactions, in PBS and in serum, demonstrating light-mediated crosslinking of 7D12-109Bpa to sEGFR in serum-containing media (Fig. 2g). We thus developed a high-affinity photoreactive 7D12 mutant, 7D12-109Bpa, that selectively forms a covalent bond with EGFR upon irradiation with light under biologically relevant conditions.

We also characterized the photocrosslinked 7D12-109Bpa–sEGFR complex using MS. The band corresponding to the photocrosslinked 7D12-109Bpa–sEGFR complex was excised from SDS–PAGE, destained and digested with trypsin/chymotrypsin. The digested sample was then analyzed by liquid chromatography with tandem MS (LC–MS/MS) (Methods). Peptide fragments corresponding to 7D12 (73% coverage) and sEGFR (62% coverage) were observed in the photocrosslinked 7D12-109Bpa–sEGFR complex analyzed by MS/MS (Supplementary Figs. 4 and 5).

Next, we aimed to develop a photoactive, photoreactive mutant of 7D12. We had earlier demonstrated that site-specific incorporation of pcY at position 32 in 7D12 inhibits its binding to EGFR, and irradiation with 365-nm light restores this binding^16^. Hence, we examined whether site-specific incorporation of Bpa at position 109 and pcY at position 32 could confer concurrent photoreactivity and photoactivity to 7D12.

Expression of proteins containing two distinct site-specifically incorporated ncAAs in live cells requires two mutually orthogonal aaRS/tRNA pairs, which are also orthogonal to the host aaRS/tRNA pairs. In addition, these aaRSs should be able to charge the tRNA exclusively with their corresponding ncAA in the presence of other ncAAs and canonical amino acids, which could be a potential challenge for the structurally similar pcY and Bpa^29,30^. In *E. coli*, *Methanosarcina barkeri* pyrrolysyl-tRNA synthetase (*Mb*PylRS)/*Mb*PyltRNA and *Mj*RS/*Mj*tRNA pairs have been shown to be orthogonal to each other^31,32^ and were thus chosen for site-specific incorporation of pcY and Bpa in 7D12 in the present study. To select which of the two pairs to employ for incorporation of pcY/Bpa, we first assessed the selectivity of the known *Mj*RS(Bpa) and *Mj*RS(pcY)^25,33^. Expression of *7D12-109TAG* mutant using these aaRSs was performed in the absence and presence of pcY or Bpa (Supplementary Fig. 6). For *Mj*RS(pcY), the full-length 7D12 was only observed when expression was performed with pcY. However, the *Mj*RS(Bpa) appears to be promiscuous and incorporates both Bpa and pcY into 7D12. Thus, for dual incorporation of pcY and Bpa in 7D12, we decided to employ the highly selective *Mj*RS(pcY) for the site-specific incorporation of pcY and the *Mb*PylRS for site-specific incorporation of Bpa into 7D12. Next, we embarked upon evolving an efficient and selective *Mb*PylRS for the site-specific incorporation of Bpa.

### Directed evolution of PylRS mutant for incorporation of Bpa

**Fig. 3:**
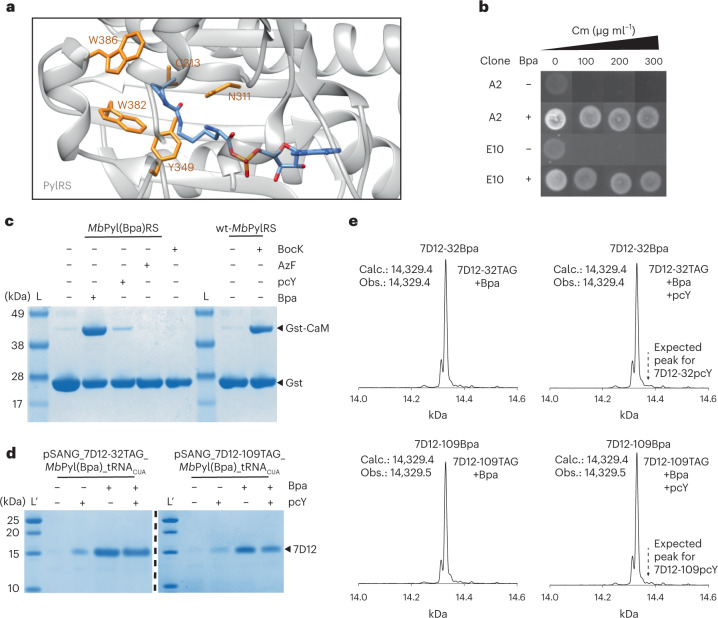
Development of an efficient and selective *Mb*PylRS/tRNA pair for site-specific incorporation of Bpa. **a**, Crystal structure of PylRS (gray) with adenylated pyrrolysine (blue; Protein Data Bank ID 2Q7H). Residues N311, C313, W382 and W386 (orange) in *Mb*PylRS were randomized to all combinations of amino acids for directed evolution experiments. **b**, To isolate Bpa-specific mutants of *Mb*PylRS, three rounds of directed evolution were performed, and subsequently, 192 clones were screened (Supplementary Fig. 7). Two clones, A2 and E10, survived on chloramphenicol concentration up to 300 μg ml^−1^ in the presence of 1 mM Bpa (A2 +Bpa and E10 +Bpa) but did not survive in the absence of Bpa on chloramphenicol concentration at and above 100 μg ml^−1^ (A2 −Bpa and E10 −Bpa). Both clones were the same and had the following mutations compared with the wild-type *Mb*PylRS: N311Q, C313T and W382A, in addition to the Y349F preprogrammed mutation. **c**, Expression of *gst-1TAG-cam* using *Mb*Pyl(Bpa)RS without ncAA, with 1 mM Bpa, 1 mM pcY, 1 mM AzF or 1 mM BocK. Control expression using wt-*Mb*PylRS that is known to efficiently incorporate BocK was also performed. Comparison of band intensities of full-length Gst-CaM demonstrates that newly evolved *Mb*Pyl(Bpa)RS is highly efficient and specific at incorporating Bpa (Supplementary Fig. 8). These experiments were repeated twice with similar results. **d**, Selective incorporation of Bpa at positions 32 and 109 in 7D12 using *Mb*Pyl(Bpa)RS. Comparison of band intensities for expression with Bpa (1 mM), pcY (1 mM), and both Bpa (1 mM) and pcY (1 mM) indicates selective incorporation of Bpa in the presence of pcY. These experiments were repeated twice with similar results. **e**, ESI-MS of amber mutants of *7D12* expressed using *Mb*Pyl(Bpa)RS in the presence of both Bpa (1 mM) and pcY (1 mM) demonstrates that Bpa is selectively incorporated. The dotted lines with arrows indicate expected molecular masses if pcY was incorporated (Supplementary Fig. 9). For gel images, lanes marked L are the Invitrogen SeeBlue Plus2 Pre-stained Protein Standard (catalog no. LC5925), and lanes marked Lʹ are the Thermo Scientific PageRuler Unstained Low Range Protein Ladder (catalog no. 26632). Source data

Four amino acid residues in the amino acid binding pocket of *Mb*PylRS (viz. N311, C313, W382 and W386) were randomized to all combinations of amino acids (Methods and Fig. 3a). In addition, the Y349F mutation that is known to improve the aminoacylation efficiency of *Mb*PylRS was introduced in the library^34^. Using this library, three rounds of alternating positive and negative selections were carried out to isolate Bpa-specific *Mb*PylRS mutants (Methods). Positive selection was performed in the presence of Bpa and allowed survival of cells containing *Mb*PylRS mutants that incorporate any amino acid in response to a TAG stop codon in the chloramphenicol acetyltransferase (*catA*) gene. The negative selection was performed in the absence of Bpa and assisted in eliminating canonical amino acid-specific *Mb*PylRS mutants from the library. After the final positive selection, 192 clones were screened at various concentrations of chloramphenicol in the presence and absence of Bpa (Supplementary Fig. 7). Two clones (viz. A2 and E10) survived at concentrations of chloramphenicol up to 300 µg ml^−1^ in the presence of Bpa, where no growth was observed on chloramphenicol concentration at and above 100 μg ml^−1^ in the absence of Bpa (Fig. 3b), indicating that these two clones specifically incorporate Bpa into the chloramphenicol acetyltransferase in response to a TAG stop codon. Both these clones were sequenced and found to be the same with the following mutations compared with the wild-type *Mb*PylRS, N311Q, C313T and W382A, in addition to the Y349F preprogrammed mutation, whereas position 386 remained unchanged. We named this mutant aaRS, *Mb*Pyl(Bpa)RS.

Next, we assessed the specificity and efficiency of the *Mb*Pyl(Bpa)RS by expressing a fusion protein of glutathione-*S*-transferase and calmodulin (Gst-CaM), where the first amino acid in calmodulin is incorporated in response to a TAG stop codon (*gst*-1TAG-*cam*) (Methods). ncAA incorporation efficiency was estimated by measuring the relative amount of full-length Gst-CaM to glutathione-*S*-transferase (Gst). As a control, we also performed a similar expression experiment with wt*-Mb*PylRS in the presence and absence of *N*6-(*tert*-butoxycarbonyl)-ʟ-lysine (BocK). Protein purification was performed using glutathione sepharose beads to isolate Gst-tagged proteins (Fig. 3c). The efficiency of *Mb*Pyl(Bpa)RS and wt*-Mb*PylRS at incorporating Bpa and BocK was estimated to be 49 and 40%, respectively (Supplementary Fig. 8). It may be noted that ncAA incorporation efficiency is not an absolute number and can vary widely with different plasmid systems^16,27^. It is the comparison between *Mb*Pyl(Bpa)RS and wt*-Mb*PylRS that is more important, with the latter known to efficiently incorporate BocK and often used as a benchmark^24^. These results demonstrate that the newly evolved *Mb*Pyl(Bpa)RS/*Mb*PyltRNA_CUA_ pair is highly efficient at site-specific incorporation of Bpa into proteins and was also found to be more efficient than the previously known *Mm*PylRS mutant, BpaRS1^35^ (Supplementary Fig. 8). Furthermore, expression of Gst-CaM using the *Mb*Pyl(Bpa)RS/*Mb*PyltRNA_CUA_ pair in the presence of *p*-Azido-ʟ-phenylalanine (AzF), BocK and pcY demonstrated that *Mb*Pyl(Bpa)RS does not incorporate AzF or BocK and is fivefold more specific for Bpa compared with pcY (Supplementary Fig. 8).

Subsequently, we assessed the specificity of the newly evolved *Mb*Pyl(Bpa)RS at incorporating Bpa into 7D12 in the presence of pcY. Single plasmids containing all three genetic elements (1) *7D12-32TAG* or *7D12-109TAG* gene, (2) *Mb*PyltRNA_CUA_ gene and (3) *Mb*Pyl(Bpa)RS gene were constructed (Methods). We named these plasmids as pSANG_7D12-32TAG_*Mb*Pyl(Bpa)_tRNA_CUA_ and pSANG_7D12-109TAG_*Mb*Pyl(Bpa)_tRNA_CUA_. Using these plasmids, protein expression was performed without ncAA, with Bpa, with pcY or with equimolar amounts of Bpa and pcY (Fig. 3d). Although in the absence of Bpa, *Mb*Pyl(Bpa)RS catalyzes some incorporation of pcY, MS results confirm that Bpa outcompetes pcY when the expression of 7D12 was performed in the presence of equimolar amounts of Bpa and pcY (Fig. 3e and Supplementary Fig. 9). Using *Mb*Pyl(Bpa)RS, we did not observe molecular weight corresponding to incorporation of pcY at either of the two positions, 32 or 109, in 7D12 when protein expression was performed in the presence of Bpa and pcY.

Thus, we evolved a highly efficient and specific *Mb*Pyl(Bpa)RS/tRNA pair that exclusively incorporates Bpa in the presence of pcY and could be employed for dual incorporation of Bpa and pcY in 7D12.

### Development of a photoactive, photoreactive 7D12 mutant

Two distinct ncAAs have been site-specifically incorporated into proteins expressed in *E. coli* by assigning (1) two distinct stop codons to ncAAs^36^, (2) a stop codon and a quadruplet codon to ncAAs^31^ or (3) two distinct quadruplet codons to ncAAs^37^. Of these, the expression system that utilizes a stop and a quadruplet codon, a quadruplet decoding evolved orthogonal ribosome and a quadruplet decoding evolved *Mb*PyltRNAs has demonstrated good protein yields^31,32^. We decided to employ this system for dual incorporation of pcY and Bpa in 7D12.

**Fig. 4:**
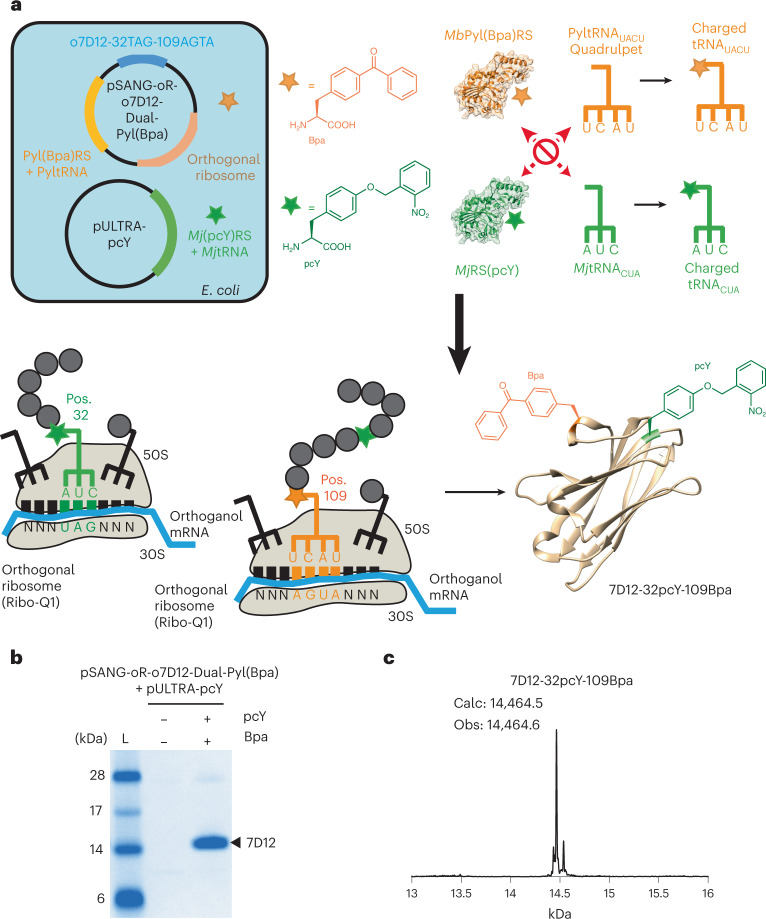
Site-specific dual incorporation of pcY and Bpa in 7D12. **a**, A single plasmid containing genes to assemble the orthogonal ribosome, our newly evolved Bpa-specific *Mb*Pyl(Bpa)RS, AGTA-decoding evolved *Mb*PyltRNA_UACU_, and *7D12* on orthogonal RBS was constructed. We named this plasmid pSANG-oR-o7D12-Dual-Pyl(Bpa). Cotransformation of this plasmid with pULTRA-pcY allowed for expression of 7D12-32pcY-109Bpa. mRNA, messenger RNA; Pos., position. **b**, Expression of 7D12-32pcY-109Bpa. Full-length 7D12 observed when expression was performed with both pcY (1 mM) and Bpa (1 mM; +pcY/+Bpa lane). See Extended Data Fig. 7 (some full-length protein also observed for expression performed with only pcY (+pcY/−Bpa lane) that might be due to incorporation of pcY by *Mb*Pyl(Bpa)RS in the absence of Bpa). These experiments were repeated twice with similar results. The lane marked L is the Invitrogen SeeBlue Plus2 Pre-stained Protein Standard (catalog no. LC5925). **c**, ESI-MS of 7D12-32pcY-109Bpa is consistent with site-specific incorporation of pcY and Bpa in 7D12. See Supplementary Fig. 11 for MS data before deconvolution. The ESI-MS data also show a minor peak at 14,537 Da, which is a mass gain of 72 Da on 7D12-32pcY-109Bpa. This peak cannot be explained by dual incorporation of pcY or Bpa, and we are unsure of its origin. Source data

First, we developed a plasmid for expressing wild-type *7D12* using the orthogonal ribosome. The ribosome binding site (RBS) of *7D12* was changed to an orthogonal RBS, forming the pSANG-o7D12 plasmid (Methods). Subsequently, a ribosomal RNA (rRNA) operon containing an evolved orthogonal 16S rRNA gene, as well as 23S and 5S rRNA genes, was cloned into the pSANG-o7D12 plasmid (Methods), forming pSANG-oR-o7D12; 0.7 mg of wt-7D12 per liter of culture was obtained using the pSANG-oR-o7D12 plasmid (Supplementary Fig. 10). Next, we mutated positions 32 and 109 in *7D12* to TAG and AGTA codons, respectively, forming pSANG-oR-o7D12-Dual plasmid (Methods). Genes for our newly evolved Bpa-specific *Mb*Pyl(Bpa)RS and an AGTA-decoding evolved *Mb*PyltRNA_UACU_ were then transplanted into the pSANG-oR-o7D12-Dual plasmid, creating a single plasmid that contains genetic components to assemble an orthogonal ribosome for the expression of *7D12* and allows for the encoding of Bpa in response to an AGTA codon (Methods). We named this plasmid pSANG-oR-o7D12-Dual-Pyl(Bpa) (Fig. 4a). pSANG-oR-o7D12-Dual-Pyl(Bpa) plasmid was cotransformed with pULTRA-pcY plasmid into BL21 cells for dual site-specific incorporation of pcY and Bpa in 7D12 (Fig. 4a).

**Extended Data Fig. 7:**
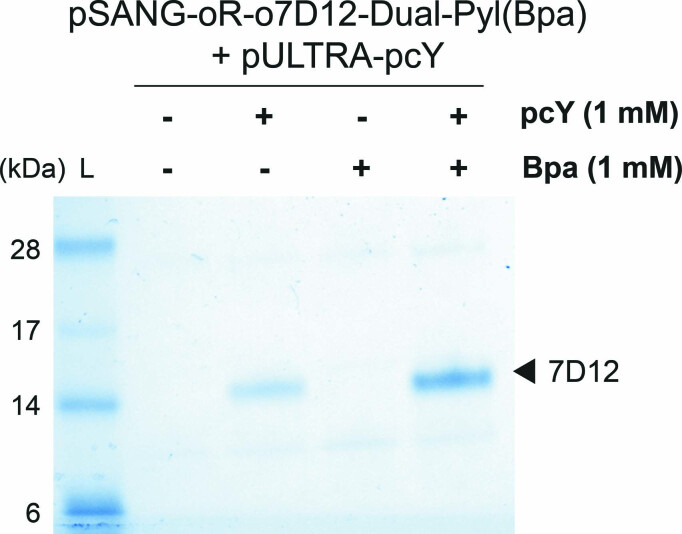
Expression of 7D12 using cells containing pSANG-oR-o7D12-Dual-Pyl(Bpa) and pULTRA-pcY plasmids performed without non-canonical amino acids, with 1 mM pcY, with 1 mM Bpa, and with 1 mM each of pcY and Bpa. Full-length protein observed for expression performed with only pcY (+pcY/ -Bpa lane) might be due to incorporation of pcY by *Mb*Pyl(Bpa)RS in the absence of Bpa. Comparison of band intensities for expression with only pcY (+pcY/ -Bpa lane), and with both pcY and Bpa (+pcY/ +Bpa lane) demonstrates that undesirable incorporation of pcY by *Mb*Pyl(Bpa)RS is an inefficient process and observed only in the absence of Bpa as confirmed by mass spectrometry data shown is Fig. 4c. This is consistent with data shown in Fig. 3c, Fig. 3d, and Fig. 3e. Lane marked L is the Invitrogen SeeBlue Plus2 Pre-stained Protein Standard (Catalog no. LC5925). These experiments were repeated twice with similar results. Source data

Protein expression was performed without the addition of any ncAA, with pcY, with Bpa, and with pcY and Bpa (Fig. 4b and Extended Data Fig. 7). Full-length 7D12 was observed when the expression was performed with both pcY and Bpa. In addition, some full-length protein was also obtained for expression with only pcY. This might be due to the undesired incorporation of pcY by *Mb*Pyl(Bpa)RS in the absence of Bpa, which is consistent with the data shown and discussed in Fig. 3c–e. MS results for expression product obtained in the presence of both pcY and Bpa prove dual incorporation of pcY and Bpa into 7D12 (Fig. 4c and Supplementary Fig. 11). Taken together, these results demonstrate site-specific incorporation of pcY at position 32 and Bpa at position 109 in 7D12, forming 7D12-32pcY-109Bpa.

**Fig. 5:**
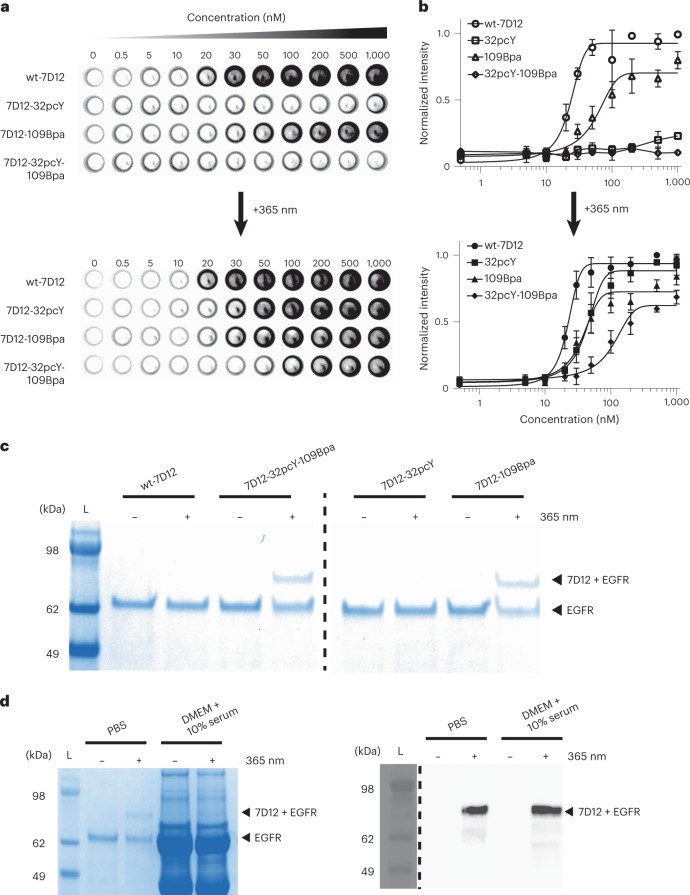
Development of photoactive-photoreactive 7D12 mutant. **a**, The on-cell binding assay demonstrates that 7D12-32pcY-109Bpa is a photoactive antibody. These experiments were performed in triplicate (Extended Data Fig. 8). **b**, The normalized intensities from the on-cell binding assay were plotted against the concentration of 7D12, where the *x* axis is in log_10_ scale. Each point in the graph represents mean values of normalized intensities ± s.d., designated as error bars, from three replicates. The data were fitted to the sigmoidal nonlinear equation using GraphPad to obtain binding affinity values (*K*_d_). Before irradiation, the *K*_d_ values of wt-7D12 and 7D12-109Bpa were 23 (±2.6) nM and 54 (±14) nM, respectively. After irradiation, the *K*_d_ values of wt-7D12, 7D12-32Bpa, 7D12-109Bpa and 7D12-32pcY-109Bpa were 22 (±1.5) nM, 42 (±5.4) nM, 35 (±6) nM and 103 (±25) nM, respectively (Extended Data Fig. 8). For 7D12-32Bpa and 7D12-32pcY-109Bpa before irradiation, lines show connection between individual points. For all other experiments, lines show the fitting trace. **c**, Photocrosslinked product observed only with 7D12-109Bpa and 7D12-32pcY-109Bpa for samples irradiated with 365-nm light, demonstrating that 7D12-32pcY-109Bpa gets activated and then forms a covalent bond with EGFR upon irradiation with 365-nm light (Extended Data Fig. 9). These experiments were repeated twice with similar results. **d**, Photocrosslinking of 7D12-32pcY-109Bpa to sEGFR performed in DMEM containing 10% serum. The left panel shows Coomassie-stained gel demonstrating photocrosslinking of 7D12-32pcY-109Bpa to sEGFR in the control reaction performed in PBS. For the same reaction performed in serum-containing media, bands corresponding to sEGFR and photocrosslinked product are not clear on Coomassie-stained gel due to the presence of serum proteins (Extended Data Fig. 10). The right panel shows the anti-His_6_ western blot of the photocrosslinking reactions that detects the C-terminal His_6_ tag on 7D12 (Extended Data Fig. 10). The bands show the sEGFR–7D12 complex demonstrating successful photocrosslinking of 7D12-32pcY-109Bpa in serum-containing media. These experiments were repeated twice with similar results. For gel images, lanes marked L are the Invitrogen SeeBlue Plus2 Pre-stained Protein Standard (catalog no. LC5925). Source data

**Extended Data Fig. 8:**
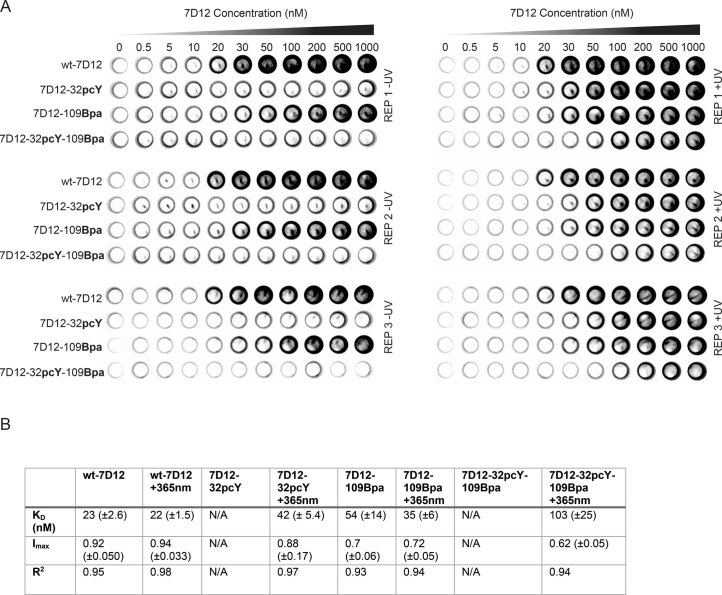
On-cell binding assay to measure the binding affinity of wt-7D12, 7D12-32pcY, 7D12-109Bpa, and 7D12-32pcY-109Bpa to EGFR expressed on A431 cells before and after irradiation with 365 nm light. (A) These results demonstrate 7D12-32pcY-109Bpa does not bind to EGFR and the binding is restored after irradiation with 365 nm light. To ensure reproducibility, experiments were performed in triplicates represented as REP 1, REP 2 and REP 3. (B) Quantitative assessment of binding between 7D12 mutants, viz. wt-7D12, 7D12-32pcY, 7D12-109Bpa, or 7D12-32pcY-109Bpa and EGFR. Chemiluminescence intensities obtained from on-cell binding experiments shown in ‘(A)’ were quantified using CLARIOstar plate reader. For each plate the maximum value of intensity was normalized to 1 and the minimum normalized to zero. Normalization is performed to ensure that data between replicates could be compared. The normalized intensity was plotted against concentration of 7D12. The data was fitted to sigmoidal nonlinear equation using GraphPad as shown in Fig. 5b. The K_D_ is the concentration of the 7D12, where chemiluminescence intensity is half of the maximum chemiluminescence intensity. Normalized I_MAX_ is the maximum chemiluminescence intensity at saturation. Values shown in brackets denote the standard deviation (s.d.) from the mean value of three replicates. Source data

Next, we measured the binding affinity of 7D12-32pcY-109Bpa to EGFR before and after 365-nm irradiation using our on-cell assay (Methods). As a control, we also measured the binding affinity of wt-7D12, 7D12-32pcY and 7D12-109Bpa. Without irradiation, near-background binding was observed for 7D12-32pcY-109Bpa and 7D12-32pcY. This is due to pcY at position 32 that inhibits 7D12–EGFR binding, consistent with our previous observations^16^ (Fig. 5a and Extended Data Fig. 8). The binding of 7D12-32pcY-109Bpa to EGFR was restored when the sample was irradiated with 365-nm light for 10 min. Also, as demonstrated in the previous section, Bpa at position 109 does not inhibit binding. The binding affinity of 7D12-32pcY-109Bpa to EGFR after irradiation was estimated to be 103 (±25) nM (Fig. 5b and Extended Data Fig. 8). These results demonstrate that 7D12-32pcY-109Bpa is a photoactive antibody.

We also demonstrate that both 7D12-32pcY-109Bpa and 7D12-109Bpa show near-background binding to MDA-MB-231 and SW620 cells when compared with A431 cells before or after irradiation with 365-nm light (Supplementary Fig. 12). The MDA-MB-231 cell line expresses much lower levels of EGFR than A431 cells, and SW620 cells have been previously used as the EGFR-negative cell line^16,38,39^. These results further demonstrate that the binding between 7D12 mutants containing site-specifically incorporated Bpa and pcY remains specific to EGFR-expressing cells before and after irradiation with 365-nm light.

**Extended Data Fig. 9:**
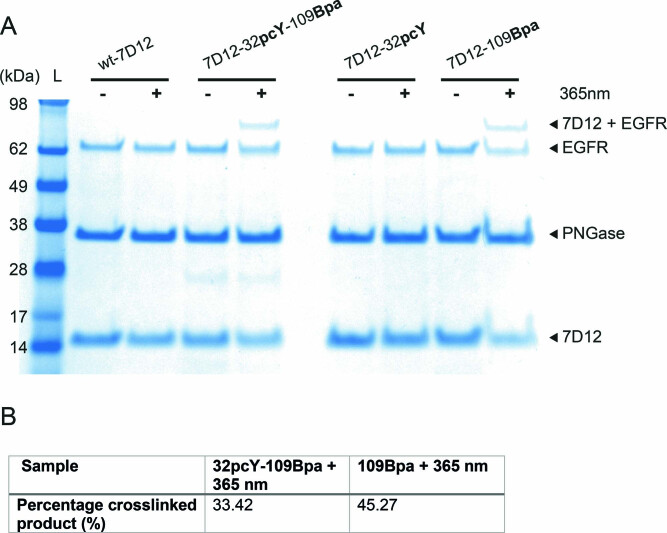
7D12-32pcY-109Bpa forms a covalent bond with EGFR upon irradiation with 365 nm light. (A) In the Coomassie stained gel image, the band higher than EGFR is photocrosslinked 7D12 + EGFR complex. This is the full gel image for data shown in Fig. 5c. Lane marked L is the Invitrogen SeeBlue Plus2 Pre-stained Protein Standard (Catalog no. LC5925). These results demonstrate that 7D12-32pcY-109Bpa and 7D12-109Bpa form covalent bond with EGFR upon irradiation with 365 nm light. No crosslinked product was observed for wt-7D12 or 7D12-32pcY. These experiments were repeated twice with similar results. (B) Percentage crosslinked product calculated by measuring the intensity of the band corresponding to 7D12 + EGFR and EGFR. Source data

**Extended Data Fig. 10:**
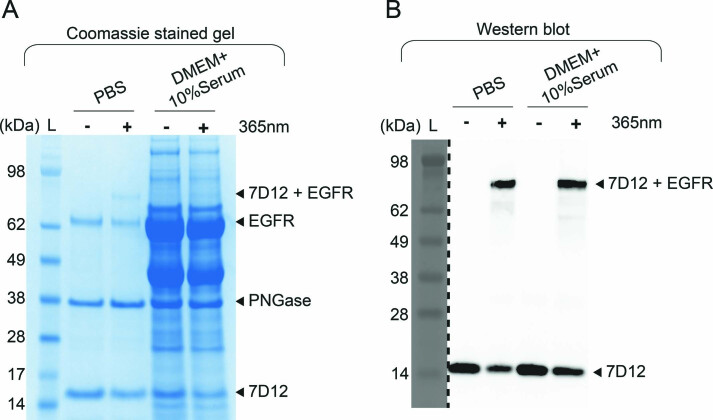
Photocrosslinking of 7D12-32pcY-109Bpa to EGFR performed in DMEM media containing 10% serum. (A) The Coomassie stained gel demonstrates successful photocrosslinking of 7D12-32pcY-109Bpa to EGFR in phosphate buffered saline (PBS). For the same reaction performed in serum containing media, bands corresponding to sEGFR and photocrosslinked product are not clear on Coomassie stained gel due to the presence of other proteins in the serum. This is the full gel image for data shown in Fig. 5d. Lane marked L is the Invitrogen SeeBlue Plus2 Pre-stained Protein Standard (Catalog no. LC5925). These experiments were repeated twice with similar results. (B) The anti-His_6_ antibody western blot detects the C-terminal His_6_ tag on 7D12-32pcY-109Bpa. The photocrosslinked product, 7D12+ EGFR (top band in the blot) appears in lanes where 7D12-32pcY-109Bpa/ sEGFR in PBS or in serum containing media are irradiated with 365 nm light, demonstrating successful photocrosslinking under both conditions. The lower band corresponds to 7D12-32pcY-109Bpa/ 7D12-109Bpa and serves as a control demonstrating detection 7D12-32pcY-109Bpa/ 7D12-109Bpa in all samples. The image was acquired using GE ImageQuant™ LAS 4000 gel imager. This is the full gel image for data shown in Fig. 5d. Lane marked L is the Invitrogen SeeBlue Plus2 Pre-stained Protein Standard (Catalog no. LC5925). These experiments were repeated twice with similar results. Source data

Next, we assessed the photoreactive property of 7D12-32pcY-109Bpa. In vitro photocrosslinking experiments were performed with 7D12-32pcY-109Bpa and as a control, with wt-7D12, 7D12-32pcY and 7D12-109Bpa (Fig. 5c and Extended Data Fig. 9). A band corresponding to the crosslinked 7D12–EGFR complex was observed only for samples containing 7D12-109Bpa and 7D12-32pcY-109Bpa after 365-nm irradiation. Thirty-three percent of 7D12-32pcY-109Bpa was estimated to crosslink to EGFR (Extended Data Fig. 9). Additionally, photocrosslinking of 7D12-32pcY-109Bpa was assessed in serum-containing media (Methods). Comparison of a Coomassie-stained gel and an anti-His_6_ western blot for detecting crosslinked product demonstrates successful light-mediated crosslinking of 7D12-32pcY-109Bpa to sEGFR in serum-containing media (Fig. 5d and Extended Data Fig. 10). These results demonstrate that 7D12-32pcY-109Bpa is a photoreactive antibody. Taken together, results from both the binding and the photocrosslinking assays demonstrate that 7D12-32pcY-109Bpa is a photoactive-photoreactive antibody fragment.

## Discussion

Genetically encoded photocaged ncAAs have been previously employed to obtain spatial and temporal control over the activity of several proteins^23,40^, and photoreactive ncAAs have been used to introduce light-dependent covalent ligation between interacting proteins^23,41^. However, proteins that can be simultaneously activated for binding and covalent ligation with their interaction partner upon photostimulation remain unknown. Here, we have developed a photoactive-photoreactive antibody fragment by site-specific incorporation of pcY and Bpa into an antibody fragment, 7D12, and demonstrate light-dependent activation of 7D12 binding and its covalent ligation to the interaction partner, EGFR.

Previous examples of site-specific installation of photocrosslinkers into antigen binding regions of the antibody fragments have led to a 10- to 100-fold decrease in their binding affinity^12,28^. We address this limitation in the present study by identifying position 109 in 7D12, where incorporation of the photocrosslinker, Bpa, has minimal effect on 7D12–EGFR binding affinity. The subsequent development of the photoactive, photoreactive 7D12 mutant required site-specific incorporation of two ncAAs (viz., pcY and Bpa). To address this challenge, we evolved a highly efficient and selective *Mb*PylRS mutant for incorporation of Bpa. This *Mb*PylRS mutant was shown to not incorporate a lysine derivative, BocK, or phenylalanine derivative, AzF, and thus, it could be used for incorporation of Bpa in the presence of these other ncAAs, aiding in further expansion of the genetic code of *E. coli*. *Mb*PylRS has also been used for ncAA incorporation in eukaryotic cells and animals; thus, the *Mb*PylRS(Bpa) mutant developed here could be used for efficient incorporation of Bpa in these systems.

We also developed a plasmid for efficient expression of 7D12 containing site-specifically incorporated pcY and Bpa. This plasmid system could be used to install other pairs of ncAAs in 7D12 and other antibody fragments, thus expanding the chemical space of this class of therapeutic and diagnostic proteins.

The photoactive-photoreactive antibody mutant developed here could address the challenges of toxicity and low residence time of its wild-type counterpart. These antibody fragments can be activated and covalently ligated to specific cancer cells close to the skin surface using wearable 365-nm UV-emitting LEDs, similar to the ones currently under investigation for treatment of vitamin D deficiency^42^. It has been demonstrated that UV light at 350-nm wavelength has a penetration depth of 60 μm for a person with white skin^43^. The depth of penetration can, however, vary with the site of the skin and can be over 160 μm for UV light at 340-nm wavelength^44^. It has also been demonstrated that UV radiations can penetrate through murine skin to activate intradermally injected light-sensitive therapeutics^45^. The drug, psoralen, that is activated by UV radiations is used for treatment of skin cancer^46^. Also, preclinical trials have been performed for the treatment of ovarian cancer in mouse models using UV light-activated antibody-based drugs^47^. Furthermore, surgically implanted biocompatible LEDs that can deliver greater than 100 mW per 1 cm^2^ of 360-nm radiation could also be used for activating such antibody fragments for treatment of solid tumors in other parts of the body^19,48^. It may be noted that long-wavelength UV radiations of 365 nm used for activation of photoactive-photoreactive antibodies in this study are known to cause less direct DNA damage and toxicity when compared with short-wavelength UV radiations^49^ and are thus considered much safer for clinical use.

The aaRS/tRNA pairs, plasmids and the methodology developed in this study would be extendable to the development of photoactive-photoreactive antibody fragments for other cellular targets. The increasing availability of high-resolution protein structures, the availability of advanced structure prediction methods^50^ and methods to predict the dynamics of protein containing ncAAs^16^ would greatly facilitate the design of new photoactive-photoreactive proteins. In addition to the translational potential of this work, the study provides (1) tools for efficient site-specific incorporation of Bpa using *Mb*PylRS(Bpa) in both prokaryotes and eukaryotes and (2) a generalized method for site-specific encoding of pcY and Bpa into proteins expressed in live cells. The latter could allow simultaneous activation of biological pathways by site-specifically incorporated pcY and identification of transient protein–protein interactions by site-specifically incorporated Bpa in vivo.

## Methods

### Cell lines, noncanonical amino acids and general methods

Human epithelial squamous carcinoma cell line, A431 (catalog no. 85090402), human breast adenocarcinoma cell line, MDA-MB-231 (catalog no. 92020424) and human colon adenocarcinoma cell line, SW620 (catalog no. 87051203) were purchased from Sigma-Aldrich, now Merck. All human cell lines were cultured in DMEM (Gibco; Life Technologies) containing ʟ-glutamine, 4.5 g l^−1^ d-glucose, 110 mg l^−1^ sodium pyruvate supplemented with 10% (vol/vol) FBS (Gibco; Life Technologies), and a cocktail of penicillin and streptomycin (Sigma-Aldrich). This medium will be referred to as ‘complete medium’. Cells were grown in 5% CO_2_ at 37 °C. Bpa (catalog no. 242935) was purchased from Fluorochem, and AzF (catalog no. F-3075.0005) and BocK (catalog no. E-1610.0005) were purchased from BACHEM. *O*-(2-Nitrobenzyl)-ʟ-tyrosine (pcY) was synthesized using procedures similar to those reported earlier^33^.

### General data collection and data analysis

SDS–PAGE gels and on-cell assay data was collected using BioRad Gel Doc XR+ gel imager, BioRad ChemiDoc XRS+ gel imager and GE ImageQuant LAS 4000 gel imager. On-cell assay quantitative data were collected using the CLARIOstar plate reader (BMG labtech). MS data were collected using a Bruker microQTOF-QIII mass spectrometer. LC–MS/MS measurements were performed on an Orbitrap Eclipse TribridTM mass spectrometer (Thermo Fisher Scientific) equipped with an UltiMate 3000 RSLCnano System (Thermo Fisher Scientific) using a nanoEase M/Z column (HSS C18 T3, 100 Å, 1.8 μm; Waters). For SDS–PAGE analysis, we used Image Lab (v.6.1.0, build 7). For DNA sequence analysis, we used Staden 2.0.0b11-2016 and SnapGene. For graph generation, we used Microsoft Excel (v.2210, build 16.0.15726.20188) and Graphpad Prism 9. MS/MS data analysis was performed using Scaffold v.5.1.2 (Proteome Software Inc.). Protein structure images were produced using UCSF chimera 1.16. Routine liquid chromatography–MS analysis was carried out as previously described^51^.

### Construction of pULTRA_*Mj*RS(Bpa)/*Mj*tRNA_CUA_

pULTRA-CNF plasmid (Addgene plasmid 48215) was digested with *Not*I, and the reaction mixture was run on a 1% agarose gel. The band corresponding to the pULTRA backbone was cut out and extracted using the QIAquick Gel Extraction Kit (QIAGEN). The gene for *Mj*RS(Bpa) (Supplementary Fig. 1) was amplified using polymerase chain reaction (PCR) from pSUP*Mj*RS(Bpa)/tRNA_CUA_ plasmid^31^ and subsequently cloned into the pULTRA backbone using Gibson cloning (New England Biolabs). The identity of pULTRA_*Mj*RS(Bpa)/*Mj*tRNA_CUA_ plasmid was confirmed by Sanger sequencing. This plasmid is referred to as pULTRA-Bpa in the text.

### Expression and purification of amber mutants of 7D12 with site-specifically incorporated Bpa

Chemically competent BL21(DE3)pLysS cells containing pULTRA-Bpa were transformed with pSANG10_7D12, pSANG10_7D12-32TAG, pSANG10_7D12-109TAG or pSANG10_7D12-113TAG plasmids. After transformation, cells were recovered in 1 ml of super optimal broth (SOB medium) for 1 h at 37 °C; 50 μl of recovered cells were transferred onto LB agar plates supplemented with 50 μg ml^−1^ kanamycin and 100 μg ml^−1^ spectinomycin. The plates were incubated overnight (37 °C, 16 h). A single colony from each plate was used to inoculate 50 ml of 2xTY-GKS media (2xTY media with 4% glucose, 50 μg ml^−1^ kanamycin and 100 μg ml^−1^ spectinomycin) and incubated overnight (37 °C, 220 r.p.m., 16 h). For large-scale expression and purification of 7D12 mutants, the next day this culture was used to inoculate fresh 500 ml 2xTY-GKS media so that its optical density measured at a wavelength of 600 nm (OD_600_) was 0.1. This was then incubated until OD_600_ reached 0.4−0.6 (37 °C, 220 r.p.m., 2–3 h), at which point IPTG (1 mM final concentration) and Bpa (1 mM final concentration) were added to induce the expression of *7D12* mutants. The cultures were incubated overnight (30 °C, 160 r.p.m., 16 h). The following day, cells were pelleted (3,200*g*, 4 °C, 10 min), the supernatant was discarded and the cells were resuspended in 25 ml periplasmic extraction buffer 1 (20% sucrose, 100 mM Tris-HCl, 1 mM EDTA, pH 8.0). The resuspended cells were incubated on ice for 30 min and then centrifuged (10,000*g*, 4 °C, 10 min). The supernatant was removed and stored at 4 °C (periplasmic fraction-1). The resulting pellet was resuspended in 25 ml periplasmic extraction buffer 2 (5 mM MgCl_2_) and incubated on ice for 20 min. The samples were centrifuged (10,000*g*, 4 °C, 10 min), and the supernatant was collected (periplasmic fraction-2). Both the periplasmic fractions were combined and passed through a 0.2-μm filter; 500 μl of Ni-NTA resin (ThermoFisher Scientific) was added to the periplasmic extract and mixed gently on a rocker (4 °C, 1 h). This was transferred into a gravity-flow column and washed three times with 10 ml PBS buffer each time. The resin was then washed twice with 8 ml Ni-NTA wash buffer (1× PBS supplemented with 20 mM imidazole). To elute the bound 7D12, 500 μl Ni-NTA elution buffer (1× PBS supplemented with 200 mM imidazole) was added and incubated for 15 min at room temperature, and the elution fraction was collected. This process was repeated eight times; the elution fractions were pooled together and dialyzed overnight at 4 °C against 1× PBS buffer. Dialyzed samples were then concentrated using a Vivaspin 500 column with a 3-kDa molecular mass cutoff (Sartorius), and the yields were determined using a colorimetric Pierce BCA protein assay (Thermo Fisher Scientific) measured at 562 nm. After protein purification and concentration, the samples were subsequently resolved by SDS–PAGE. To this end, Nu-PAGE LDS loading buffer (Invitrogen) and dithiothreitol (DTT) (at 100 mM final concentration) were added to 20 μl of protein samples, heated at 95 °C for 15 min, centrifuged (13,000*g*, 15 min, 4 °C) and loaded on a 4–12% Bis-Tris gel (Invitrogen) along with SeeBlue Plus2 protein ladder (Invitrogen). The gel was then stained with Coomassie Blue (InstantBlue; Abcam), and the identity of the protein was further confirmed by ESI-MS coupled with liquid chromatography. The molecular masses determined through SDS–PAGE and MS were in good agreement with the expected molecular mass of 7D12 and its mutants with site-specifically incorporated Bpa (Fig. 1b,c). A similar method was employed for the expression and purification of 7D12 mutants containing pcY. Small-scale expression and purification of *7D12* mutants have been described previously^16^.

### On-cell assay for measuring the binding of His-tagged antibody fragments

Cells were grown in a T-75 flask (Thermo Fisher Scientific) in complete medium (DMEM, 10% FBS, 1% of 100× penicillin and streptomycin solution) using standard tissue culture procedures until 80–90% confluence. After washing the cells with 1× PBS and trypsinising, they were spun down (300*g*, 5 min) and subsequently resuspended in 10 ml fresh complete medium. The cell density was established with a hemocytometer, and cells were diluted to 200,000 cells per milliliter. Two hundred microliters of this suspension was dispensed into each well (40,000 cells per well) of a white 96-well plate (Corning, catalog no. 3917) and grown overnight. After 12–16 h, the medium was replaced with 200 μl of fresh complete medium supplemented with 7D12 or its mutants at the desired concentration. The plate was incubated for 5 min (37 °C, 5% CO_2_). If the binding affinity was measured after 365-nm irradiation, this plate was placed on a transilluminator (GelDocMega; BioSystematica) and irradiated with a photon flux and intensity of 33 mW per 1 cm^2^ and 14 mW, respectively, at 365 nm for 10 min. The photon flux and the intensity of 365-nm light from the UV transilluminator were measured using a laser power meter (FieldMate; Coherent) at the surface of the transilluminator where the samples were placed. After removing the medium, the cells were washed once with complete medium (150 μl) and then fixed by adding 150 μl of 3.7% formaldehyde solution in sterile Mili-Q water to each well and incubating them for 20 min at room temperature. After removing the formaldehyde solution, cells were washed three times (150 μl, 5 min, gentle rocking) with 1× PBS supplemented with 0.1% Tween-20 (PBST). The wash buffer was subsequently exchanged for 100 μl of blocking buffer (10% milk in PBST), and cells were incubated at room temperature for 1 h with gentle rocking. Following on from this, the blocking buffer was exchanged for 50 μl of fresh blocking buffer (1% milk in PBST) containing 1,000-fold diluted primary mouse anti-6x-His tag antibody (Invitrogen, catalog no. MA1-21315), and the plate was incubated at room temperature for 1 h. After incubation with the primary antibody, cells in each well were washed three times with PBST (150 μl, 5 min, gentle rocking). Subsequently, 50 μl of horseradish peroxidase (HRP)-linked secondary antibody solution containing anti-mouse HRP-linked antibody (Cell Signaling, catalog no. 7076S) at 1:1,500 dilution and 1% milk in PBST was applied to each well and incubated at room temperature for 1 h. Next, the cells were washed six times with PBST (150 μl, 5 min, gentle rocking); finally, 150 μl of SuperSignal chemiluminescent Substrate (Thermo Fisher Scientific) was added to each well, and the plate was imaged using the BioRad ChemiDoc XRS+ gel imager or the GE ImageQuant LAS 4000 gel imager. The chemiluminescence intensity in each well was further quantified using a CLARIOstar plate reader (BMG Labtech).

### In vitro experiments to assess light-dependent covalent bond formation between 7D12 mutants and sEGFR

Various volumes of 50 μM stock of 7D12 mutants were mixed with 1 μl of 10 μM sEGFR (PeproTech, catalog no. 100-15 R) and 1 μl of 10× PBS in a final volume of 10 μl. The reaction mixture was incubated for 5 min at 37 °C. Samples were aliquoted on to a coverslip (VWR, catalog no. 631-0153) and then irradiated with 365-nm light using a transilluminator (GelDocMega; BioSystematica) with a photon flux and intensity of 33 mW per 1 cm^2^ and 14 mW, respectively, at 365 nm for 10 min. The photon flux and intensity of 365-nm light from the UV transilluminator were measured using a laser power meter (FieldMate; Coherent) at the surface of the transilluminator where the samples were placed. sEGFR in the reaction mixture and the crosslinked product were then enzymatically deglycosylated using PNGase F (New England Biolabs) before analysis using SDS–PAGE. This was achieved by adding 1 μl of glycoprotein denaturing buffer (10×) to the above reaction mixture, followed by incubation at 95 °C for 10 min. Denatured samples were then transferred to ice and incubated for 5 min followed by centrifugation at 13,000*g* for 5 min at 4 °C. This was followed by the addition of 2 µl GlycoBuffer 2 (10×), 2 µl 10% NP-40, 5 µl H_2_O and 1 µl PNGase F and incubation at 37 °C for 1 h. After addition of Nu-PAGE LDS loading buffer (Invitrogen) and DTT (at 100 mM final concentration), the samples were run on a 4–12% Bis-Tris gel (Invitrogen) along with protein ladder (SeeBlue Plus2 Prestained standard from Invitrogen) as a marker. The gel was then stained with Coomassie Blue (InstantBlue; Abcam) and imaged using GelDoc (Bio-Rad).

### In vitro experiments to assess light-dependent covalent bond formation between 7D12 mutants and sEGFR in serum-containing media

Two microliters of 50 μM stock of 7D12 mutants was mixed with 2 μl of 5 μM sEGFR (PeproTech, catalog no. 100-15 R) and 5 μl of DMEM containing 20% (vol/vol) serum in a final volume of 10 μl. DMEM (Gibco; Invitrogen) contains ʟ-glutamine, 4.5 g l^−1^ d-glucose and 110 mg l^−1^ sodium pyruvate. Serum is FBS (Gibco; Life Technologies). The reaction mixture was incubated for 5 min at 37 °C. The photocrosslinking was performed using the same procedure as described in In vitro experiments to assess light-dependent covalent bond formation between 7D12 mutants and sEGFR. The reaction was analyzed by Coomassie staining or western blotting. For western blotting, the proteins from 4–12% Bis-Tris gel were transferred onto a nitrocellulose membrane (Invitrogen iBlot 2 Transfer Stacks, nitrocellulose) using an iBlot 2 Dry Blotting System (Thermo Fisher Scientific). After transferring the proteins onto the nitrocellulose membrane, the membrane was incubated with the blocking buffer (PBST) for 1 h at room temperature. Subsequently, the blocking buffer was removed, and the membrane was washed with PBST. The membrane was then incubated with the primary antibody (6x-His Tag mAb (HIS.H8) mouse (Invitrogen, catalog no. MA1-21315) at 1:2,000 dilution in 1% milk and PBST) overnight at 4 °C. Subsequently, the membrane was washed three times with PBST. The membrane was then incubated with the secondary antibody (anti-mouse IgG, HRP-linked antibody (Cell Signaling, catalog no. 7076S) at 1:3,000 dilution in 1% milk and PBST) for 1 h at room temperature. Subsequently, the membrane was washed three times with PBST. Finally, for signal development, the membrane was incubated with the substrate for horseradish peroxidase, SuperSignal West Pico PLUS Chemiluminescent Substrate (Thermo Fisher Scientific). The membrane was then imaged using a GE ImageQuant LAS 4000 gel imager.

### Effect of 365-nm irradiation on A431 cells assessed using the cell viability assay

A431 cells were grown in a T-75 flask in complete medium using standard tissue culture procedures until 80–90% confluence. Cells were then washed once with Dulbecco’s phosphate-buffered saline, detached using Trypsin-EDTA, pelleted (300*g*, 5 min) and resuspended in 10 ml fresh complete medium. The resuspended cells were then counted using a hemocytometer and diluted to 200,000 cells per milliliter of complete medium; 200 µl of these cells were seeded (40,000 cells per well) into each well of a white 96-well plate (Corning). The plates were incubated overnight, 12–16 h (37 °C, 5% CO_2_). The following day, medium in each well was replaced with fresh prewarmed complete medium. These plates were then irradiated with 365-nm light for 0, 5, 10 and 15 min using a transilluminator (GelDocMega; BioSystematica) at the photon flux and intensity of 33 mW per 1 cm^2^ and 14 mW, respectively, at 365 nm. After irradiation, medium in each well was removed and replaced with 90 µl fresh prewarmed complete medium and 10 µl alamarBlue reagent. This was incubated for 2 h (37 °C, 5% CO_2_). The fluorescence emission at 590 nm (with excitation at 560 nm) from each well was quantified using the CLARIOstar plate reader (BMG labtech). The fluorescence intensity of a standard containing no cells was subtracted from the fluorescence intensity from each well. Subsequently, these intensities were normalized by dividing the intensity values by the mean intensity value obtained from the no irradiation control experiment and plotted as a bar graph. Six replicates of each experiment were performed.

### MS analysis of photocrosslinked 7D12-109Bpa–sEGFR complex

The band corresponding to photocrosslinked 7D12–EGFR was excised from SDS–PAGE gels, destained, and digested with sequencing-grade trypsin/chymotrypsin, as previously described^52^. Aliquots of the peptides were used for LC–MS/MS analysis on an Orbitrap Eclipse Tribrid mass spectrometer (Thermo Fisher Scientific) equipped with an UltiMate 3000 RSLCnano System (Thermo Fisher Scientific) using a nanoEase M/Z column (HSS C18 T3, 100 Å, 1.8 μm; Waters). The samples were loaded and trapped using a precolumn with 0.1% TFA at 20 µl min^−1^ for 3 min. The trap column was then switched in line with the analytical column for separation using the following long gradient of solvents A (water, 0.05% formic acid) and B (80% acetonitrile, 0.05% formic acid) at a flow rate of 0.2 µl min^−1^: 0–4 min 3% B, 4–10 min linear increase B to 7%, 10–70 min increase B to 37% and 70–90 min increase B to 55% followed by a ramp to 99% B and reequilibration to 3% B, for a total running time of 122 min. The peak lists were used to search against a custom database containing the proteins of interest in a background of common contaminants (MaxQuant) using an in-house Mascot Server v.2.8.0 (Matrix Science) with trypsin/chymotrypsin digestion and two missed cleavages. Oxidation (Met), acetylation (protein N terminus) and deamidation (Asn, Gln) were used as variable modifications, and carbamidomethyl (Cys) was used as a fixed modification. Mass tolerances were 6 ppm for precursor ions and 0.6 Da for fragment ions. Scaffold v.5.1.2 (Proteome Software Inc.) was used to validate MS/MS-based peptide and protein identifications. Peptide identifications were accepted if they could be established at greater than 95.0% probability by the Peptide Prophet algorithm with Scaffold delta-mass correction^53^. Peptide identifications of ≥95.0% probability and protein identifications of ≥99.0% probability by the Peptide Prophet algorithm were accepted with at least one identified peptide in Scaffold 5.

### *M. barkeri* pyrrolysyl-tRNA synthetase library generation for directed evolution

We first constructed the Y349F mutant gene of PylRS. pBK_pylRS^54^ was PCR amplified using the primer pair Mb_pylRS_Y349F_F and Mb_pylRS_Y349F_R (Supplementary Table 1) and Q5-DNA polymerase (New England Biolabs (NEB)) according to the manufacturer’s instructions. The PCR product was subsequently purified using the PCR purification kit (QIAGEN). The purified PCR product was digested with DpnI and BsaI (NEB), ligated using T4 DNA ligase (NEB) and transformed into electrocompetent *E. coli* GeneHog cells (Thermo Fisher Scientific). Plasmid isolated from the respective transformant was checked for the presence of the desired mutation using Sanger sequencing. Hereafter, the mutation carrying the plasmid, pBK_pylRS_349F, was used as template for construction of the library. The first round of inverse PCR was performed to mutate positions 311 and 313 to NNK codons. This was achieved using the primer pair N311_C313_F and N311_C313_R (Supplementary Table 1) and amplification by Q5-DNA polymerase (NEB). The PCR product was subsequently purified using the PCR purification kit (QIAGEN). The purified PCR product was digested with DpnI and BsaI (NEB), ligated using T4 DNA ligase (NEB) and transformed into electrocompetent *E. coli* GeneHog cells. The electroporated cells were recovered in 1 ml SOC medium for 1 h at 37 °C. To this, 6 ml of LB medium supplemented with kanamycin (50 μg ml^−1^ in the final volume) was added, and a small aliquot (0.1 ml) was taken to generate a serial dilution series that was plated onto LB agar containing kanamycin to determine the number of clones in the library. The rest of the culture was incubated overnight (37 °C, 220 r.p.m., 12–16 h). The next morning, the cells were pelleted by centrifugation (3,200*g*, 4 °C, 10 min), and the plasmid DNA was isolated using the QIAGEN plasmid miniprep kit (QIAGEN). This plasmid DNA library was called pBK_pylRS_349F_311NNK_313NNK. Using the LB agar plates, we determined that the library contained a total of 1 × 10^6^ clones. The isolated plasmid DNA was used as a template for the next round of inverse PCR to mutate positions 382 and 386 to NNK codons. The procedure of PCR, digestion and transformation was again repeated. Note that three independent 50-μl PCR reactions were performed using 4 ng of pBK_pylRS_349F_(311,313)X template DNA each, primer pair MbpylRS_W382_G386X_F and MbpylRS_W382_G386X_R (Supplementary Table 1), and Q5-DNA polymerase (NEB). To guarantee a sufficient number of transformants in the final library, a total of 12 electroporations were carried out, and after regenerating the cells for 1 h in 12 ml SOC medium at 37 °C, 38 ml of LB medium and kanamycin for a final concentration of 50 μg ml^−1^ were added, and the culture was incubated shaking overnight. The final library contained a total of 1.8 × 10^8^ clones and was assessed through Sanger sequencing of the *pylRS* gene to verify its quality. This plasmid DNA library was called pBK_pylRS_349F_(311,313,382,386)X.

### Directed evolution of *M. barkeri* pyrrolysyl-tRNA synthetase for efficient site-specific incorporation of Bpa into proteins

Freshly prepared *E. coli* GeneHog cells (Thermo Fisher Scientific) carrying the pREP-PylT plasmid^54^ were electroporated with the pBK_pylRS_349F_(311,313,382,386)X library DNA. The cells were recovered in SOC media containing 1 mM Bpa at 37 °C for 1 h and subsequently plated onto LB agar containing 25 μg ml^−1^ kanamycin, 12.5 μg ml^−1^ tetracycline, 50 μg ml^−1^ chloramphenicol and 1 mM Bpa. These plates were incubated at 37 °C for 36–48 h, and the resulting colonies were washed with LB medium. Plasmid DNA from the colonies was isolated using the QIAGEN plasmid miniprep kit (QIAGEN). The isolated plasmid DNA was digested with *DraI* (NEB) to remove the pREP plasmid, purified using SureClean (Bioline) and subsequently electroporated into *E. coli* GeneHog cells containing the pYOBB2 plasmid^54^ for the negative selection. The cells were recovered in SOC media containing 0.2% arabinose at 37 °C for 1 h and plated onto LB agar containing 50 μg ml^−1^ kanamycin, 50 μg ml^−1^ chloramphenicol and 0.2% arabinose. The plates were incubated at 37 °C for 14–18 h. The surviving colonies were washed with LB medium, and the plasmid DNA was isolated using the QIAGEN plasmid miniprep kit. The isolated plasmid DNA was digested with *DraI* (NEB) to remove the pYOBB2 plasmid, purified using SureClean and subsequently electroporated into *E. coli* GeneHog cells containing pREP plasmid for the final positive selection. A procedure similar to the first positive selection was followed; 192 colonies from the final positive selection plate were picked and transferred to two 96-deep well plates (Thermo Fisher Scientific), where each well contained 200 µl LB medium supplemented with 50 μg ml^−1^ kanamycin and 25 μg ml^−1^ tetracycline. The plates were incubated at 37 °C with shaking at 220 r.p.m. overnight (12–16 hours). The next morning, 1 μl of overnight culture from each well was used to inoculate two cultures, one without and the other with 1 mM Bpa (all cultures were supplemented with 25 μg ml^−1^ kanamycin and 12.5 μg ml^−1^ tetracycline) in four 96-deep well plates. The plates were incubated at 37 °C with shaking at 220 r.p.m. for 3 h, and subsequently, 2.5 µl from each well were spotted onto LB agar plates with and without 1 mM Bpa at a range of chloramphenicol concentrations (viz. 0, 100, 200 and 300 μg ml^−1^). All LB agar plates were supplemented with 25 μg ml^−1^ kanamycin and 12.5 μg ml^−1^ tetracycline. The plates were incubated at 37 °C for 12–16 h and imaged using GelDoc (Bio-Rad).

### Construction of pREP_gst-1TAG-cam plasmid

The *gst-cam* gene cassette encompassing the lac promoter and the *gst-cam* fusion gene was PCR amplified using Q5-DNA polymerase and primers gst-cam_pREP_F and gst-cam_pREP_R (Supplementary Table 1), with the pRSF_wtRibo_wtRBS-gst-1TAG_cam plasmid^31^ serving as template DNA. The resulting PCR product was cloned into pREP plasmid digested with BsaAI and ScaI (NEB) using a Gibson Assembly Cloning Kit (NEB) according to the manufacturer’s instructions. The sequence of the resulting plasmid pREP_gst-1TAG-cam was verified through Sanger sequencing.

### Expression and purification of Gst-CaM for assessing the specificity and efficiency of the *Mb*Pyl(Bpa)RS

*E. coli* GeneHog cells containing pREP_gst-1TAG-cam plasmid were transformed with pBK_*Mb*Pyl(Bpa)RS plasmid and plated onto LB agar containing 50 μg ml^−1^ kanamycin and 25 μg ml^−1^ tetracycline (LB-KT). The following day, a single colony was inoculated into 5 ml LB-KT media and incubated overnight (37 °C, 220 r.p.m., 14–16 h). The overnight culture was diluted to OD_600_ of 0.1 in fresh 25 ml LB-KT media and incubated at 37 °C and 220 r.p.m. until the OD_600_ reached 0.4–0.6. The cultures were induced with IPTG (1 mM, final concentration) split into two cultures of 10 ml each, and Bpa (1 mM, final concentration) was added to one of the cultures. The cultures were incubated overnight (14–16 h, 37 °C, 220 r.p.m.), pelleted the next day (5,000*g*, 10 min, 4 °C), washed with 1 ml PBS and suspended in 800 μl of BugBuster protein extraction reagent (Novagen) supplemented with protease inhibitor cocktail tablets (Roche), 1 mg ml^−1^ lysozyme and 1 mg ml^−1^ DNase I. The cell suspension was incubated at 25 °C for 1 h on a ThermoMixer (Eppendorf) at 900 r.p.m. Cell debris was pelleted (17,000*g*, 30 min, 4 °C), the supernatant was transferred to a fresh tube and 50 μl of Glutathione Sepharose 4b beads (Cytiva) were added. The beads were incubated with the extracted proteins for 1 h at 4 °C to bind Gst-tagged proteins. Subsequently, the beads were spun down at 500*g* at 4 °C and washed with 800 μl PBS (four times). Finally, the bound protein was eluted by heating the beads in 1× LDS sample buffer (Thermo Fisher Scientific) supplemented with 100 mM DTT at 95 °C for 5 min. The beads were spun down (17,000*g* for 10 min at 4 °C), and the supernatant was analyzed on a 4–12% Bis-Tris gel. In addition, SeeBlue Plus2 prestained protein standard (Invitrogen) was also loaded on the gel as marker. The gels were subsequently stained with InstantBlue (Abcam) Coomassie protein stain.

### Construction of pSANG_7D12-32TAG_*Mb*Pyl(Bpa)_tRNA_CUA_ and pSANG_7D12-109TAG_*Mb*Pyl(Bpa)_tRNA_CUA_ plasmids

First, the gene for *Mb*Pyl(Bpa)RS*-*A2 was cut out of pBK_ *Mb*Pyl(Bpa)RS*-*A2 plasmid with NdeI and PstI and subcloned into two other plasmids pAS61 and pAS64. pAS61 and pAS64 plasmids contain a single copy of genes for the wt-*Mb*PylRS/*Mb*PyltRNA_CUA_ pair and wt-*Mb*PylRS/ev*Mb*PyltRNA_UACU_ pair, respectively. The resulting pAS61 and AS64 derivatives were named pAS61-A2 and pAS64-A2, respectively. Next, the DNA fragments comprising genes for the *Mb*Pyl(Bpa)RS*-*A2/*Mb*PyltRNA_CUA_ pair from pAS61-A2 were PCR amplified using primers AS61_to_pSang_F and AS61_to_pSang_R (Supplementary Table 1) and Q5-DNA polymerase (NEB). This PCR product was cloned into pSANG10_7D12-32TAG and pSANG10_7D12-109TAG plasmids that had been previously digested with SphI using the Gibson Assembly Cloning Kit (NEB) according to the manufacturer’s instructions. The integrity of the resulting plasmids pSANG_7D12-32TAG_*Mb*Pyl(Bpa)_tRNA_CUA_ and pSANG_7D12-109TAG_*Mb*Pyl(Bpa)_tRNA_CUA_ was verified through Sanger sequencing.

### Construction of pSANG-oR-o7D12 plasmid

We first changed the RBS of *7D12* in pSANG10-wt7D12 plasmid^16^ to orthogonal RBS^31^. pSANG10-wt7D12 plasmid was digested using XbaI and SalI (NEB). After digestion, the reaction mixture was run on a 1% agarose gel. The band corresponding to the pSANG-10 backbone was extracted using the QIAquick Gel Extraction Kit (QIAGEN). A gene fragment containing orthogonal RBS and part of *7D12* (obtained from Integrated DNA Technologies) (Supplementary Fig. 13) was cloned into the digested pSANG-10 backbone using Gibson cloning (NEB). We named this plasmid as pSANG-o7D12. The integrity of pSANG-o7D12 plasmid was confirmed by Sanger sequencing. Next, the orthogonal 16S ribosomal RNA (o-16S rRNA), 23S rRNA and 5S rRNA genes were inserted into pSANG-o7D12. o-16S rRNA, 23S rRNA and 5S rRNA genes were amplified from pRSF-riboQ1-o-gst-cam plasmid^31^ using the primers orRNA_RSF_SANG_F and orRNA_RSF_SANG_R (Supplementary Table 1) and Q5-DNA polymerase (New England Biolabs) according to the manufacturer’s instructions. The PCR product was purified using the PCR purification kit (QIAGEN). For insertion of this PCR product into pSANG-o7D12, pSANG-o7D12 plasmid was digested with BglI (NEB). After digestion, the reaction mixture was run on a 1% agarose gel. The band corresponding to the linear pSANG-o7D12 plasmid was extracted using the QIAquick Gel Extraction Kit (QIAGEN). The PCR product containing o-16S rRNA, 23S rRNA and 5S rRNA genes was cloned into this linear plasmid using the Gibson Assembly Cloning Kit (NEB). We named this plasmid as pSANG-oR-o7D12. The integrity of pSANG-oR-o7D12 plasmid was confirmed by Sanger sequencing.

### Construction of pSANG-oR-o7D12-Dual (pSANG-oR-o7D12-32^TAG^109^AGTA^) plasmid

pSANG-oR-o7D12 plasmid was digested using SgrA1 and BlpI (NEB) to remove the o*7D12* DNA fragment from this plasmid. After digestion, the reaction mixture was run on a 1% agarose gel. The band corresponding to the pSANG-oR backbone was extracted using the QIAquick Gel Extraction Kit (QIAGEN). A gene fragment containing orthogonal RBS and *7D12*-32TAG-109AGTA (obtained from Integrated DNA Technologies) (Supplementary Fig. 14) was cloned into the digested pSANG-oR backbone using the Gibson Assembly Cloning Kit (NEB). We named this plasmid as pSANG-oR-o7D12-Dual. The integrity of pSANG-oR-o7D12-Dual plasmid was confirmed by Sanger sequencing the *7D12* region in the plasmid.

### Construction of pSANG-oR-o7D12-Dual-Pyl(Bpa) (pSANG-oR-o7D12-32^TAG^109^AGTA^-*Mb*Pyl(Bpa)-evtRNA_UACU_) plasmid

First, pSANG-oR-o7D12-Dual plasmid was digested with BamHI (NEB). After digestion, the linearized plasmid was purified using the QIAquick PCR Purification Kit (QIAGEN). Next, the DNA fragment containing the genes for the *Mb*Pyl(Bpa)RS*-*A2/ev*Mb*PyltRNA_UACU_ pair from pAS64-A2 (for pAS64-A2, see Construction of pSANG_7D12-32TAG_*Mb*Pyl(Bpa)_tRNA_CUA_ and pSANG_7D12-109TAG_*Mb*Pyl(Bpa)_tRNA_CUA_ plasmids) was PCR amplified using primers o-pSANG_AS61_BamHI_f and o-pSANG_AS61_BamHI_r (Supplementary Table 1) and Q5-DNA polymerase (NEB). This PCR product was cloned into pSANG-oR-o7D12-Dual plasmid previously digested with BamHI using the Gibson Assembly Cloning Kit (NEB) according to the manufacturer’s instructions. We named this plasmid as pSANG-oR-o7D12-Dual-Pyl(Bpa). The identity of pSANG-oR-o7D12-Dual-Pyl(Bpa) plasmid was confirmed by Sanger sequencing.

### Reporting summary

Further information on research design is available in the Nature Portfolio Reporting Summary linked to this article.

## Online content

Any methods, additional references, Nature Portfolio reporting summaries, source data, extended data, supplementary information, acknowledgements, peer review information; details of author contributions and competing interests; and statements of data and code availability are available at 10.1038/s41589-022-01251-9.

## Supplementary information

Supplementary Information Table of contents, Supplementary Figs. 1–14 and Table 1.

Reporting Summary

## Data Availability

All data supporting the finding reported in this manuscript are available in the main manuscript and supplementary information. Raw data are available as source data files. The crystal structure of the 7D12–EGFR complex was accessed via Protein Data Bank (PDB ID 4KRL). The crystal structure of PylRS with adenylated pyrrolysine was accessed via Protein Data Bank (PDB ID 2Q7H). All unique materials developed in this study can be obtained by writing to the corresponding author. The requesting person/organization will need to sign a materials transfer agreement with the University of East Anglia (UK) and might have to bear reasonable shipping costs. Source data are provided with this paper.

## Source data

Source Data Fig. 1 Full-length Coomassie-stained SDS–PAGE gel in Fig. 1.
Source Data Fig. 1 Raw mass spectrometry data in Fig. 1.
Source Data Fig. 2 Source data from on-cell assay in Fig. 2.
Source Data Fig. 3 Full-length Coomassie-stained SDS–PAGE gel in Fig. 3.
Source Data Fig. 3 Raw mass spectrometry data in Fig. 3.
Source Data Fig. 4 Full-length Coomassie-stained SDS–PAGE gel in Fig. 4.
Source Data Fig. 4 Raw mass spectrometry data in Fig. 4.
Source Data Fig. 5 Source data from on-cell assay in Fig. 5.
Source Data Extended Data Fig. 1 Source data from on-cell assay in Extended Data Fig. 1.
Source Data Extended Data Fig. 2 Coomassie-stained SDS–PAGE band intensity calculations for Extended Data Fig. 2.
Source Data Extended Data Fig. 3 Coomassie-stained SDS–PAGE band intensity calculations for Extended Data Fig. 3.
Source Data Extended Data Fig. 4 Coomassie-stained SDS–PAGE band intensity calculations for Extended Data Fig. 4.
Source Data Extended Data Fig. 5 Full-length Coomassie-stained SDS–PAGE gel in Extended Data Fig. 5.
Source Data Extended Data Fig. 6 Full-length SDS–PAGE gel and blots in Extended Data Fig. 6.
Source Data Extended Data Fig. 7 Full-length Coomassie-stained SDS–PAGE gel in Extended Data Fig. 7.
Source Data Extended Data Fig. 8 Source data from on-cell assay in Extended Data Fig. 8.
Source Data Extended Data Fig. 9 Coomassie-stained SDS–PAGE band intensity calculations for Extended Data Fig. 9.
Source Data Extended Data Fig. 10 Full-length SDS–PAGE gel and blots in Extended Data Fig. 10.
