# Supplementary material for: Site-specific encoding of photoactivity and photoreactivity into antibody fragments
Source: Nat Chem Biol. 2023 Feb 16;19(6):740–9. doi: 10.1038/s41589-022-01251-9 (PMC10229432; doi:10.1038/s41589-022-01251-9)
Supplement: Supplementary file 1 — Table of contents, Supplementary Figs. 1–14 and Table 1. [file 41589_2022_1251_MOESM1_ESM.pdf]

nature chemical biology

Article https://doi.org/10.1038/s41589-022-01251-9

Site-specific encoding of photoactivity and photoreactivity into antibody fragments

In the format provided by the authors and unedited

Table of Contents

Supplementary Figure 1: The sequence of *Mj*RS(Bpa) in pULTRA-Bpa plasmid.................. 4

Supplementary Figure 2: Raw mass spectrometry data before deconvolution for: A) wt-7D12, B) 7D12-32Bpa, C) 7D12-109Bpa, and D) 7D12-113Bpa. Mass spectra after deconvolution are shown in Figure 1c. 5

Supplementary Figure 3: Effect of 365 nm irradiation on the viability of A431 cells assessed using alamarBlue cell viability assay (see Methods). After adding the alamarBlue reagents, the fluorescence emission at 590 nm (with excitation at 560 nm) was quantified using CLARIOstar plate reader (BMG labtech). The fluorescence intensity of a standard containing no cells was subtracted from the fluorescence intensity from each well. Subsequently, these intensities were normalised by dividing the intensity values by mean intensity value obtained from no irradiation control experiment, and plotted as a bar graph. Six replicates of each experiment were performed. The normalised intensities from each of these replicates are shown as circles in the bar graph. The top of each bar in the bar graph represents mean values of normalised intensities ± s.d, designated as error bar, from six independent replicates. The results demonstrate that greater than 90% of the cells are viable upon 10 min irradiation with 365 nm light.. 6

Supplementary Figure 4. Liquid chromatography tandem mass spectrometry (MS/MS) analysis of the photocrosslinked 7D12-109Bpa-sEGFR complex demonstrates the presence of 7D12 in the crosslinked product. The 7D12 sequence is covered by 14 unique peptides (73% of the sequence) highlighted in grey. Location of 109Bpa (B) is highlighted in cyan. Representative MS/MS spectra of detected peptides matching the 7D12 sequence are also shown. Assigned b- and y-ions are annotated in red and blue, respectively. Ions belonging to other series, e.g. a-ions, are shown in green. Data was visualised using Scaffold 5 (see Methods for further details). 7

Supplementary Figure 5. Liquid chromatography tandem mass spectrometry (MS/MS) analysis of the photocrosslinked 7D12-109Bpa-sEGFR complex demonstrates presence of EGFR in the crosslinked product. Protein sequence of EGFR showing detected peptides (49 unique peptides, covering 62% of the sequence; highlighted in grey). Representative MS/MS spectra of detected peptides matching the EGFR sequence are also shown. Assigned b- and y-ions are annotated in red and blue, respectively. Ions belonging to other series, e.g. a-ions, are shown in green. Data was visualised using Scaffold 5 (see Methods for further details). 9

Supplementary Figure 6: Assessing the selectivity of *Mj*RS(pcY)/*Mj*tRNA$_{CUA}$, and *Mj*RS(Bpa)/*Mj*tRNA$_{CUA}$ at site-specific incorporation of pcY and Bpa at position 109 in 7D12. For *Mj*RS(pcY)/*Mj*tRNA$_{CUA}$, band corresponding to full-length 7D12 is only observed when the expression is performed in the presence of pcY, demonstrating that *Mj*RS(pcY) is specific for pcY. For *Mj*RS(Bpa)/*Mj*tRNA$_{CUA}$, band corresponding to full 7D12 is observed when the expression is performed in the presence of pcY or Bpa or without any non-canonical amino acid, demonstrating that *Mj*RS(Bpa) is promiscuous. These gel images are obtained after Coomassie staining. Lane marked L' is the Thermo Scientific PageRuler Unstained Low Range Protein Ladder (Catalog no. 26632). This experiment was repeated twice with similar results... 15

Supplementary Figure 7: Screening of *Mb*PylRS mutants obtained after three rounds of directed evolution to isolate novel *Mb*PylRS mutants for site-specific incorporation of Bpa.

192 colonies (or *Mb*PylRS mutants) were screened for efficient site-specific incorporation of Bpa at position 111 in chloramphenicol acetyl transferase (CAT). Two colonies, A2 on Plate I and E10 on Plate II (circled red) survived on chloramphenicol concentration up to 300 μg/ml in the presence of 1 mM Bpa, but for these colonies no growth was observed in the absence of Bpa on chloramphenicol concentration at and above 100 μg/ml. The results demonstrate that *Mb*PylRS mutants in A2 and E10 are likely to efficiently incorporate Bpa but no other naturally occurring canonical amino acid. 16

Supplementary Figure 8: Assessing the efficiency and substrate specificity of newly evolved *Mb*Pyl(Bpa)RS and previously known *Mm*Pyl(Bpa)RS, BpaRS1 (Chembiochem, 2013. 14(16): p. 2100-5), at site-specific incorporation of Bpa. Expression of *gst-1TAG-cam* gene is performed with *Mb*Pyl(Bpa)RS, and BpaRS1 either without any noncanonical amino acid (ncAA), or with p-benzoyl-L-phenylalanine (Bpa), O-(2-Nitrobenzyl)-L-tyrosine (photocaged tyrosine, pcY), p-Azido-L-phenylalanine (AzF) or N6-(tert-butoxycarbonyl)-L-lysine (BocK). As a control, expression of *gst-1TAG-cam* gene was also performed with wt-*Mb*PylRS that is known to efficiently incorporate BocK. Site-specific incorporation of amino acid in response to TAG stop codon will result in the full-length Gst-CaM (top band). A) Comparison of band intensities of full length Gst-CaM for expressions performed with Bpa (1mM), pcY(1mM), AzF(1mM) and BocK(1mM) using *Mm*Pyl(Bpa)RS and newly evolved *Mb*Pyl(Bpa)RS demonstrates that latter is highly efficient and specific at incorporating Bpa in Gst-CaM. This gel image is obtained after Coomassie staining. B) ncAA incorporation efficiency is calculated by taking the ratio of intensity of the top band (Gst-CaM) to the sum of intensities of top band (Gst-CaM) and lower band (Gst). The efficiency of *Mb*Pyl(Bpa)RS at incorporating Bpa and that of wt-*Mb*PylRS at incorporating BocK are similar. Also, *Mb*Pyl(Bpa)RS is 5-fold selective for Bpa over pcY. Lane marked L is the Invitrogen SeeBlue Plus2 Pre-stained Protein Standard (Catalog no. LC5925). These experiments were repeated twice with similar results. 17

Supplementary Figure 9: Raw mass spectrometry data before deconvolution for protein samples obtained after purification for expression of: A) 7D12-32TAG with *Mb*Pyl(Bpa)RS/ *Mb*PyltRNA$_{CUA}$ pair in the presence of 1 mM Bpa, B) 7D12-32TAG with *Mb*Pyl(Bpa)RS/ *Mb*PyltRNA$_{CUA}$ pair in the presence of 1 mM Bpa and 1 mM pcY, C) 7D12-109TAG with *Mb*Pyl(Bpa)RS/ *Mb*PyltRNA$_{CUA}$ pair in the presence of 1 mM Bpa, and D) 7D12-109TAG with *Mb*Pyl(Bpa)RS/ *Mb*PyltRNA$_{CUA}$ pair in the presence of 1 mM Bpa and 1 mM pcY. Mass spectra after deconvolution are shown in Figure 3e. The mass spectrometry data demonstrates that newly evolved *Mb*Pyl(Bpa)RS/ *Mb*PyltRNA$_{CUA}$ pair can selectively incorporate Bpa in the presence of equimolar amount of pcY. 18

Supplementary Figure 10: Expression of wt-7D12 using pSANG-oR-o7D12 plasmid. Coomassie stained gel image demonstrates that the expression of 7D12 is efficient and dependent on addition of IPTG. Lane marked L is the Invitrogen SeeBlue Plus2 Pre-stained Protein Standard (Catalog no. LC5925). This experiment was repeated twice with similar results. 19

Supplementary Figure 11: Raw mass spectrometry data before deconvolution for 7D12-32pcY-109Bpa. Mass spectrum after deconvolution is shown in Figure 4c. 20

Supplementary Figure 12: On-cell binding assay to measure the binding of 7D12-109Bpa and 7D12-32pcY-109Bpa towards MDA-MB-231 and SW620 cells. MDA-MB-231 and SW620 cells are used as negative control cell lines to assess the specificity of 7D12-109Bpa and 7D12-32pcY-109Bpa to EGFR. (A) Near background binding was observed for 7D12-

109Bpa and 7D12-32pcY-109Bpa towards the control cell lines, MDA-MB-231 and SW620, both before and after irradiation with 365 nm light. Binding assay of 7D12-109Bpa towards EGFR-positive, A431 cells, was performed as a control experiment. These results demonstrate that 7D12-109Bpa and 7D12-32pcY-109Bpa specifically bind to EGFR on cell surface. To ensure reproducibility, experiments were performed in duplicates represented as REP 1 and REP 2. These images were acquired using GE ImageQuant™ LAS 4000 gel imager. (B) Chemiluminescence intensities obtained from on-cell binding experiments were quantified using CLARIOstar plate reader. For each lane (i.e. each row), the intensity from each well was subtracted from intensity for zero concentration in that lane, i.e. I-I_0, and plotted against concentration of 7D12. This normalisation is performed to ensure that data between different cell lines, and between replicates could be compared. The data was plotted using GraphPad. Each point in the graph represents mean values of normalised intensities ± s.d, designated as error bar, from two independent replicates, REP 1 and REP 2 in Supplementary Figure 12A. The line shows connection between individual points. 21

Supplementary Figure 13: Sequence of o7D12 gene block for insertion into pSANG10 plasmid. 22

Supplementary Figure 14: Sequence of o7D12-32TAG-109AGTA gene block for insertion into pSANG-oR plasmid. 23

Supplementary Table 1: List of primers used in the present investigation. 24

Supplementary Figure 1: The sequence of *Mj*RS(Bpa) in pULTRA-Bpa plasmid.

```
ATGGACGAATTTGAAATGATAAAGAGAAACACATCTGAAATTATCAGCGAGGAA
M  D  E  F  E  M  I  K  R  N  T  S  E  I  I  S  E  E
GAGTTAAGAGAGGTTTTAAAAAAAGATGAAAAATCTGCTGGTATAGGTTTTGAA
E  L  R  E  V  L  K  K  D  E  K  S  A  G  I  G  F  E
CCAAGTGGTAAAATACATTTAGGGCATTATCTCCAAATAAAAAAGATGATTGAT
P  S  G  K  I  H  L  G  H  Y  L  Q  I  K  K  M  I  D
TTACAAAATGCTGGATTTGATATAATTATATTGTTGGCTGATTTACACGCCTAT
L  Q  N  A  G  F  D  I  I  I  L  L  A  D  L  H  A  Y
TTAAACCAGAAAGGAGAGTTGGATGAGATTAGAAAAATAGGAGATTATAACAAA
L  N  Q  K  G  E  L  D  E  I  R  K  I  G  D  Y  N  K
AAAGTTTTTGAAGCAATGGGGTTAAAGGCAAAATATCTTTATGGAAGTCCTTTC
K  V  F  E  A  M  G  L  K  A  K  Y  L  Y  G  S  P  F
CAGCTTGATAAGGATTATACACTGAATGTCTATAGATTGGCTTTAAAAACTACC
Q  L  D  K  D  Y  T  L  N  V  Y  R  L  A  L  K  T  T
TTAAAAAGAGCAAGAAGGAGTATGGAACTTATAGCAAGAGAGGATGAAAATCCA
L  K  R  A  R  R  S  M  E  L  I  A  R  E  D  E  N  P
AAGGTTGCTGAAGTTATCTATCCAATAATGCAGGTTAATACGAGTCATTATCTG
K  V  A  E  V  I  Y  P  I  M  Q  V  N  T  S  H  Y  L
GGCGTTGATGTTGCAGTTGGAGGGATGGAGCAGAGAAAAATACACATGTTAGCA
G  V  D  V  A  V  G  G  M  E  Q  R  K  I  H  M  L  A
AGGGAGCTTTTACCAAAAAAGGTTGTTTGTATTCACAACCCTGTCTTAACGGGT
R  E  L  L  P  K  K  V  V  C  I  H  N  P  V  L  T  G
TTGGATGGAGAAGGAAAGATGAGTTCTTCAAAAGGGAATTTTATAGCTGTTGAT
L  D  G  E  G  K  M  S  S  S  K  G  N  F  I  A  V  D
GACTCTCCAGAAGAGATTAGGGCTAAGATAAAGAAAGCATACTGCCCAGCTGGA
D  S  P  E  E  I  R  A  K  I  K  K  A  Y  C  P  A  G
GTTGTTGAAGGAAATCCAATAATGGAGATAGCTAAATACTTCCTTGAATATCCT
V  V  E  G  N  P  I  M  E  I  A  K  Y  F  L  E  Y  P
TTAACCATAAAAAGGCCAGAAAAATTTGGTGGAGATTTGACAGTTAATAGCTAT
L  T  I  K  R  P  E  K  F  G  G  D  L  T  V  N  S  Y
GAGGAGTTAGAGAGTTTATTTAAAAATAAGGAATTGCATCCAATGCGCTTAAAA
E  E  L  E  S  L  F  K  N  K  E  L  H  P  M  R  L  K
AATGCTGTAGCTGAAGAACTTATAAAGATTTTAGAGCCAATTAGAAAGAGATTA
N  A  V  A  E  E  L  I  K  I  L  E  P  I  R  K  R  L
TAA
-
```

Supplementary Figure 2: Raw mass spectrometry data before deconvolution for: A) wt-7D12, B) 7D12-32**Bpa**, C) 7D12-109**Bpa**, and D) 7D12-113**Bpa**. Mass spectra after deconvolution are shown in Figure 1c.

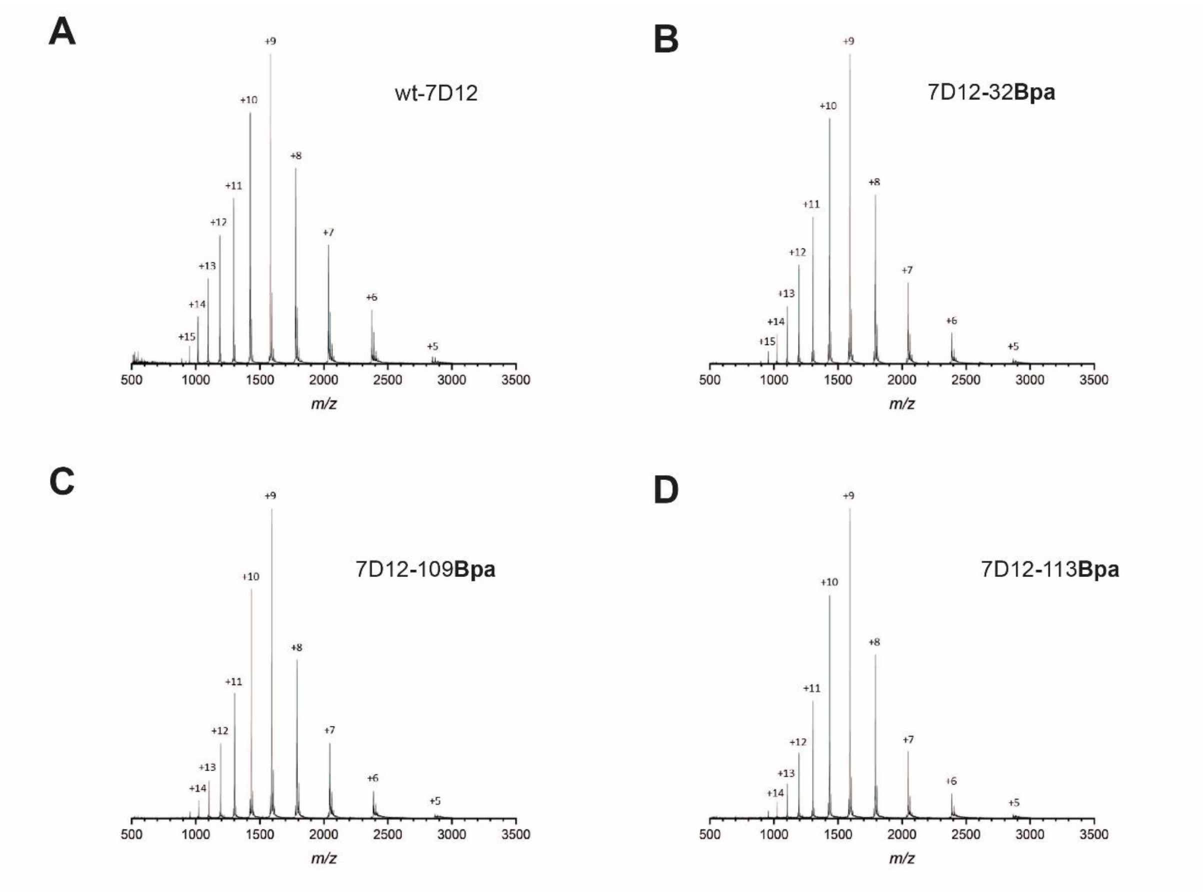

Supplementary Figure 3: Effect of 365 nm irradiation on the viability of A431 cells assessed using alamarBlue cell viability assay (see **Methods**). After adding the alamarBlue reagents, the fluorescence emission at 590 nm (with excitation at 560 nm) was quantified using CLARIOstar plate reader (BMG labtech). The fluorescence intensity of a standard containing no cells was subtracted from the fluorescence intensity from each well. Subsequently, these intensities were normalised by dividing the intensity values by mean intensity value obtained from no irradiation control experiment, and plotted as a bar graph. Six replicates of each experiment were performed. The normalised intensities from each of these replicates are shown as circles in the bar graph. The top of each bar in the bar graph represents mean values of normalised intensities ± s.d, designated as error bar, from six independent replicates. The results demonstrate that greater than 90% of the cells are viable upon 10 min irradiation with 365 nm light.

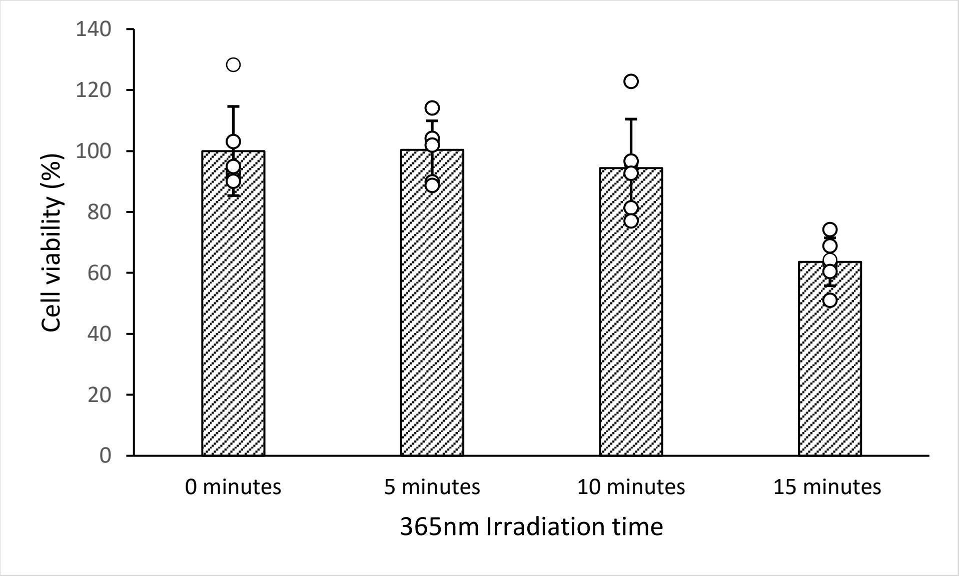

Supplementary Figure 4. Liquid chromatography tandem mass spectrometry (MS/MS) analysis of the photocrosslinked 7D12-109**Bpa**-sEGFR complex demonstrates the presence of 7D12 in the crosslinked product. The 7D12 sequence is covered by 14 unique peptides (73% of the sequence) highlighted in grey. Location of 109**Bpa** (B) is highlighted in cyan. Representative MS/MS spectra of detected peptides matching the 7D12 sequence are also shown. Assigned b- and y-ions are annotated in red and blue, respectively. Ions belonging to other series, e.g. a-ions, are shown in green. Data was visualised using Scaffold 5 (see **Methods** for further details).

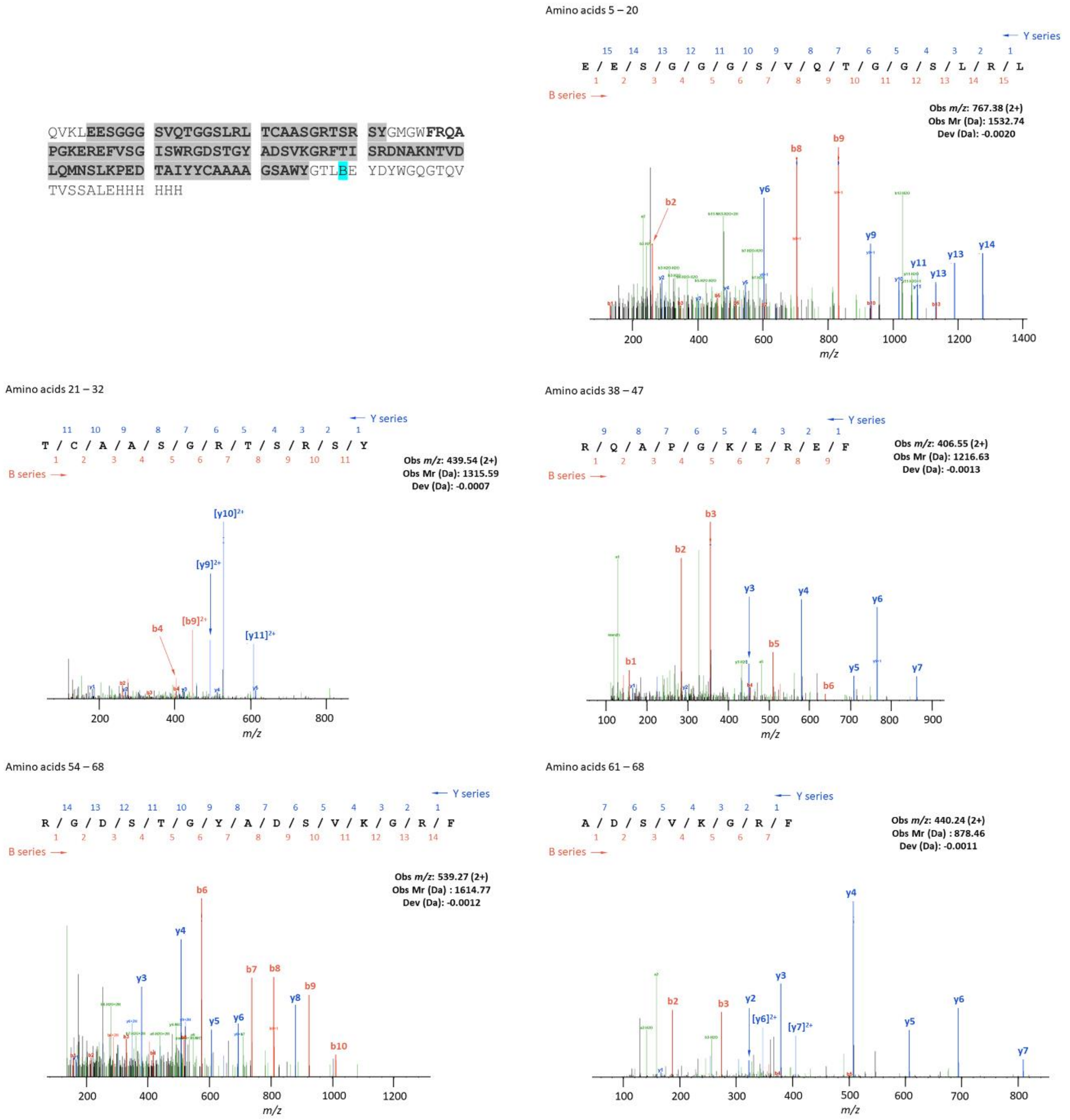

Amino acids 69 - 81

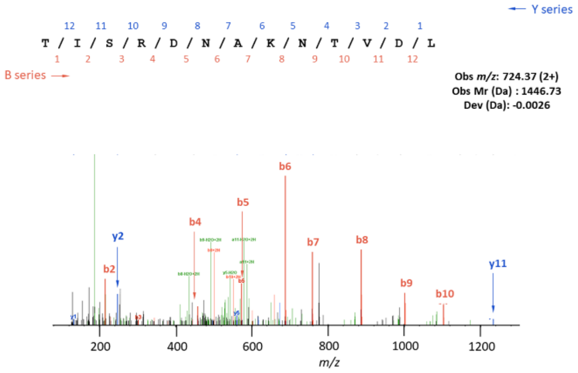

Amino acids 87 – 94

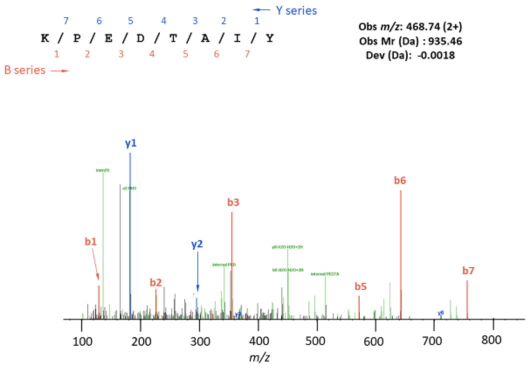

Amino acids 96 – 105

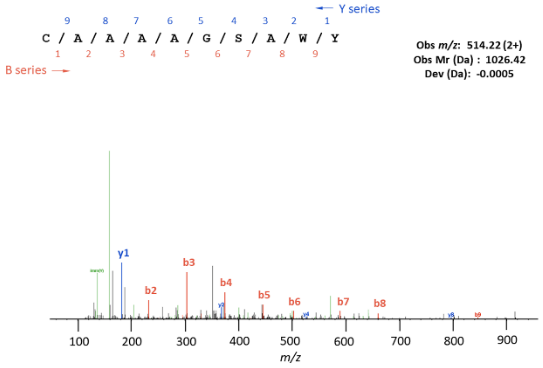

Supplementary Figure 5. Liquid chromatography tandem mass spectrometry (MS/MS) analysis of the photocrosslinked 7D12-109**Bpa**-sEGFR complex demonstrates presence of EGFR in the crosslinked product. Protein sequence of EGFR showing detected peptides (49 unique peptides, covering 62% of the sequence; highlighted in grey). Representative MS/MS spectra of detected peptides matching the EGFR sequence are also shown. Assigned b- and y-ions are annotated in red and blue, respectively. Ions belonging to other series, e.g. a-ions, are shown in green. Data was visualised using Scaffold 5 (see **Methods** for further details).

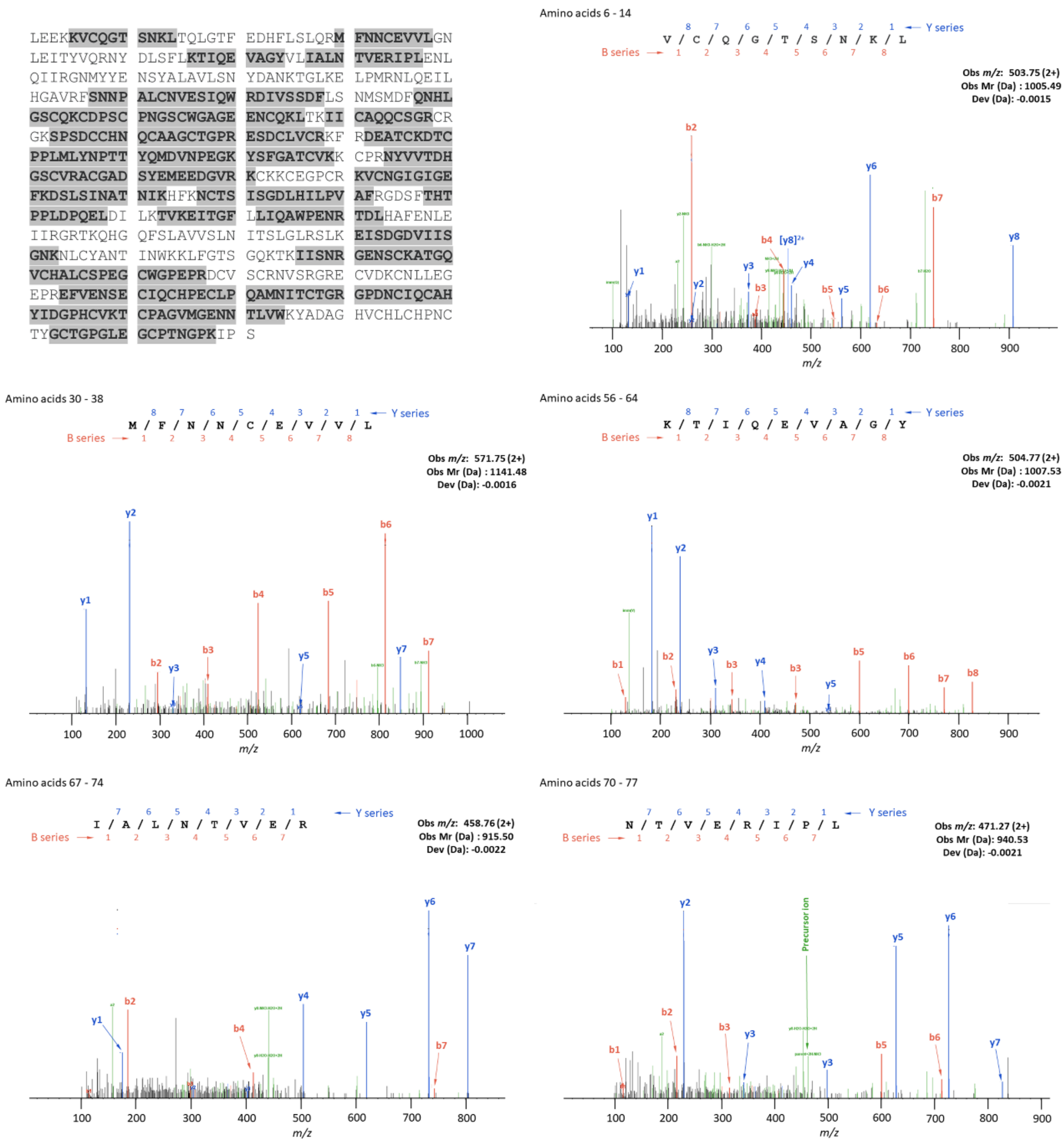

Amino acids 127 - 140

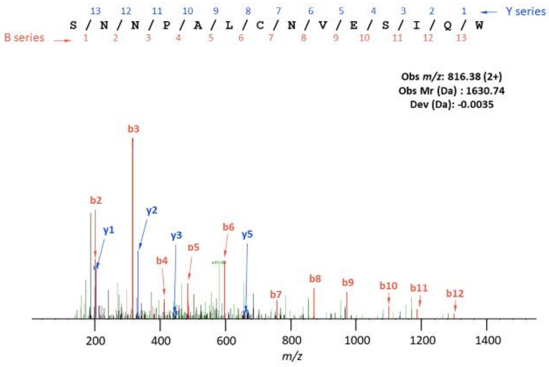

Amino acids 141 - 148

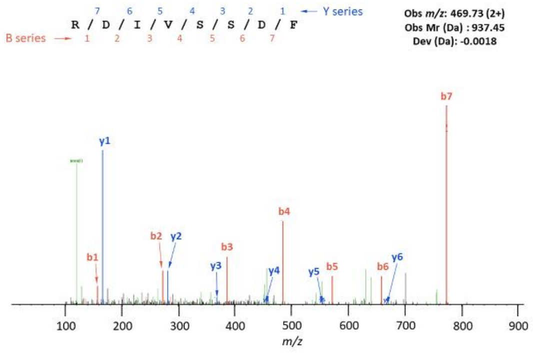

Amino acids 157 - 165

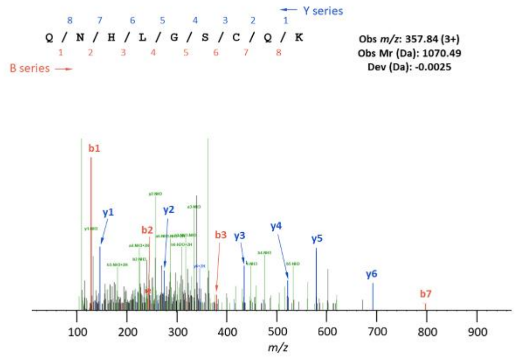

Amino acids 166 - 176

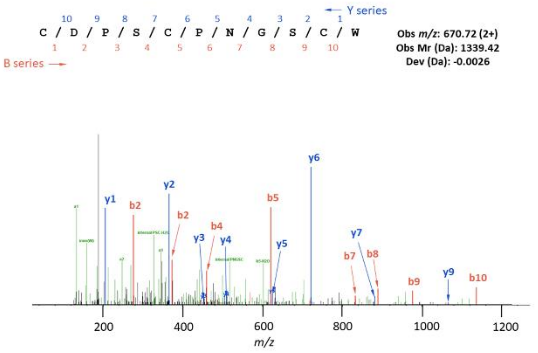

Amino acids 177 - 186

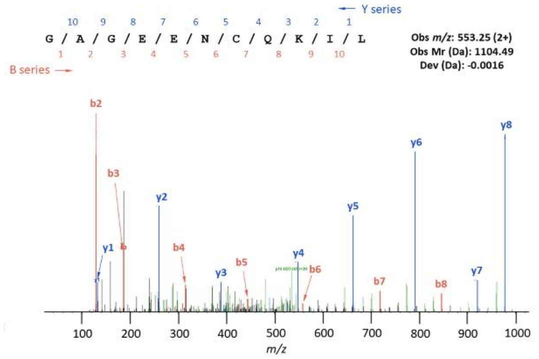

Amino acids 189 - 198

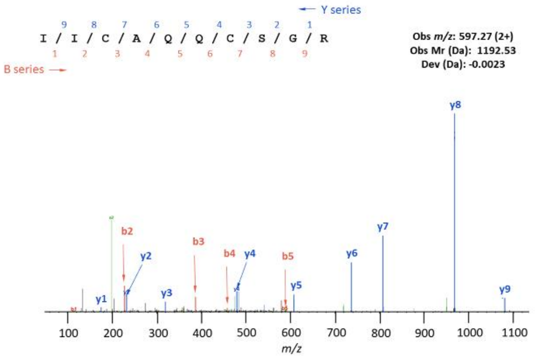

Amino acids 203 - 220

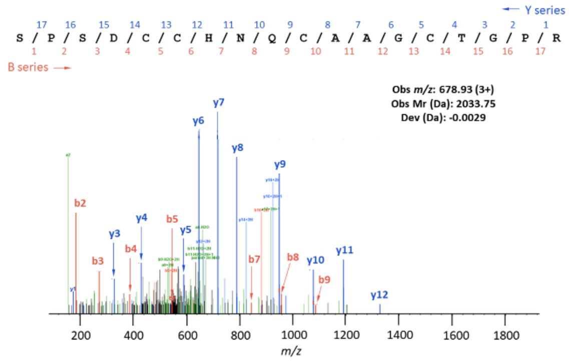

Amino acids 221 - 228

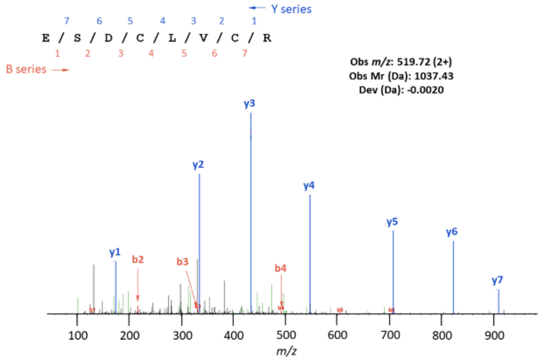

Amino acids 232 - 243

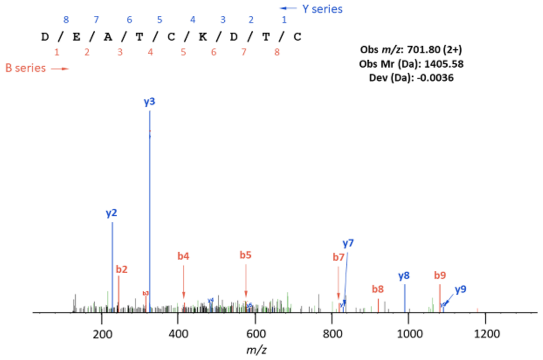

Amino acids 238 - 245

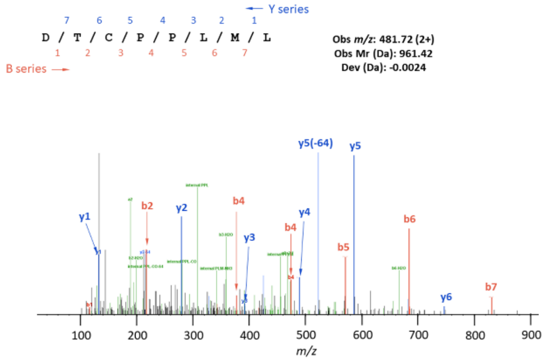

Amino acids 252 - 260

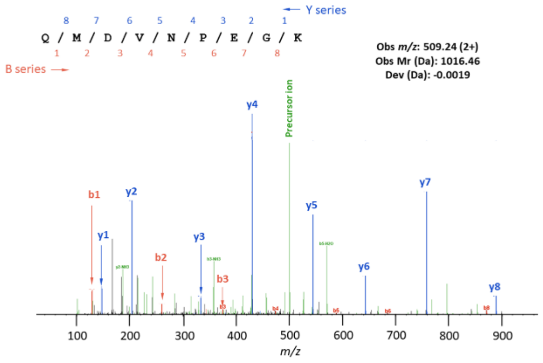

Amino acids 262 - 269

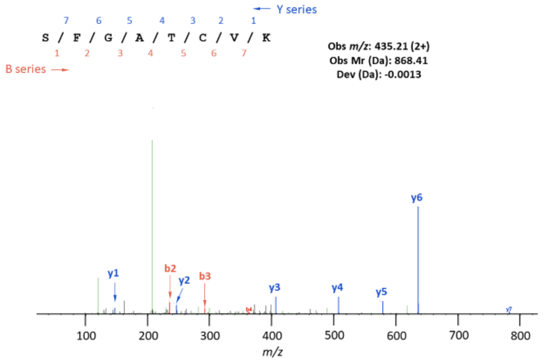

Amino acids 276 – 285
(m/z 565)

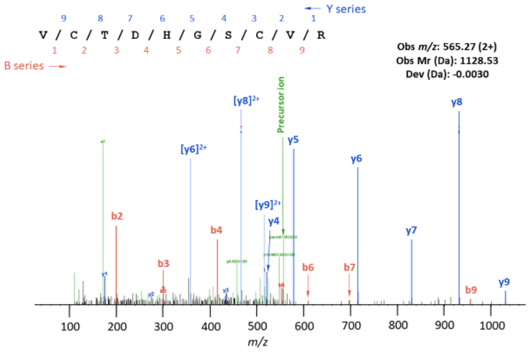

Amino acids 286 - 300

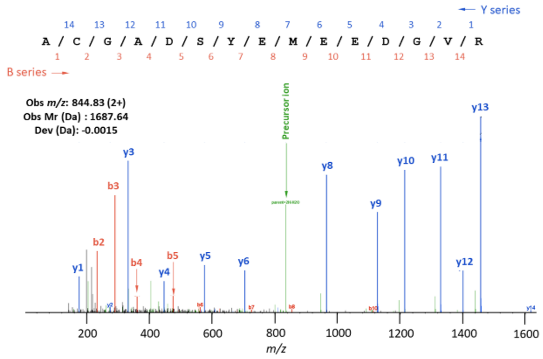

Amino acids 311 - 321

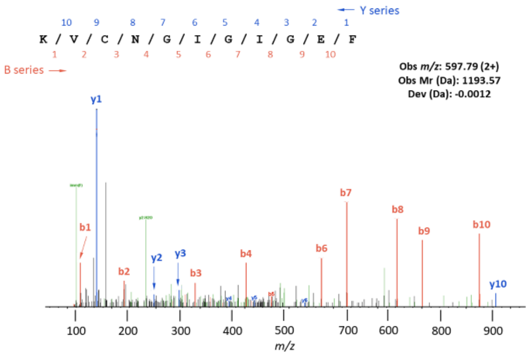

Amino acids 323 - 333

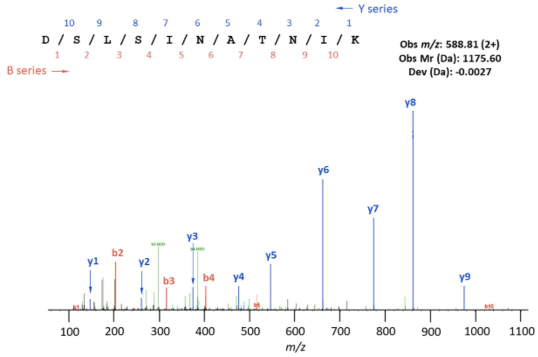

Amino acids 337 - 352

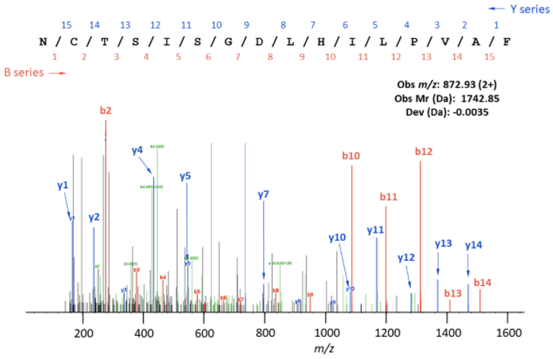

Amino acids 358 - 368

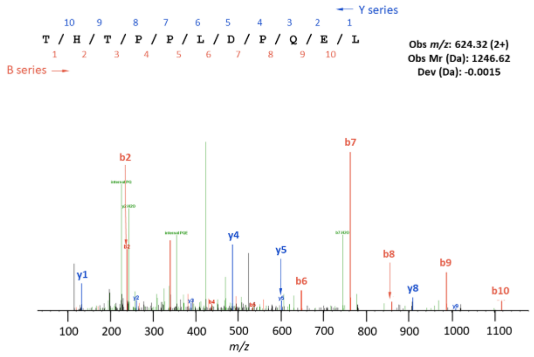

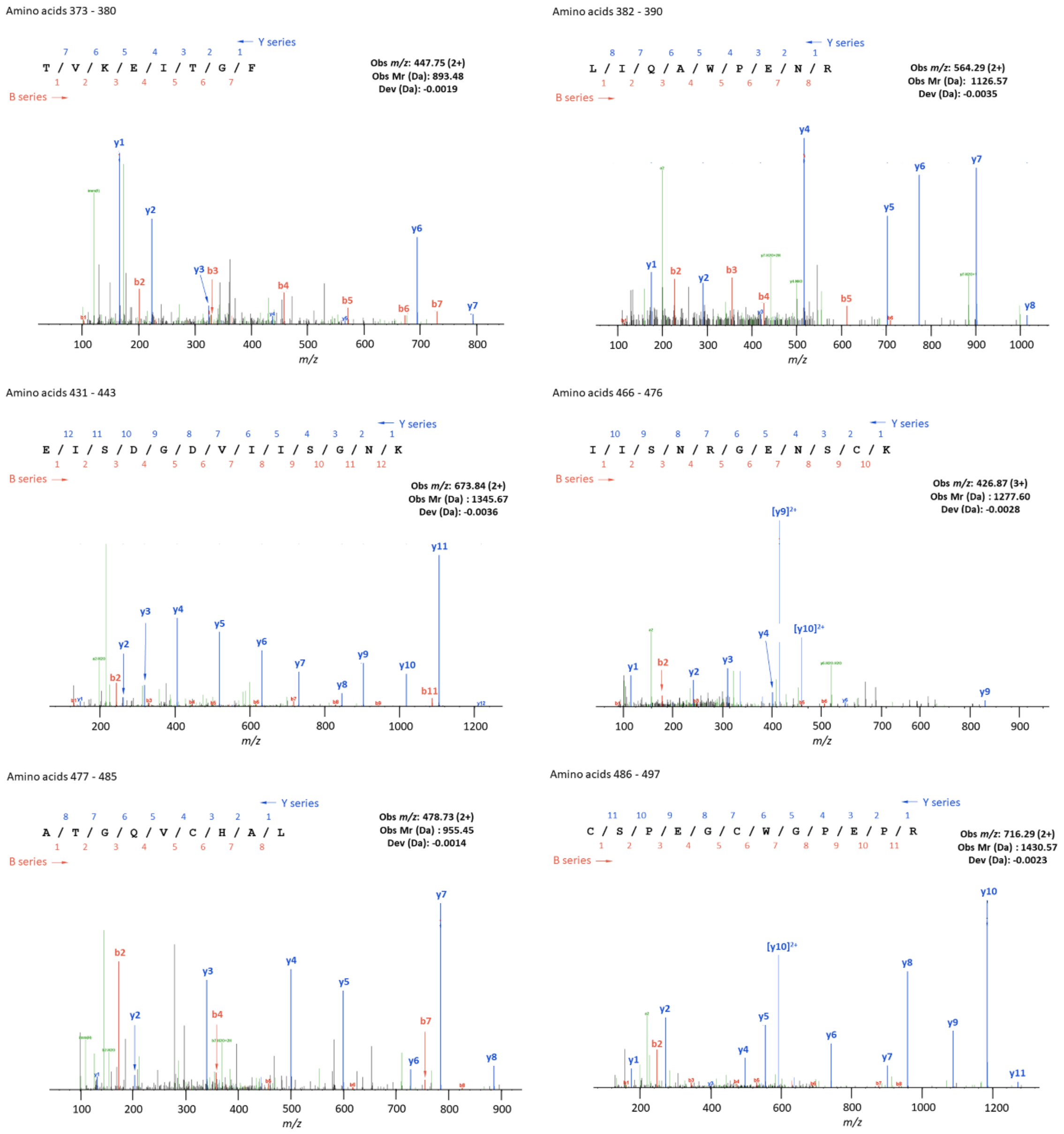
Amino acids 373 - 380
T / V / K / E / I / T / G / F
Obs m/z: 447.75 (2+)
Obs Mr (Da): 893.48
Dev (Da): -0.0019
Amino acids 382 - 390
L / I / Q / A / W / P / E / N / R
Obs m/z: 564.29 (2+)
Obs Mr (Da): 1126.57
Dev (Da): -0.0035
Amino acids 431 - 443
E / I / S / D / G / D / V / I / I / S / G / N / K
Obs m/z: 673.84 (2+)
Obs Mr (Da) : 1345.67
Dev (Da): -0.0036
Amino acids 466 - 476
I / I / S / N / R / G / E / N / S / C / K
Obs m/z: 426.87 (3+)
Obs Mr (Da) : 1277.60
Dev (Da): -0.0028
Amino acids 477 - 485
A / T / G / Q / V / C / H / A / L
Obs m/z: 478.73 (2+)
Obs Mr (Da) : 955.45
Dev (Da): -0.0014
Amino acids 486 - 497
C / S / P / E / G / C / W / G / P / E / P / R
Obs m/z: 716.29 (2+)
Obs Mr (Da) : 1430.57
Dev (Da): -0.0023
Y series
B series
m/z

Amino acids 524 - 550

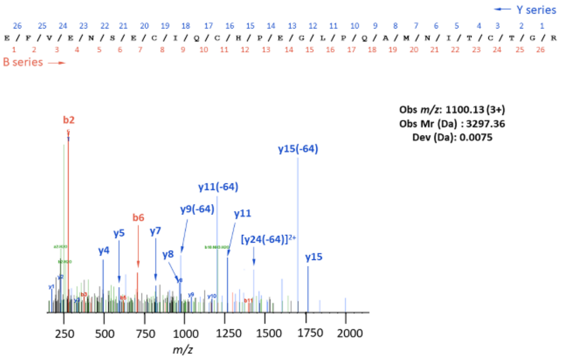

Amino acids 551 - 561

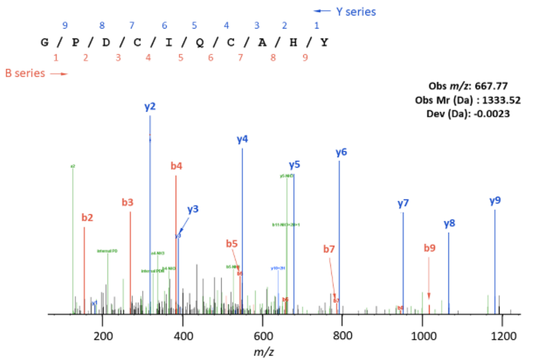

Amino acids 570 – 584

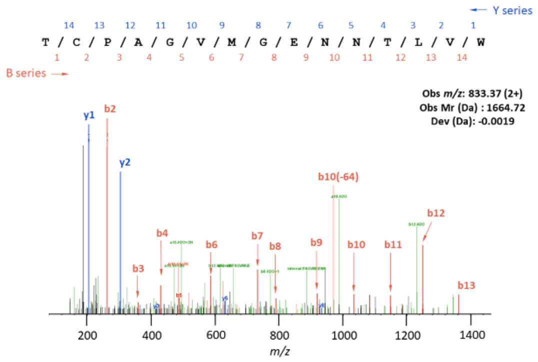

Amino acids 603 - 618

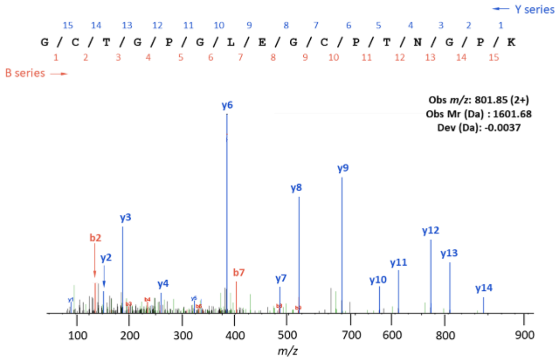

Supplementary Figure 6: Assessing the selectivity of *Mj*RS(pcY)/*Mj*tRNA$_{CUA}$, and *Mj*RS(Bpa)/*Mj*tRNA$_{CUA}$ at site-specific incorporation of **pcY** and **Bpa** at position 109 in 7D12. For *Mj*RS(pcY)/*Mj*tRNA$_{CUA}$, band corresponding to full-length 7D12 is only observed when the expression is performed in the presence of **pcY**, demonstrating that *Mj*RS(pcY) is specific for **pcY**. For *Mj*RS(Bpa)/*Mj*tRNA$_{CUA}$, band corresponding to full 7D12 is observed when the expression is performed in the presence of **pcY** or **Bpa** or without any non-canonical amino acid, demonstrating that *Mj*RS(Bpa) is promiscuous. These gel images are obtained after Coomassie staining. Lane marked L' is the Thermo Scientific PageRuler Unstained Low Range Protein Ladder (Catalog no. 26632). This experiment was repeated twice with similar results.

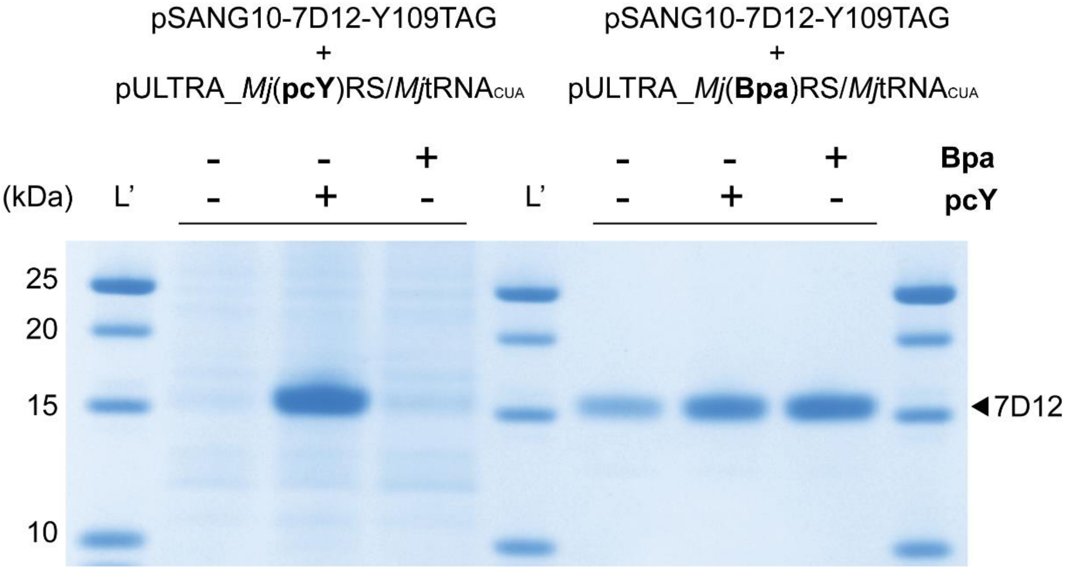

Supplementary Figure 7: Screening of *Mb*PylRS mutants obtained after three rounds of directed evolution to isolate novel *Mb*PylRS mutants for site-specific incorporation of **Bpa**. 192 colonies (or *Mb*PylRS mutants) were screened for efficient site-specific incorporation of **Bpa** at position 111 in chloramphenicol acetyl transferase (CAT). Two colonies, A2 on Plate I and E10 on Plate II (circled red) survived on chloramphenicol concentration up to 300 μg/ml in the presence of 1 mM **Bpa**, but for these colonies no growth was observed in the absence of **Bpa** on chloramphenicol concentration at and above 100 μg/ml. The results demonstrate that *Mb*PylRS mutants in A2 and E10 are likely to efficiently incorporate **Bpa** but no other naturally occurring canonical amino acid.

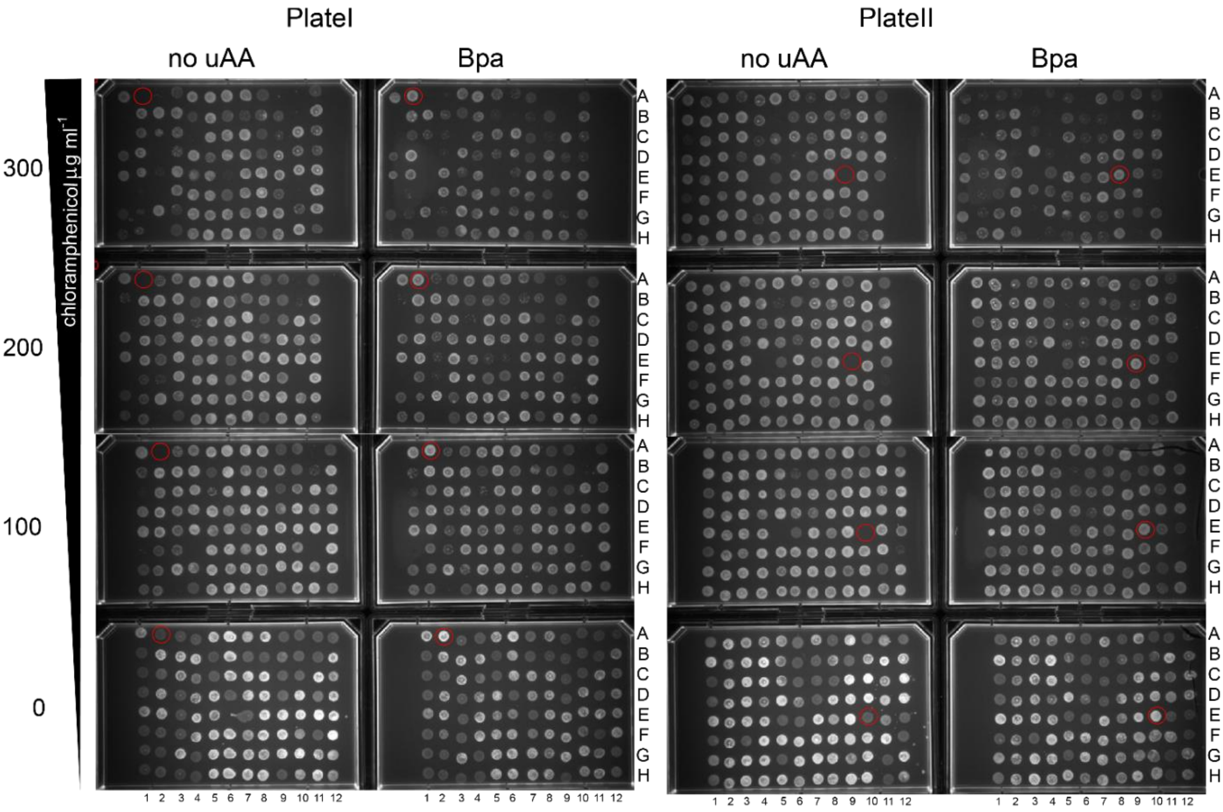

Supplementary Figure 8: Assessing the efficiency and substrate specificity of newly evolved *Mb*Pyl(Bpa)RS and previously known *Mm*Pyl(Bpa)RS, BpaRS1 (Chembiochem, 2013. 14(16): p. 2100-5), at site-specific incorporation of **Bpa**. Expression of *gst-1TAG-cam* gene is performed with *Mb*Pyl(Bpa)RS, and BpaRS1 either without any noncanonical amino acid (ncAA), or with p-benzoyl-L-phenylalanine (**Bpa**), O-(2-Nitrobenzyl)-L-tyrosine (photocaged tyrosine, **pcY**), p-Azido-L-phenylalanine (**AzF**) or N6-(tert-butoxycarbonyl)-L-lysine (**BocK**). As a control, expression of *gst-1TAG-cam* gene was also performed with wt-*Mb*PylRS that is known to efficiently incorporate **BocK**. Site-specific incorporation of amino acid in response to TAG stop codon will result in the full-length Gst-CaM (top band). A) Comparison of band intensities of full length Gst-CaM for expressions performed with **Bpa** (1mM), **pcY**(1mM), **AzF**(1mM) and **BocK**(1mM) using *Mm*Pyl(Bpa)RS and newly evolved *Mb*Pyl(Bpa)RS demonstrates that latter is highly efficient and specific at incorporating Bpa in Gst-CaM. This gel image is obtained after Coomassie staining. B) ncAA incorporation efficiency is calculated by taking the ratio of intensity of the top band (Gst-CaM) to the sum of intensities of top band (Gst-CaM) and lower band (Gst). The efficiency of *Mb*Pyl(Bpa)RS at incorporating **Bpa** and that of wt-*Mb*PylRS at incorporating **BocK** are similar. Also, *Mb*Pyl(Bpa)RS is 5-fold selective for **Bpa** over **pcY**. Lane marked L is the Invitrogen SeeBlue Plus2 Pre-stained Protein Standard (Catalog no. LC5925). These experiments were repeated twice with similar results.

A)

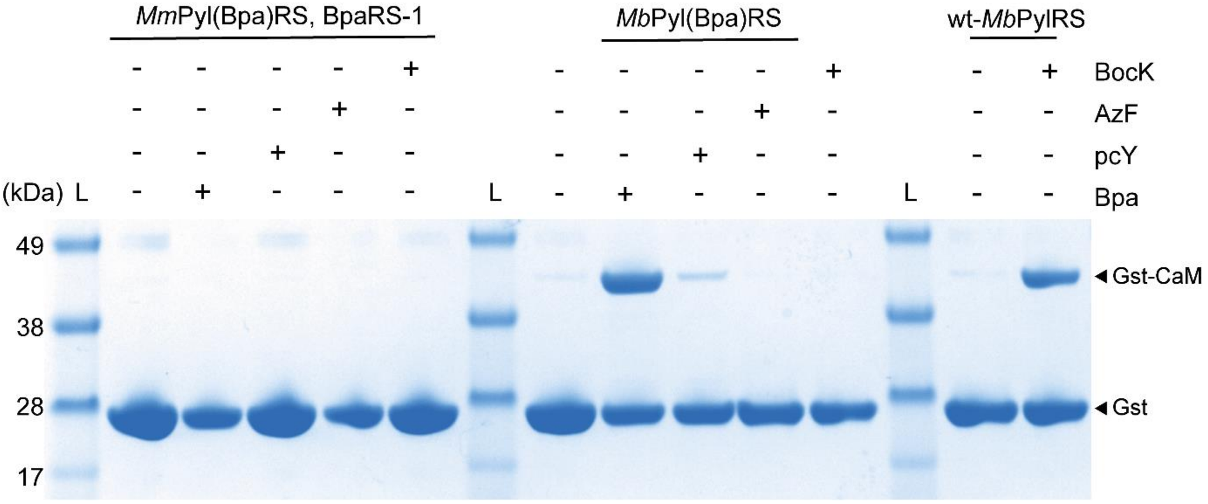

B)

**aaRS/tRNA pair**	*Mb*Pyl(Bpa)RS/ *Mb*PyltRNA$_{CUA}$		wt-*Mb*PylRS/ *Mb*PyltRNA$_{CUA}$
**ncAA**	**Bpa**	**pcY**	**BocK**
**ncAA incorporation efficiency**	49%	9.5%	40%

Supplementary Figure 9: Raw mass spectrometry data before deconvolution for protein samples obtained after purification for expression of: A) 7D12-32TAG with *Mb*Pyl(Bpa)RS/ *Mb*PyltRNA$_{CUA}$ pair in the presence of 1 mM **Bpa**, B) 7D12-32TAG with *Mb*Pyl(Bpa)RS/ *Mb*PyltRNA$_{CUA}$ pair in the presence of 1 mM **Bpa** and 1 mM **pcY**, C) 7D12-109TAG with *Mb*Pyl(Bpa)RS/ *Mb*PyltRNA$_{CUA}$ pair in the presence of 1 mM **Bpa**, and D) 7D12-109TAG with *Mb*Pyl(Bpa)RS/ *Mb*PyltRNA$_{CUA}$ pair in the presence of 1 mM **Bpa** and 1 mM **pcY**. Mass spectra after deconvolution are shown in Figure 3e. The mass spectrometry data demonstrates that newly evolved *Mb*Pyl(Bpa)RS/ *Mb*PyltRNA$_{CUA}$ pair can selectively incorporate **Bpa** in the presence of equimolar amount of **pcY**.

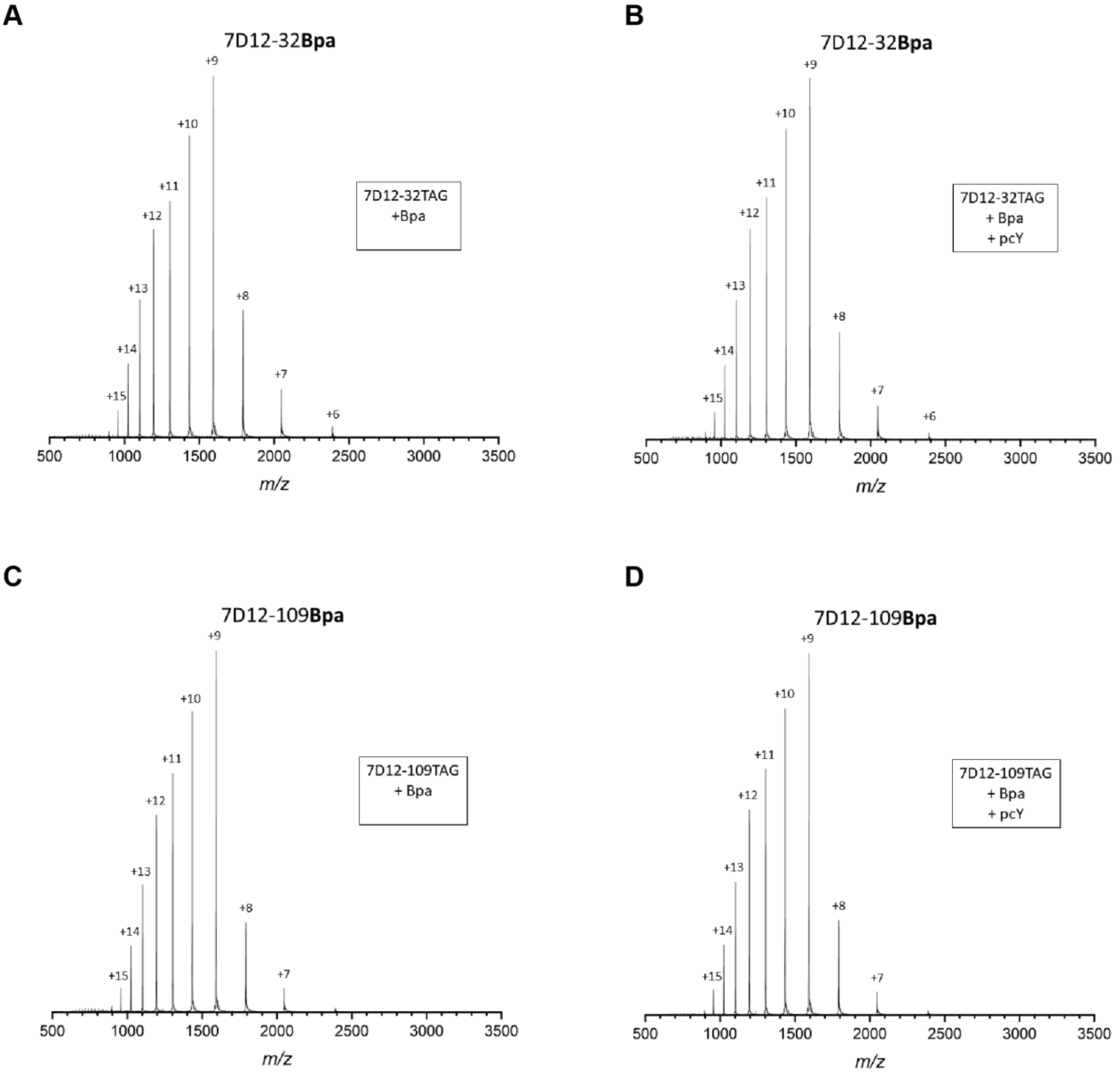

Supplementary Figure 10: Expression of wt-7D12 using pSANG-oR-o7D12 plasmid. Coomassie stained gel image demonstrates that the expression of 7D12 is efficient and dependent on addition of IPTG. Lane marked L is the Invitrogen SeeBlue Plus2 Pre-stained Protein Standard (Catalog no. LC5925). This experiment was repeated twice with similar results.

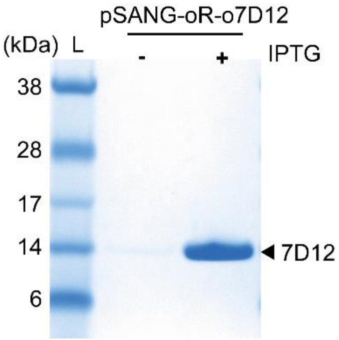

Supplementary Figure 11: Raw mass spectrometry data before deconvolution for 7D12-32**pcY**-109**Bpa**. Mass spectrum after deconvolution is shown in Figure 4c.

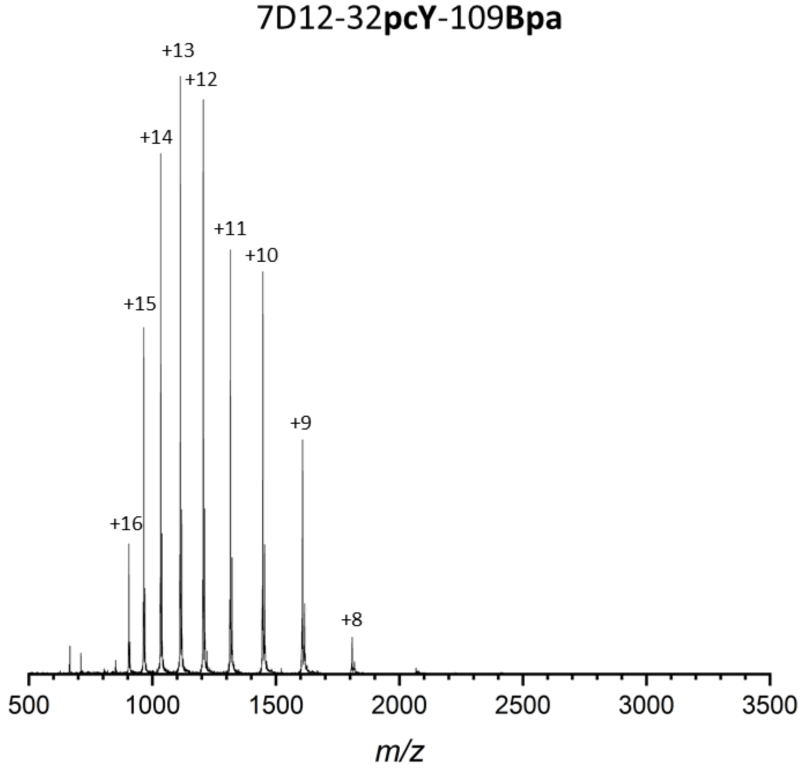

Supplementary Figure 12: On-cell binding assay to measure the binding of 7D12-109**Bpa** and 7D12-32**pcY**-109**Bpa** towards MDA-MB-231 and SW620 cells. MDA-MB-231 and SW620 cells are used as negative control cell lines to assess the specificity of 7D12-109**Bpa** and 7D12-32**pcY**-109**Bpa** to EGFR. (A) Near background binding was observed for 7D12-109**Bpa** and 7D12-32**pcY**-109**Bpa** towards the control cell lines, MDA-MB-231 and SW620, both before and after irradiation with 365 nm light. Binding assay of 7D12-109**Bpa** towards EGFR-positive, A431 cells, was performed as a control experiment. These results demonstrate that 7D12-109**Bpa** and 7D12-32**pcY**-109**Bpa** specifically bind to EGFR on cell surface. To ensure reproducibility, experiments were performed in duplicates represented as REP 1 and REP 2. These images were acquired using GE ImageQuant™ LAS 4000 gel imager. (B) Chemiluminescence intensities obtained from on-cell binding experiments were quantified using CLARIOstar plate reader. For each lane (i.e. each row), the intensity from each well was subtracted from intensity for zero concentration in that lane, i.e. I-I_0, and plotted against concentration of 7D12. This normalisation is performed to ensure that data between different cell lines, and between replicates could be compared. The data was plotted using GraphPad. Each point in the graph represents mean values of normalised intensities ± s.d, designated as error bar, from two independent replicates, REP 1 and REP 2 in Supplementary Figure 12A. The line shows connection between individual points.

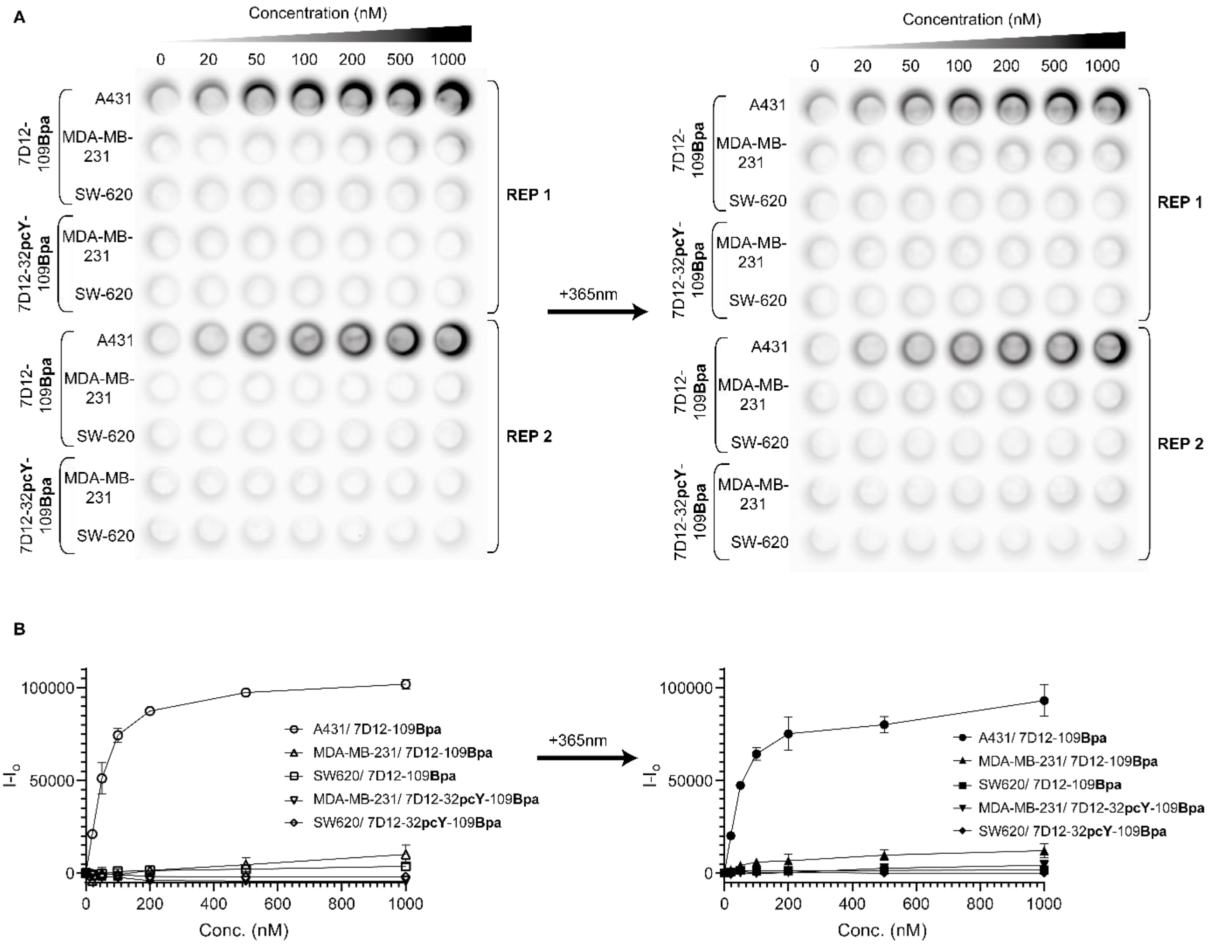

Supplementary Figure 13: Sequence of o7D12 gene block for insertion into pSANG10 plasmid.

GAGCGGATAACAATTCCCCTCTAGATGAGCGGCTCGAGACAATTTTCATATCCCTCCGCAAA
TGAAATCGCTGATTACTCCGATTGCTGCCGGATTACTGTTGGCGTTTTCCCAATACTCGTTG
GCGCAGGTAAAATTAGAAGAATCTGGGGGTGGGTCCGTTCAGACGGGTGGAAGTCTGCGCTT
GACATGTGCCGCGTCTGGGCGCACATCACGCTCATATGGAATGGGCTGGTTCCGTCAAGCTC
CTGGCAAGGAGCGTGAGTTCGTATCCGGTATCTCATGGCGCGGTGACTCAACCGGATATGCT
GACTCAGTAAAGGGGCGTTTCACCATCTCTCGTGATAATGCGAAGAATACTGTCGACTTACA
AATGAACTCACTTA

Supplementary Figure 14: Sequence of o7D12-32TAG-109AGTA gene block for insertion into pSANG-oR plasmid.

CCAGCAACCGCACCTGTGGCGCCGGTGATGCCGGCCACGATGCGTCCGGCGTAGAGGATCGAGATCTC

GATCCCGCGAAATTAATACGACTCACTATAGGGGAATTGTGAGCGGATAACAATTCCCCTCTAGATGA

GCGGCTCGAGACAATTTTCATATCCCTCCGCAAATGAAATCGCTGATTACTCCGATTGCTGCCGGATT

ACTGTTGGCGTTTTCCCAATACTCGTTGGCGCAGGTAAAATTAGAAGAATCTGGGGGTGGGTCCGTTC

AGACGGGTGGAAGTCTGCGCTTGACATGTGCCGCGTCTGGGCGCACATCACGCTCATAGGGAATGGGC

TGGTTCCGTCAAGCTCCTGGCAAGGAGCGTGAGTTCGTATCCGGTATCTCATGGCGCGGTGACTCAAC

CGGATATGCTGACTCAGTAAAGGGGCGTTTCACCATCTCTCGTGATAATGCGAAGAATACTGTCGACT

TACAAATGAACTCACTTAAACCAGAGGATACCGCTATTTACTACTGCGCTGCTGCGGCAGGGTCTGCA

TGGTACGGTACACTGAGTAGAGTATGACTACTGGGGGCAGGGCACCCAGGTCACGGTTTCTTCTCACC

ACCATCACCACCACTGATAAAAGCTTTAATAAGTCGAGCACCACCACCACCACCACTGAGATCCGGCT

GCTAACAAAGCCCGAAAGGAAGCTGAGTTGGCTGCTGCCACCGCTGAGCAATAACTAGCATAACCCCT

TG

Supplementary Table 1: List of primers used in the present investigation.

Primer name	Primer no. (UEA Dxxxx)	Primer sequence
Mb_pylRS_Y349F_F	UEA D356	ATGGTCTCATTTGGCGATACCCTGGATATTATGC
Mb_pylRS_Y349F_R	UEA D357	ATGGTCTCACAAACACCATGCAGCTATCGCCC
N311_C313_F	UEA D326	ATGGTCTCAGGTTNNKTTTNNKCAAATGGGCAGCGGCTGC
N311_C313_R	UEA D327	ATGGTCTCAAACCATGGTGAATTCTTCCAGGTG
MbpylRS_W382_G386X_F	UEA D378	ATGGTCTCAATTGGCGCGNNKTTTGGCCTGGAACGTCTGC
MbpylRS_W382_G386X_R	UEA D379	ATGGTCTCACAATMNNCGGTTTATCAATGCCCCATTCAC
gst-cam_pREP_F	UEA D354	TTTGGCGAAAATGAGACGTTGATCGGCACGCGGCCGCAATTAATGTGAGTTAGCTCACTC
gst-cam_pREP_R	UEA D355	ATTACGCCCCGCCCTGCCACTCATCGCAGTGCGGCCGCTTAGTGATGGTGATGGTGATGC
REP_S3	UEA D263	ATCAGTAAGTTGGCAGCATC
REP_S16	UEA D302	CCCTGCACCATTATGTTCCG
AS61_to_pSang_F	UEA D496	GGGGACTGTTGGGCGCCATCTCCTTGCATGGGATCCTCGGGAGTTGTCAG
orRNA_RSF_SANG_F	UEA D047	CACCCGTGGGGCCGCCATGCCTGCAGCGCCGACATCATAACGGTTCT
orRNA_RSF_SANG_R	UEA D048	GCAGGCCATTATCGCCGGCAGGATCCTGTAGATATGACGACAGGAA
o-pSANG_AS61_BamHI_f	UEA D499	TACAAACTCTTCCTGTCGTCATATCTACAGGATCCTCGGGAGTTGTCAGC
o-pSANG_AS61_BamHI_r	UEA D500	CGGCGAGAAGCAGGCCATTATCGCCGGCAGCGGCCGCGTTGGGTAACGCCAGGGTTTTC
