# Supplementary material for: Site-specific encoding of photoactivity and photoreactivity into antibody fragments
Source: Nat Chem Biol. 2023 Feb 16;19(6):740–9. doi: 10.1038/s41589-022-01251-9 (PMC10229432; doi:10.1038/s41589-022-01251-9)
Supplement: Supplementary file 2 — Reporting Summary [file 41589_2022_1251_MOESM2_ESM.pdf]

nature research

Corresponding author(s): Amit Sachdeva

Last updated by author(s): Nov 25, 2022

Reporting Summary

Nature Research wishes to improve the reproducibility of the work that we publish. This form provides structure for consistency and transparency in reporting. For further information on Nature Research policies, see our Editorial Policies and the Editorial Policy Checklist.

Statistics

For all statistical analyses, confirm that the following items are present in the figure legend, table legend, main text, or Methods section.

n/a	Confirmed	
☐	☒	The exact sample size (*n*) for each experimental group/condition, given as a discrete number and unit of measurement
☐	☒	A statement on whether measurements were taken from distinct samples or whether the same sample was measured repeatedly
☒	☐	The statistical test(s) used AND whether they are one- or two-sided<br>*Only common tests should be described solely by name; describe more complex techniques in the Methods section.*
☒	☐	A description of all covariates tested
☒	☐	A description of any assumptions or corrections, such as tests of normality and adjustment for multiple comparisons
☐	☒	A full description of the statistical parameters including central tendency (e.g. means) or other basic estimates (e.g. regression coefficient) AND variation (e.g. standard deviation) or associated estimates of uncertainty (e.g. confidence intervals)
☒	☐	For null hypothesis testing, the test statistic (e.g. *F*, *t*, *r*) with confidence intervals, effect sizes, degrees of freedom and *P* value noted<br>*Give P values as exact values whenever suitable.*
☒	☐	For Bayesian analysis, information on the choice of priors and Markov chain Monte Carlo settings
☒	☐	For hierarchical and complex designs, identification of the appropriate level for tests and full reporting of outcomes
☒	☐	Estimates of effect sizes (e.g. Cohen's *d*, Pearson's *r*), indicating how they were calculated

Our web collection on statistics for biologists contains articles on many of the points above.

Software and code

Policy information about availability of computer code

Data collection	SDS-PAGE gels and on-cell assay data was collected using BioRad Gel Doc XR+ gel imager, BioRad ChemiDoc XRS+ gel imager, and GE ImageQuant™ LAS 4000 gel imager. On-cell assay quantitative data was collected using CLARIOstar plate reader (BMG labtech). Mass spectrometry data was collected using Bruker microQTOF-QIII mass spectrometer. LC-MS/MS measurements were performed on an Orbitrap Eclipse™ TribridTM mass spectrometer (Thermo Fisher, Hemel Hempstead, UK equipped with an UltiMate™ 3000 RSLCnano System (Thermo Fisher) using a nanoEase M/Z column (HSS C18 T3, 100 Å, 1.8 µm; Waters, Wilmslow, UK).
Data analysis	For SDS-PAGE analysis we used Image Lab (Version 6.1.0, build 7). For DNA sequence analysis we used Staden 2.0.0b11-2016, and SnapGene. For graph generation we used Microsoft Excel (Version 2210, Build 16.0.15726.20188) and Graphpad Prism 9. MS/MS data analysis was performed using Scaffold version 5.1.2 (Proteome Software Inc., Portland, OR). Protein structure images were produced using UCSF chimera 1.16.

For manuscripts utilizing custom algorithms or software that are central to the research but not yet described in published literature, software must be made available to editors and reviewers. We strongly encourage code deposition in a community repository (e.g. GitHub). See the Nature Research guidelines for submitting code & software for further information.

Data

Policy information about availability of data

All manuscripts must include a data availability statement. This statement should provide the following information, where applicable:

- Accession codes, unique identifiers, or web links for publicly available datasets
- A list of figures that have associated raw data
- A description of any restrictions on data availability

All data supporting the finding reported in this manuscript is available in the main manuscript, and supplementary information. Raw data is available as source data

files. Crystal structure of 7D12-EGFR complex was accessed via protein data bank, PDB ID: 4KRL. Crystal structure of PylRS with adenylated pyrrolysine was accessed via protein data bank, PDB ID: 2Q7H.

Field-specific reporting

Please select the one below that is the best fit for your research. If you are not sure, read the appropriate sections before making your selection.

☒ Life sciences ☐ Behavioural & social sciences ☐ Ecological, evolutionary & environmental sciences

For a reference copy of the document with all sections, see nature.com/documents/nr-reporting-summary-flat.pdf

Life sciences study design

All studies must disclose on these points even when the disclosure is negative.

Sample size	The sample size for on-cell assay was determined based on previous experience with similar experiments (Angewandte Chemie Int. Ed., Vol. 58, Pg. 17986.). Most other experiments were performed in duplicates with several repeats between experiments. In addition, the conclusions made in the manuscript are supported by multiple distinct experiments proving the same thing. Sample size for experiments was not predetermined but chosen based on convention in field. Also, as the variation in the assays was small and we are interested in large changes, the chosen sample size was deemed appropriate. Sample size for experiments is mentioned in the figure captions.
Data exclusions	No data was excluded.
Replication	To ensure reproducibility, most experiments were repeated twice (and often more) with similar results. Replication was also performed between different experiments. The number of times an experiment was repeated is mentioned in the figure captions, this number does not include part of the experiment repeated between experiments. Reproducibility was also demonstrated by performing multiple distinct experiments to support conclusions made in the manuscript.
Randomization	Randomization is not relevant to this study as samples were not divided into experimental groups.
Blinding	Blinding was not applicable because these types of studies do not use group allocation.

Reporting for specific materials, systems and methods

We require information from authors about some types of materials, experimental systems and methods used in many studies. Here, indicate whether each material, system or method listed is relevant to your study. If you are not sure if a list item applies to your research, read the appropriate section before selecting a response.

Materials & experimental systems

n/a	Involved in the study	
☐	☒	Antibodies
☐	☒	Eukaryotic cell lines
☒	☐	Palaeontology and archaeology
☒	☐	Animals and other organisms
☒	☐	Human research participants
☒	☐	Clinical data
☒	☐	Dual use research of concern

Methods

n/a	Involved in the study	
☒	☐	ChIP-seq
☒	☐	Flow cytometry
☒	☐	MRI-based neuroimaging

Antibodies

Antibodies used	Anti-6x-His tag antibody (Thermo Fisher, HIS.H8, #MA1-21315). Anti-mouse IgG, HRP-linked Antibody (Cell signal, #7076).
Validation	The validation of primary antibody Anti-6x-His tag antibody (Thermo Fisher, HIS.H8, #MA1-21315) is described as following by the supplier: " This Antibody was verified by Relative expression to ensure that the antibody binds to the antigen stated. Antibody specificity was demonstrated by detection of different targets fused to 6x-His tag in transiently transfected lysates tested. Relative detection of 6x-His tag was observed across different proteins fused with 6x-His in V5-H3-His (Lane 3) and His-p65-YFP (Lane 4-6), using Anti-6x-His Tag Monoclonal Antibody (HIS.H8)" In addition, we have tested this antibody for detection of His-tagged proteins in western blots and on-cell assays." The manufacture's website also mentions that the Anti-6x-His tag antibody has been mentioned in 170 references.<br>We have also tested the secondary antibody Anti-mouse IgG, HRP-linked Antibody (Cell signal, #7076) for detection of mouse IgG in western blots and on-cell assays.<br>For on-cell assay, primary antibody (Anti-6x-His tag) was used at a dilution of 1:1000 in PBST (1% milk powder) and secondary antibody (Anti-mouse IgG, HRP-linked) was used at a dilution of 1:1500 in PBST (1% milk powder).<br>Note that both primary and secondary antibodies have been used to detect 6xHis-tagged proteins in our on-cell assays and western blots shown in figures 2a, 2g, 5a and 5d.

Eukaryotic cell lines

Policy information about cell lines

Cell line source(s)	Human epithelial squamous carcinoma cell line, A-431 (Catalog no. 85090402); Human breast adenocarcinoma cell line, MDA-MB-231 (Catalog no. 92020424) and Human colon adenocarcinoma cell line, SW620 (Catalog no. 87051203) were purchased from Sigma-Aldrich, now Merck.
Authentication	Cell lines were obtained from Sigma-Aldrich, now Merck, and no further authentication was done.
Mycoplasma contamination	Cell lines were tested for Mycoplasma using a PCR method showing they were mycoplasma free.
Commonly misidentified lines (See ICLAC register)	We could not find "A-431" or "MDA-MB-231" or "SW620" on the Register of Misidentified Cell Lines accessed through the following website: https://iclac.org/databases/cross-contaminations/
