# Supplementary figures and images for: Site-specific encoding of photoactivity and photoreactivity into antibody fragments
Source: Nat Chem Biol. 2023 Feb 16;19(6):740–9. doi: 10.1038/s41589-022-01251-9 (PMC10229432; doi:10.1038/s41589-022-01251-9)

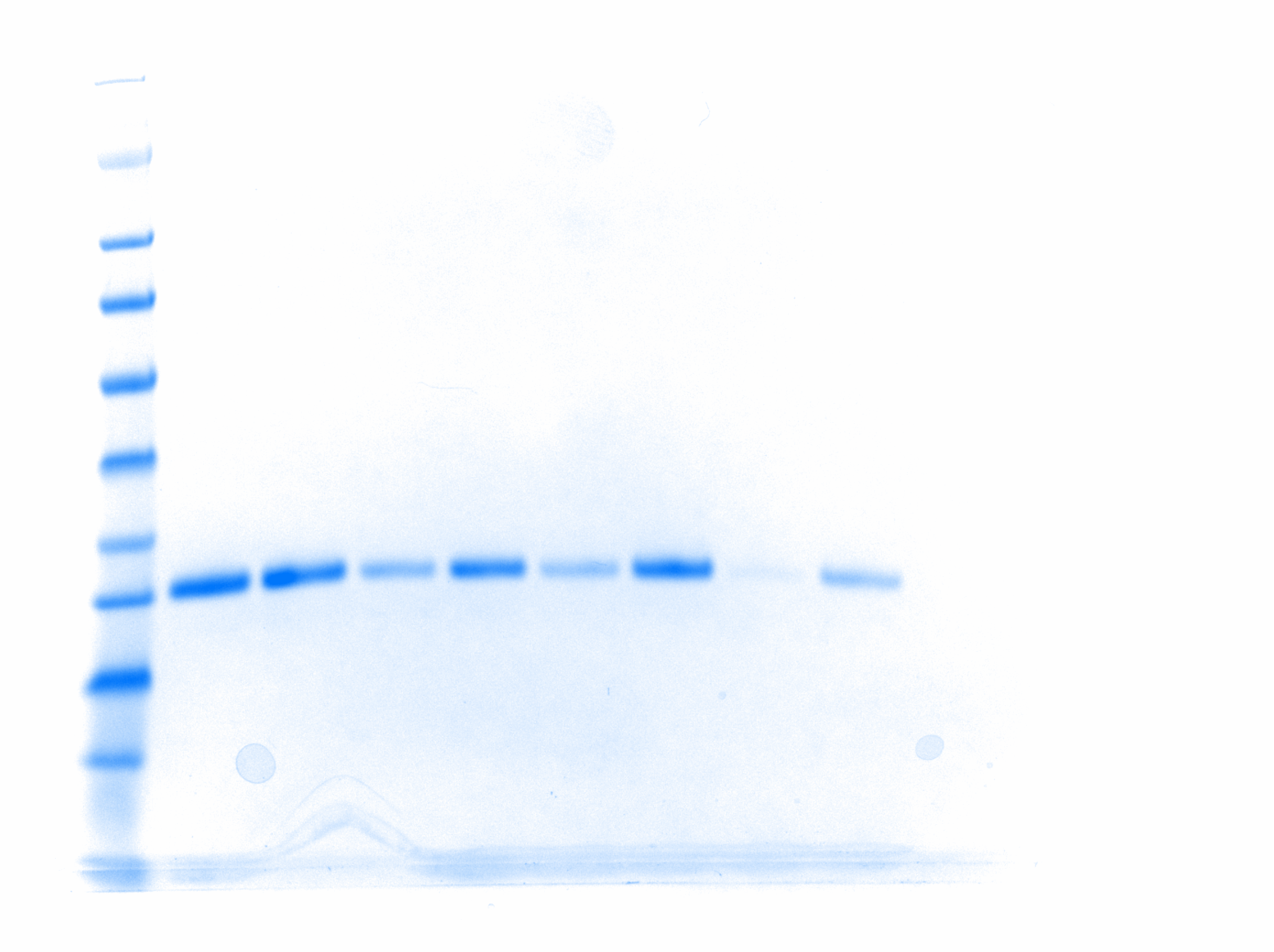

Supplement: Source Data Fig. 1 — Full-length Coomassie-stained SDS–PAGE gel in Fig. 1. [file 41589_2022_1251_MOESM3_ESM.tif]

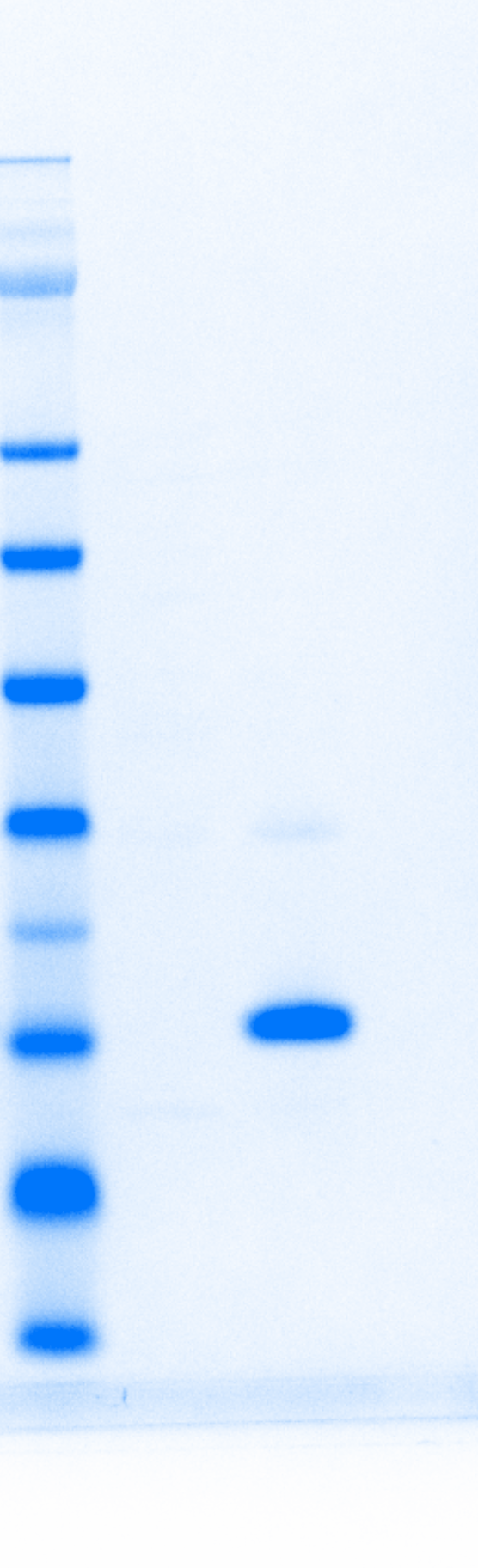

Supplement: Source Data Fig. 4 — Full-length Coomassie-stained SDS–PAGE gel in Fig. 4. [file 41589_2022_1251_MOESM8_ESM.tif]

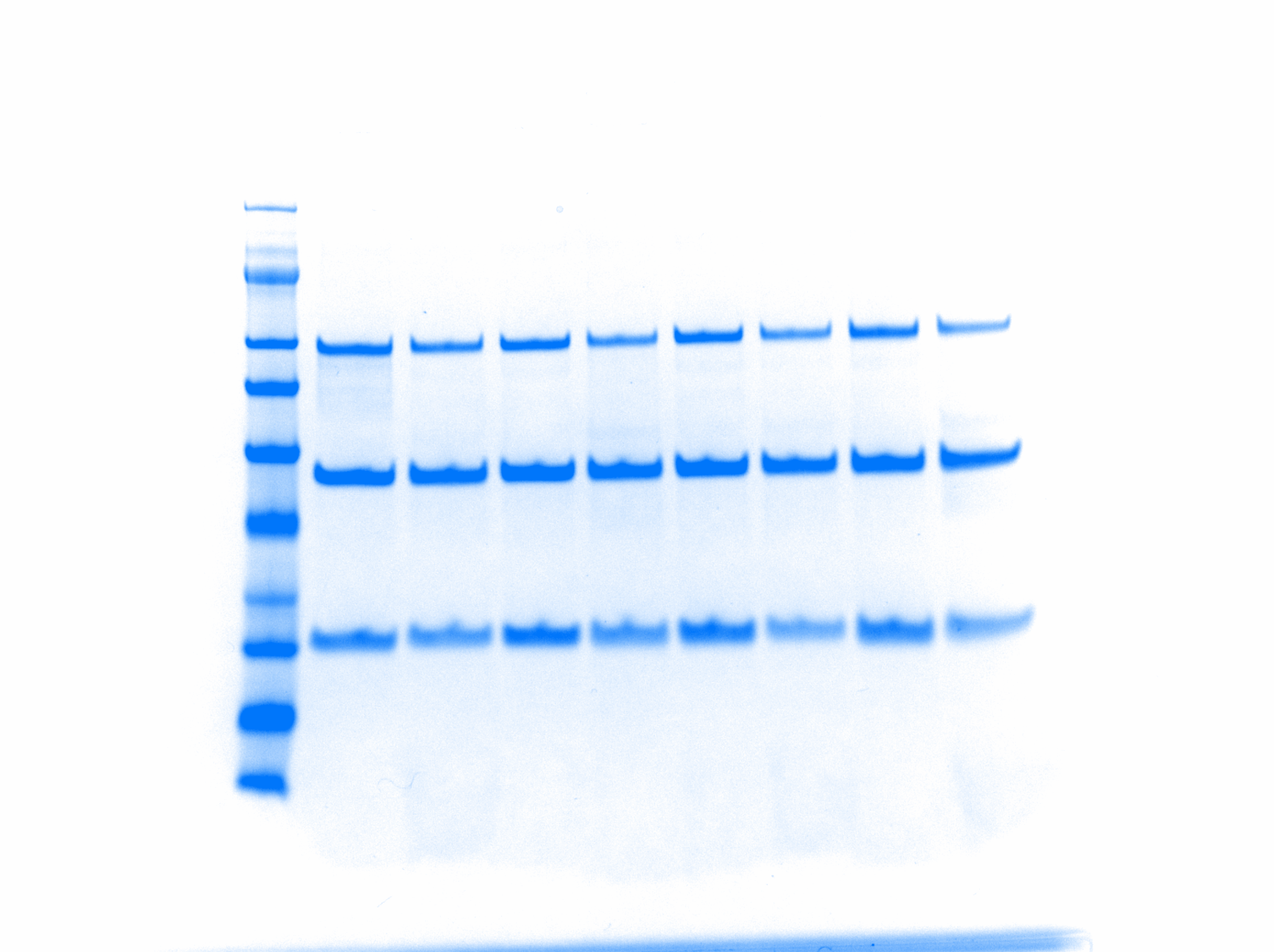

Supplement: Source Data Extended Data Fig. 5 — Full-length Coomassie-stained SDS–PAGE gel in Extended Data Fig. 5. [file 41589_2022_1251_MOESM15_ESM.tif]

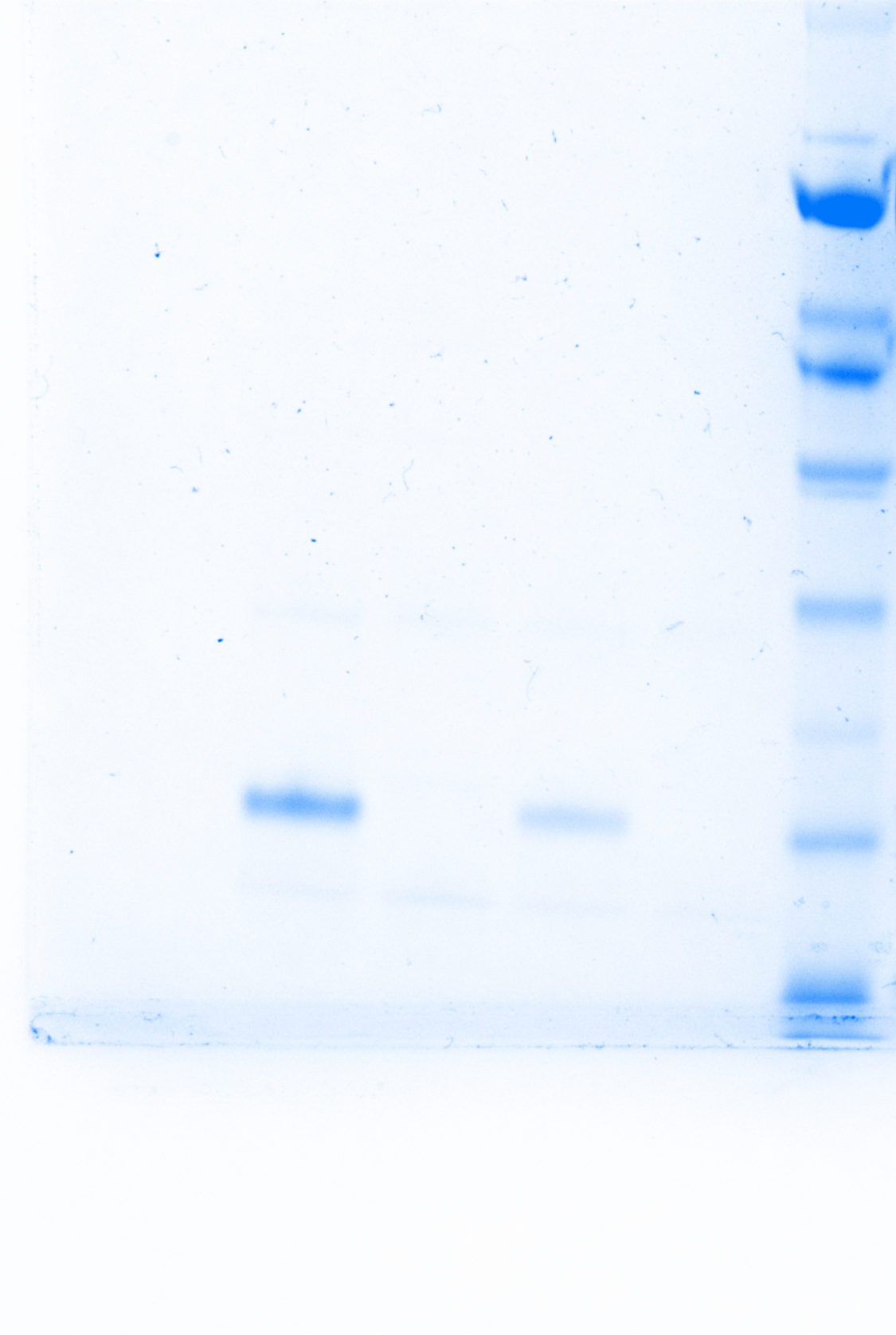

Supplement: Source Data Extended Data Fig. 7 — Full-length Coomassie-stained SDS–PAGE gel in Extended Data Fig. 7. [file 41589_2022_1251_MOESM17_ESM.tif]
