# Supplementary material for: Site-specific encoding of photoactivity and photoreactivity into antibody fragments
Source: Nat Chem Biol. 2023 Feb 16;19(6):740–9. doi: 10.1038/s41589-022-01251-9 (PMC10229432; doi:10.1038/s41589-022-01251-9)

Source data for Figure 3 showing full length gel images:

Source data for Figure 3c:

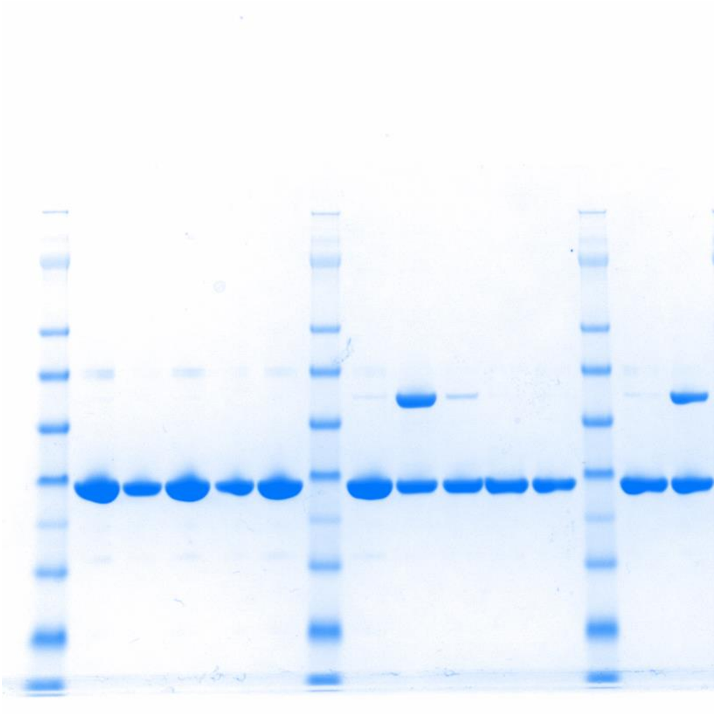

Source data for Figure 3d (Left Panel):

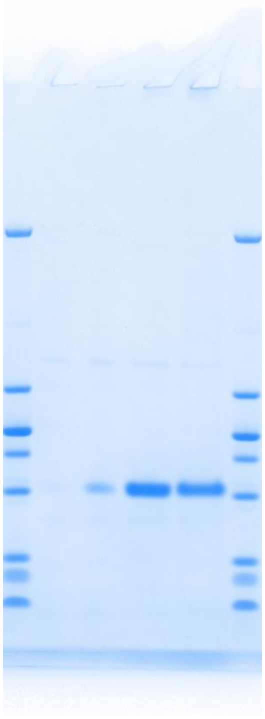

Source data for Figure 3d (Right Panel):

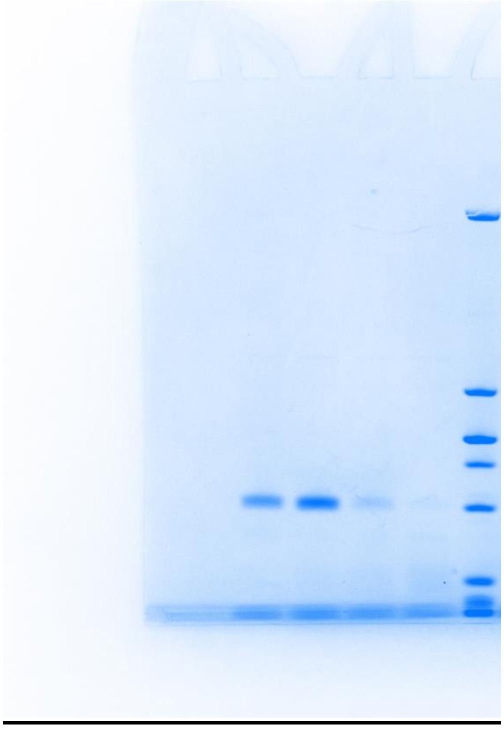

Supplement: Source Data Fig. 3 — Full-length Coomassie-stained SDS–PAGE gel in Fig. 3. [file 41589_2022_1251_MOESM6_ESM.pdf]
