# Supplementary material for: Site-specific encoding of photoactivity and photoreactivity into antibody fragments
Source: Nat Chem Biol. 2023 Feb 16;19(6):740–9. doi: 10.1038/s41589-022-01251-9 (PMC10229432; doi:10.1038/s41589-022-01251-9)

Source data for Extended Data Figure 6 showing full length gel images:

Source data for Extended Data Figure 6- Left Panel, Coomassie Stained gel:

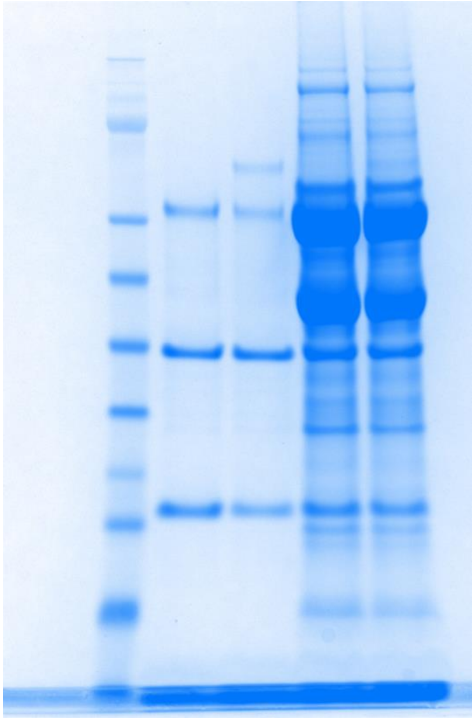

Source data for Extended Data Figure 6- Right Panel, Western blot image:

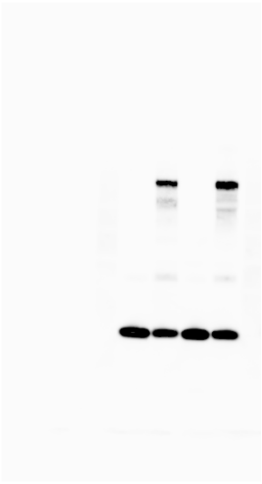

Source data for Extended Data Figure 6- Right Panel, Western blot image taken using white light for protein ladder:

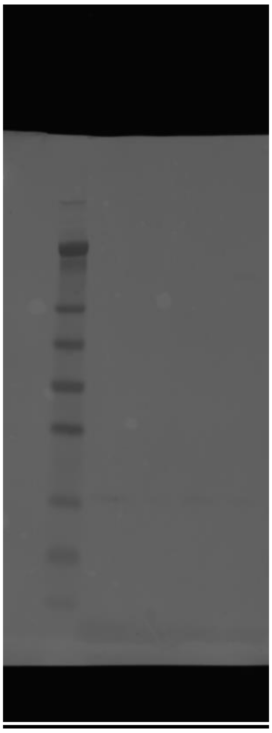

Supplement: Source Data Extended Data Fig. 6 — Full-length SDS–PAGE gel and blots in Extended Data Fig. 6. [file 41589_2022_1251_MOESM16_ESM.pdf]
